# Oscillatory co-expression of HES1 and HES5 enables a hybrid state in a cross-repressive transcription factor regulatory motif

**DOI:** 10.1242/dev.204969

**Published:** 2026-06-10

**Authors:** Veronica Biga, Anzy Miller, Anoushka Kamath, Robert Lea, Ying Q. P. Mak, Antony D. Adamson, Elli Marinopoulou, Paul François, Nancy Papalopulu, Cerys S. Manning

**Affiliations:** ^1^ Faculty of Biology Medicine and Health, School of Medical Sciences, Division of Developmental Biology and Medicine, The University of Manchester, Manchester M13 9PT, UK; ^2^ Faculty of Biology Medicine and Health, Division of Cardiovascular Sciences , University of Manchester, Manchester M13 9PT, UK; ^3^ UK Dementia Research Institute, University College London, London WC1E 6BT, UK; ^4^ Genome Editing Unit, Faculty of Biology, Medicine and Health, The University of Manchester, Manchester M13 9PT, UK; ^5^ Research Development and Innovation, Faculty of Biology Medicine and Health, The University of Manchester, Manchester M13 9PT, UK; ^6^ Department of Biochemistry and Molecular Medicine, University of Montreal, Montreal, Quebec QC H3T 1J4, Canada

**Keywords:** Oscillatory dynamics, Transcription factor regulation, Cross-repressing transcription factors

## Abstract

Many cell fate decisions in the developing neural tube are directed by cross-repressive transcription factor (TF) motifs that generate bistability, such that cells express one TF but not both. Hybrid states in which cells express both cross-repressing fate determinants have been observed, but how these arise or persist remains unclear. Here, we focus on HES1 and HES5, which are auto-repressive oscillatory TFs that regulate neural progenitor maintenance and are expressed in adjacent dorsoventral progenitor domains in the developing spinal cord. Knockdown experiments demonstrate that HES1 and HES5 are cross-repressing in mouse spinal cord neural progenitors, and live-cell imaging *in vitro* shows that they can be co-expressed, defining a hybrid state. In this state, HES proteins co-oscillate in-phase within single cells. Computational modelling indicates that modulation of cross-repression strength or relative TF abundance destabilises this state, driving resolution towards a single oscillatory HES TF. This is consistent with *in vivo* analysis showing transient HES1 and HES5 co-expression, followed by progressive restriction to a single TF oscillator. Our findings suggest that oscillatory expression enables the co-existence of cross-repressing TFs, allowing hybrid states within a developmental bistable motif.

## Teaser

**Highlighted Article:** Expression of the cross-repressing proteins HES1 and HES5 is mutually exclusive but when they oscillate, a cell can express both, creating a hybrid state that later resolves into single HES states.

## INTRODUCTION

In the developing neural tube, progenitor cells make decisions to differentiate into specialised cell types, governed by gene expression programmes that define specific cell fates. Along the dorsoventral axis of the mammalian spinal cord, the complexity of cell types arises from progenitor domains that are reproducibly organised in distinct domains of gene expression. These arise when homeodomain transcription factors (TFs), such as Pax6, Olig2 and Nkx2.2, are differentially induced by morphogenetic factors and then cross-repress each other in adjacent progenitor domains, reinforcing distinct gene expression territories (Briscoe et al., 2000; Balaskas et al., 2012; Sagner and Briscoe, 2019). This domain-specific expression of TFs, reflecting mutually exclusive cell fates, represents several bistable transcriptional switches between TFs in adjacent domains, which together comprise an extensive gene regulatory network. In this case, bistability, a system in which cells can stably adopt one of two gene expression states, is mediated by cross-repression between fate-determining TFs, where cells can express gene A or B, but not both (Ferrell, 2002). Bistability enables cells to switch between discrete states and is a common mechanism for enforcing binary fate decisions in development (Moris et al., 2016; Kutejova et al., 2016). Thus, domain boundaries, which initially contain a mix of cells of different fates (expressing different genes) are sharpened through resolution of bistable TF expression combined with cell re-arrangement, ensuring stable region-specific cell fates along the dorsoventral axis.

In traditional or idealised models of bistability, intermediate states in which cells express both gene A and B are unstable, with abrupt transitions between A+/B− and A−/B+ states (Wang et al., 2009; Verdugo et al., 2013; Zorzan et al., 2021; Rombouts and Gelens, 2021). However, during development, there is growing evidence to suggest that, before fates are resolved, progenitor cells can occupy a transitory or ‘hybrid’ state, in which genes associated with opposing fates are co-expressed in the same cells, as discussed by Moris et al. (2016). For example, in early embryonic development, co-expression of pluripotency markers (e.g. OCT4) and TFs associated with lineage commitment, such as GATA6, MIXL1 and Hex, as well as PDGFRa, has been reported (Plusa et al., 2008; Morgani et al., 2013; Allison et al., 2018; Stavish et al., 2020; Redo-Riveiro et al., 2024). In the developing nervous system, we detected co-expression of progenitor and differentiation genes by smFISH in the zebrafish hindbrain (Soto et al., 2020). scRNAseq is also increasingly identifying intermediate or hybrid states in many different contexts where cell fate decisions are made, including haematopoiesis (Hu et al., 1997; Laslo et al., 2006; Olsson et al., 2016; Bergiers et al., 2018), osteogenesis (Wang et al., 2009), T cell differentiation (Zhou et al., 2008) and the epithelial-mesenchymal transition, reviewed by MacLean et al. (2018). These have spurred new computational approaches to determine the underlying network structure (Dey and MacLean, 2025), as well as identifying such states from data (Kong et al., 2022).

**Fig. 1.**
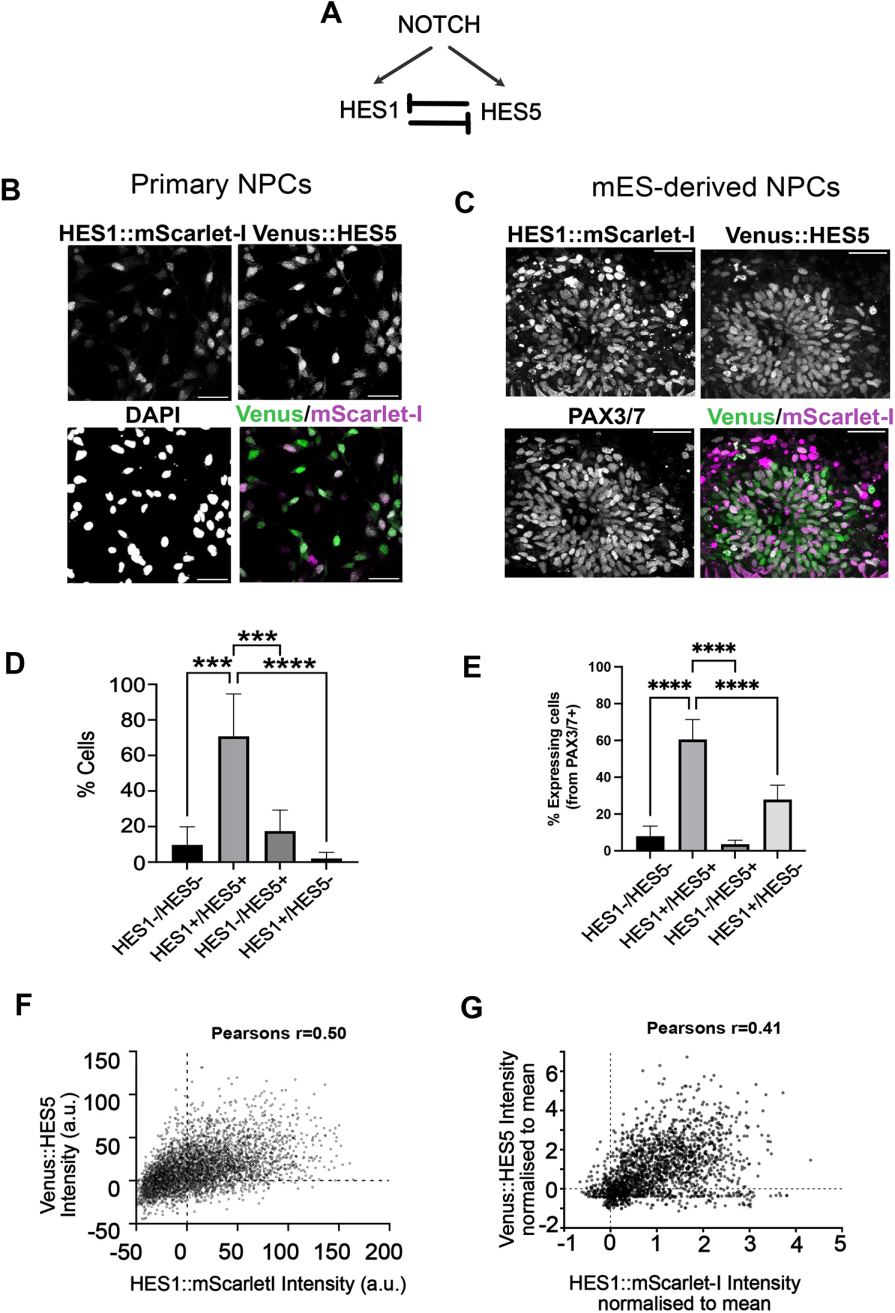
**Co-expression of HES1 and HES5 in primary spinal cord and mouse embryonic stem cell-derived neural progenitor cells.** (A) Diagram of Notch activating the HES1 and HES5 targets that cross-repress. (B) Fixed primary neural progenitor cell cultures expressing endogenous HES1::mScarlet-I, endogenous Venus::HES5 and a nuclear DAPI stain at 2 days in culture; 40× objective. Scale bars: 40 μm. (C) Fixed mouse embryonic stem cell (mESC)-derived dorsal progenitor cell cultures at 2 days post-retinoic acid removal (see Materials and Methods) expressing endogenous HES1::mScarlet-I, endogenous mVenus::HES5 and immunofluorescence for PAX3/7 antibody; 40× objective. Scale bars: 40 μm. (D,E) Quantification of single- and double-positive HES1::mScarlet-I and Venus::HES5 fractions observed in primary neural progenitor cells (priNPCs) (D) and mESC-derived NPCs (E). Data are mean±s.d. D, *n*=4 experiments, >2000 cells analysed; E, *n*=3 experiments, 1987 cells; one-way ANOVA, Tukey's multiple comparison correction (***P<0.001; ****P<0.0001). (F,G) Scatter plots of HES1::mScarlet-I versus Venus::HES5 in the same cell obtained from priNPC (F) mESC-derived NPC cultures (G). Markers indicate background-subtracted mean fluorescent intensity per nucleus; normalised data are divided by the mean per experiment.

This presents a conceptual challenge: how can cross-repressing TFs co-exist within the same cell, and how is this hybrid state eventually resolved into a stable mutually exclusive fate choice? A few theoretical explanations have been proposed, including narrow parameter regimes that permit co-expression (Andrecut et al., 2011), weak (Laslo et al., 2006; Rohm et al., 2018) or delayed cross-repression (Zhu et al., 2007; Bokes et al., 2009), strong autoregulation (Guantes and Poyatos, 2008; Wu et al., 2017) and noise from transcriptional bursting (Bokes et al., 2013). In theoretical models of spinal cord patterning, the network structure and dynamics of cross-repressive (toggle) switches allow hybrid states to be observed transiently (Perez-Carrasco et al., 2016; Exelby et al., 2021). Furthermore, modelling has suggested that oscillations can lead to co-expression in a bistable switch comprising competitive TFs (Bokes and King, 2019). However, experimental evidence is lacking. We hypothesised that oscillatory expression of TFs within the same cell may allow progenitor cells to co-express cross-repressing fate determinants, offering a mechanistic basis for the observed hybrid progenitor state, while at the same time allowing the fates to be resolved over time. To test this hypothesis in the context of spinal cord development, and to gain some mechanistic insight, we focused on hairy and enhancer of split (HES) 1 and 5, members of the basic helix-loop-helix (bHLH) family of transcriptional repressors, because they are known to repress each other (cross-repress) (Fig. 1A) as well as to oscillate via auto-repression in neural progenitors during neural development. Evidence for cross-repression comes from single knockout mouse models, where loss of *Hes1* leads to *Hes5* becoming expressed throughout spinal cord; conversely, loss of *Hes5* causes *Hes1* to be upregulated in areas where *Hes5* would normally be highly expressed (Hatakeyama et al., 2004). Furthermore, over-expression of *Hes5* in mouse embryos leads to a decrease in *Hes1* expression and, conversely, a Hes5-GFP BAC reporter is upregulated in *Hes1* germline mutants (Bansod et al., 2017; Riesenberg et al., 2018).

The Notch target genes HES1 (including zebrafish ortholog Her6) and HES5 have been shown to have temporal oscillatory expression across different species and tissue types including muscle (Lahmann et al., 2019), pancreas (Seymour et al., 2020), the mouse spinal cord (Manning et al., 2019; Biga et al., 2021; Hawley et al., 2022), forebrain (Imayoshi et al., 2013; Ochi et al., 2020), the zebrafish brain (Soto et al., 2020; Doostdar et al., 2024) and pre-somitic mesoderm (Niwa et al., 2007; Shimojo et al., 2016; El Azhar et al., 2024). HES oscillations are typically in the order of a few hours (Kageyama et al., 2007; Manning et al., 2019; Soto et al., 2020; Doostdar et al., 2024) and are generated via delayed auto-negative feedback (Takebayashi et al., 1994; Kageyama et al., 2007), combined with short mRNA and protein half-lives (Hirata et al., 2002; Imayoshi et al., 2013). Double HES knockouts show that their expression is required for CNS development and, importantly, for neural progenitor maintenance (Hatakeyama et al., 2004; Bansod et al., 2017). Experiments involving characterisation and manipulation of dynamics show that their oscillatory expression is important for cell state transitions (Manning et al., 2019; Soto et al., 2020; Marinopoulou et al., 2021; Maeda et al., 2023; Shimojo et al., 2024). This indicates that oscillatory HES proteins represent a developmental programme responsible for enabling state transitions in the developing CNS, including spinal cord. Much like the homeodomain TFs mentioned above, HES1 and HES5 are thought to occupy mutually exclusive adjacent territories along the dorsoventral axis of the developing spinal cord (Sagner et al., 2018). When combined with single HES mouse knockout data, this segregated spatial localisation seems to be due to cross-repression (Hatakeyama et al., 2004).

To understand whether oscillatory dynamics may allow co-expression but also resolution of cross-repressive TFs, we performed simultaneous live imaging of HES1 and HES5 in single cells, using genetically engineered reporters for endogenous expression dynamics. Knockdown experiments confirmed that HES1 and HES5 are indeed cross-repressing TFs. We observed co-oscillation of HES1 and HES5 in neural progenitor cells *in vitro*, and co-expression *in vivo*, particularly during early developmental stages. Computational modelling based on auto- and cross-repression predicted that HES1 and HES5 have different free running periods, explained *in silico* by differential protein stability. However, when HES1 and HES5 were co-expressed in the same cells, the two oscillators became in-phase, suggesting a correlation between co-expression and changes in periodicity, particularly a slowing down of the faster oscillator (HES1). Changing the cross-repression strength or the level of either protein *in silico*, allowed one oscillator to prevail and switch off the other, which may underlie the resolution of expression into mutually exclusive domains during development of the spinal cord. Our findings show that the simultaneous oscillatory expression of HES1 and HES5 enables progenitor cells to co-express cross-repressing fate determinants, before resolving to progenitors with mutually exclusive oscillatory expression. This provides a mechanistic basis for the maintenance of a hybrid progenitor state through in-phase oscillations. Furthermore, our data provide a mechanistic link between oscillatory expression and fate resolution, and reveal additional features of HES oscillations, including in-phase expression and the extension of the decision-making window, potentially enhancing both robustness and flexibility in neural development.

## RESULTS

### *In vitro* neural progenitor cultures indicate HES1 is co-expressed with HES5

First, we defined progenitor cell states according to whether they express HES1 or HES5, or both. To do this we characterised the endogenous expression using previously described knock-in fluorescent reporters (Imayoshi et al., 2013; Marinopoulou et al., 2021) in both primary neural progenitor cells (priNPCs) derived from E10.5 spinal cord (Venus::HES5^+/−^, HES1::mScarlet-I^+/−^) and mouse embryonic stem cell (mESC)-derived neural progenitor cells (NPCs) (Venus::HES5^+/+^, HES1::mScarlet-I^+/+^) (Fig. 1B-G, Fig. S1). A large proportion, representing 60-65% of cells, co-express HES1 and HES5 in primary and mESC-derived NPCs, respectively (Fig. 1D,E). We also observed that 28% of NPCs expressed HES1 only in the mESC differentiation protocol, which enriches for dorsal types of interneuron progenitors (Gupta et al., 2022) (identified by expression of PAX3/7; Mansouri and Gruss, 1998), where HES1 is more widely expressed compared to ventral types (Sagner et al., 2018). Both the co-expressing and single HES1 fractions were observed in mESC-derived progenitors over several differentiation days (Fig. S1B). HES1 and HES5 intensities in the same cell were positively correlated, which is indicative of co-expression at a range of levels (Fig. 1F,G). These results indicated that a ‘hybrid state’ exhibiting co-expression of HES1 and HES5 is observed in most spinal cord NPCs *in-vitro*.

### HES1 and HES5 oscillate at a similar period, although HES1 is more oscillatory and has a higher amplitude than HES5

**Fig. 2.**
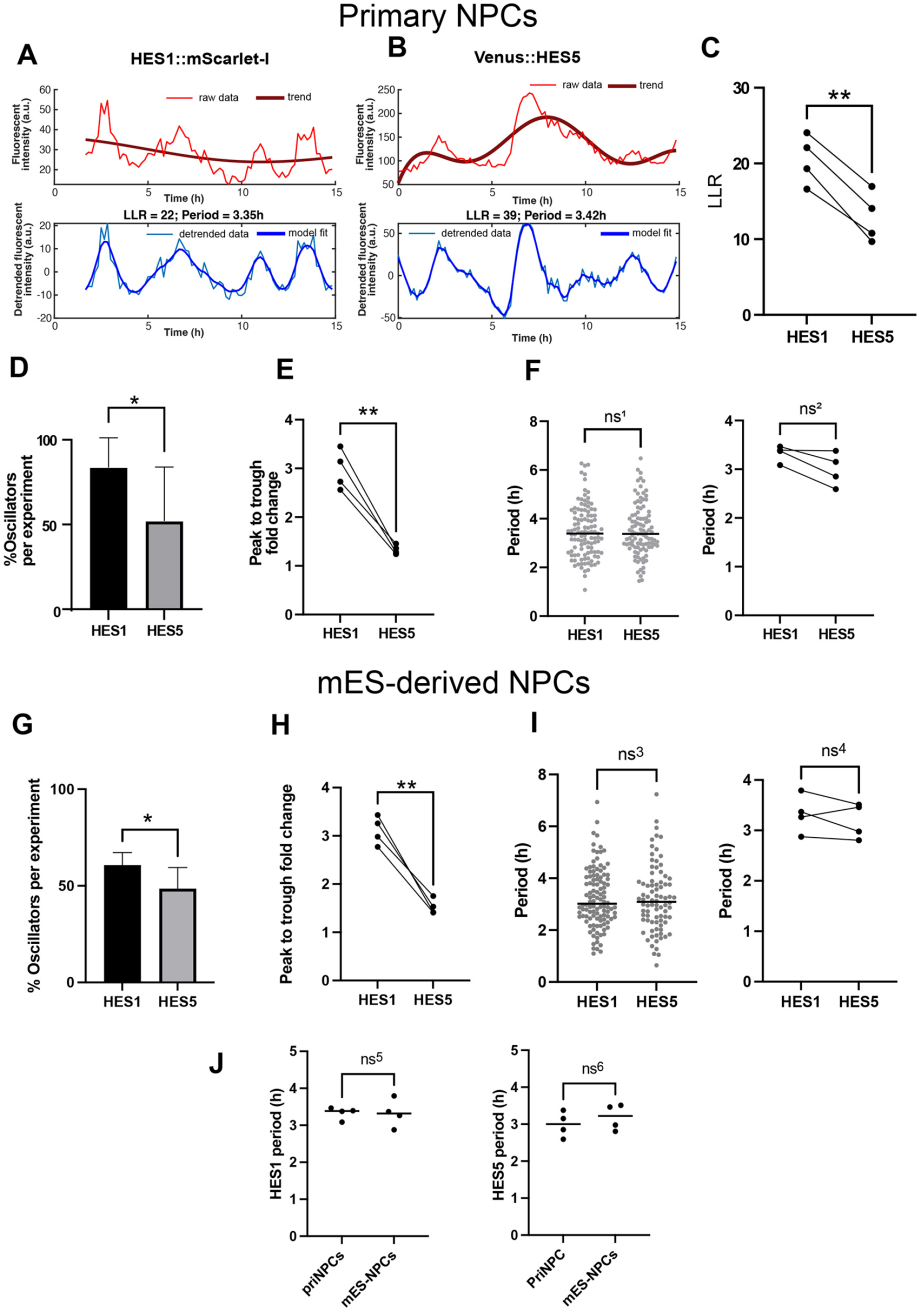
**Oscillatory expression of HES1 and HES5 in primary spinal cord and mouse embryonic stem cell-derived neural progenitor cells.** (A,B) Representative examples of periodic fluctuations observed in mean nuclear intensity of HES1::mScarlet-I (A) and Venus::HES5 (B) in two different primary neural progenitor cells (priNPCs) over time. Upper panels indicate raw intensity and slow-varying trend (solid red line); lower panels indicate detrended intensity and periodic model fit (solid blue line). LLR denotes log-likelihood ratio. (C) Comparison of LLR values (indicative of oscillation quality) observed in HES1::mScarlet-I versus Venus::HES5 timeseries. Data points indicate the median per experiment. (D) Percentage of oscillatory timeseries observed in HES1::mScarlet-I and Venus::HES5. Data are mean±s.d. (E) Comparison of maximum peak-to-trough fold change in fluorescent intensity per cell in HES1::mScarlet-I versus Venus::HES5 timeseries. Data points indicate the median per experiment. (C-E) Two-tailed paired *t*-tests (*P<0.05; **P<0.01); sample size, *n*=4 experiments, 446 tracks. (F) Comparison of period duration in oscillatory HES1::mScarlet-I versus Venus::HES5 timeseries observed in a single experiment as well as across multiple repeats. Left: data points are values from individual cells; bars indicate the median. Two-tailed Mann–Whitney test (ns^1^, non-significant; *P*=0.7065). Right: data points indicate the median per experiment. Two-tailed paired *t*-test (ns^2^, non-significant; *P*=0.0639). (G) Percentage of oscillatory timeseries observed in HES1::mScarlet-I and mVenus::HES5 mouse embryonic stem cell (mESC)-derived NPCs. Data are mean±s.d. Two-tailed paired *t*-test (*P<0.05). Sample size for G-I: *n*=4 experiments, 220 tracks. (H) Comparison of maximum peak-to-trough fold change in mESC-derived progenitors; data points indicate the median per experiment. Two-tailed paired *t*-test (**P<0.01). (I) Comparison of period duration in oscillatory HES1-mScarlet-I versus mVenus::HES5 timeseries in mESC-derived NPCs. Left: data points are values from individual cells; bars indicate the median. Two-tailed Mann–Whitney test (ns^3^, non-significant; *P*=0.7443). Right: data points indicate the median per experiment. Two-tailed paired *t*-test (ns^4^, non-significant; *P*=0.3721). (J) Comparison of HES1 and HES5 period in different culture systems. Data points indicate the median per experiment. Two-tailed unpaired *t*-test [ns^5^, non-significant; (*P*=0.9851); ns^6^, non-significant (*P*=0.4543)].

We compared the oscillatory parameters of HES1 and HES5 in NPCs using timelapse imaging and tracking of individual nuclei (Fig. 2). We observed that both proteins have peaks of expression over time (Fig. 2A,B, examples from two progenitors) with HES1 expression dynamics being more regular and periodic compared to HES5 [Fig. 2C, log likelihood ratio (LLR) indicative of oscillation quality, see Phillips et al., 2017]. A higher proportion of HES1 timeseries passed a statistical test for oscillatory expression compared to HES5 timeseries (Fig. 2D). In addition, the dynamics of HES1 expression were more pronounced compared to HES5, quantified as the ratio of intensity at the peak versus intensity at the trough (Fig. 2E, peak-to-trough fold change).

As the two proteins were monitored with different fluorophore fusions (HES1 fused to mScarlet-I and HES5 fused to Venus), we validated that the period and fold-change measurements were not significantly affected by choice of fluorophore. To do this, we generated a HES1::mVenus mouse (see Materials and Methods), crossed it with the existing HES1::mScarlet-I mouse and derived priNPC cultures containing a double knock-in of HES1 fused with mVenus on one allele and mScarlet-I on the other (Fig. S2A). Periodic activity with in-phase oscillations on each HES1 allele and no differences in the period, maximum or mean fold-change values was observed with the double HES1 reporters (Fig. S2B-F). Additionally, we found that the HES5 period was unchanged in homozygous versus heterozygous Venus::HES5 cultures (Fig. S2G). These observations argue against artefactual differences due to the choice of fluorophores or mono-allelic versus bi-allelic expression, and confirm that HES1 is more oscillatory and has a higher fold-change compared to HES5 in priNPCs (Fig. 2D,E).

The period of oscillations was variable at the single cell level, approximately 3.3 h for HES1 and 3 h for HES5 in priNPCs, with no significant differences at the population level (Fig. 2F). In mESC-derived NPCs, we observed similar features of the dynamics of HES1 and HES5 expression (Fig. 2G-I). The HES period showed no significant differences between the two culture systems (Fig. 2J), even though mESC-derived NPCs grow as rosettes and have a higher local density than priNPCs (Movies 1 and 2, respectively). Overall, we conclude that HES1 is more oscillatory and has a higher peak-to-trough fold change but, on average, has the same period as HES5 at the population level.

### HES1 and HES5 oscillate in-phase in the co-expressing fraction

**Fig. 3.**
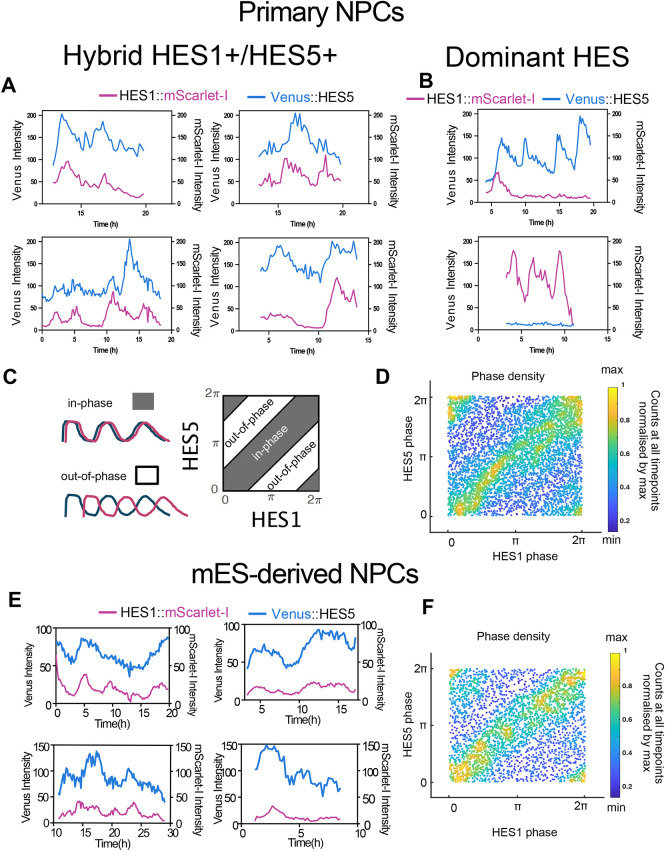
**Exploration of synchrony in HES1 and HES5 oscillations in the same cells.** (A,B) Representative examples of HES1::mScarlet-I (magenta) and Venus::HES5 (teal) raw intensity timeseries in the same cell observed in neural progenitor cell (priNPC) cultures in the same experiment grouped into co-expressing (or hybrid HES1^+^/HES5^+^ in A) and dominant HES in B; fluorescence units of Venus and mScarlet-I are not directly comparable; however, the same fluorophore intensity can be compared across examples. (C) Diagram depicting areas of HES1 versus HES5 in in-phase and out-of-phase regions of phase-phase density mapping. (D) Phase-phase density mapping showing HES1 and HES5 phase values observed in the same cells at the same time in timeseries collected from priNPCs; markers are colour-coded to indicate low- and high-probability density areas. (E) Representative examples of raw timeseries of HES1-mScarlet-I and mVenus::HES5 observed in mESC-derived NPCs. (F) Phase-phase density mapping showing HES1 and HES5 phase values observed in timeseries collected from mESC-derived NPCs; markers are colour-coded to indicate low- and high-probability density areas.

Next, we monitored the fluctuations in level of both Venus::HES5 and HES1::mScarlet-I in the same cells over time using detrended data (Fig. 3, see Materials and Methods). The majority of progenitors co-expressed HES1 and HES5 and showed positively correlated dynamics at the single cell level in priNPCs, indicating the presence of coordinated activity in the same cells; however, dynamics were uncorrelated between different cells (Fig. 3A-D, Fig. S3A,B). Notably, dynamics with negative correlations were also observed, albeit less frequently, corresponding to one HES oscillating while expression of the other is very low, a regime that we refer to as dominant (Fig. 3B, Fig. S3A).

The presence of correlated intra-cellular HES1 and HES5 in both culture systems (Fig. 3A,E) was consistent with the predominance of the hybrid HES1+/HES5+ cell state at a fixed timepoint (Fig. 1D,E) and, in addition, strongly suggested synchronisation of HES oscillations. To confirm synchrony, we analysed the phase relationship of HES1 and HES5 oscillations in the hybrid HES1+/HES5+ cell state, where phase refers to the position within the oscillation cycle at any given time (Fig. S3C). We used phase-phase mapping according to previously described techniques (Biga et al., 2021), where the phase of HES1 versus the phase of HES5 would be synchronous if the data clusters were along the first diagonal and at opposite corners (Fig. 3C).

The phase relationship between HES1 and HES5 varied between cells, and also in the same cell over time (Fig. S3D). However, despite this presence of stochasticity in phase, over 80% of co-expressing cells showed synchronous HES1 and HES5 oscillations (Fig. 3D,F, Fig. S3E).

Taken together, the data indicate that HES1 and HES5 oscillate in-phase in the hybrid HES1+/HES5+ cell state, thus providing a way to balance the expression of two TFs in a dynamic way over time. This prompted the need to determine the mechanism leading to synchronisation.

### HES1 and HES5 are predicted to have distinct ‘free-running periods’

**Fig. 4.**
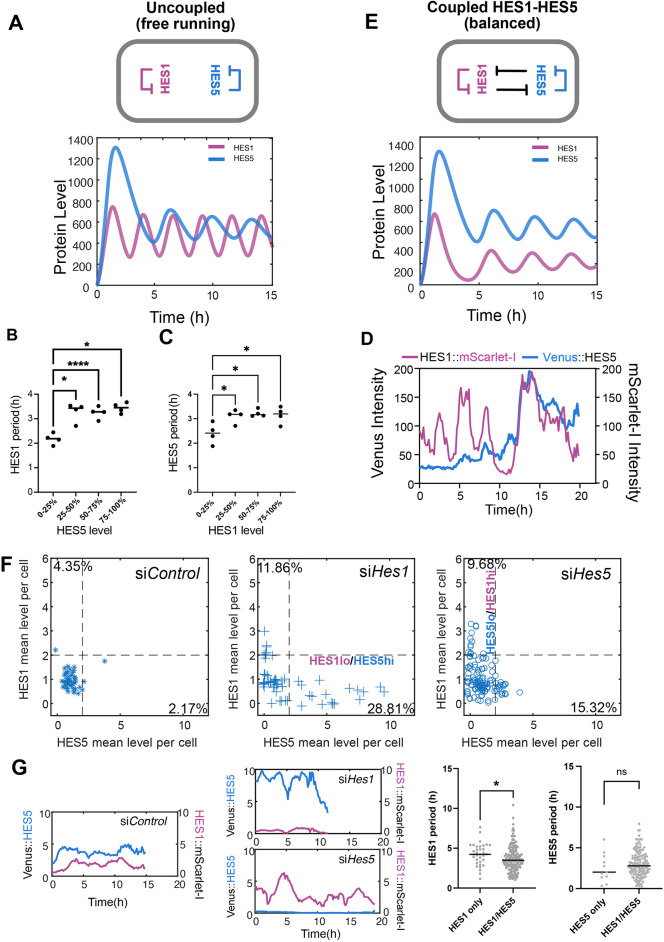
**A mathematical model of coupled HES1-HES oscillations explains how cells can adopt a common period through bidirectional effects.** (A) Simulation of free running (uncoupled) HES1 and HES5 dynamics at fixed values of period and mRNA degradation (see Materials and Methods). HES1 rates: 
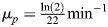
, 
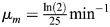
; HES5 rates: 
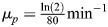
, 
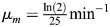
; other model parameters: 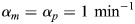; 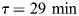; *n*=7; *P*_0_=390. (B,C) HES1 (B) and HES5 (C) period observed in primary neural progenitor cells (priNPCs) with different levels of HES5 (B) and HES1 (C). Protein levels were binned into quartiles and the median per quartile/per experiment was reported; repeated measures ANOVA with Dunnet's multiple comparison correction (*P<0.05; ****P<0.0001). (D) Representative example of priNPCs showing a transition from single HES1 expressing to HES1^+^/HES5^+^ co-expressing over time. The HES1 period increases in the co-expressing state. (E) Simulation of coupled HES1 and HES5 dynamics at the same values of cross-repression (HES1 onto HES5, or HES5 onto HES1) and self-repression threshold (HES1 onto HES1; HES5 onto HES5), referred to as balanced coupling: *P*_015_=*P*_051_=*P*_01_=*P*_05_=390; HES1 rates: 
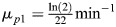
, 
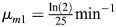
; HES5 rates: 
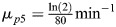
, 
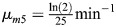
; other model parameters: 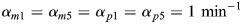; 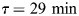; *n*=7; *n*_15_=*n*_51_=5. (F) Perturbations in the level of either HES protein observed in mouse embryonic stem cell-derived NPCs under siRNA knockdown against *Hes1* (si*Hes1*), *Hes5* (si*Hes5*) and non-targeting control (si*Control*). Mean level per track was divided by the mean of control and gated at 2× to identify an increase in perturbed cells HES1lo/HES5hi and HES1hi/HES5lo; markers indicate single cells, *n*=4 experiments, 229 tracks. (G) Representative examples of Venus::HES5 and HES1::mScarlet-I fluorescence intensity (normalised to the mean of control) observed in siRNA conditions. Right: comparison of HES1 and HES5 period values in single oscillatory cells compared to hybrid cells. Data points are values from individual cells; bars indicate the median. Two-tailed Mann–Whitney test [*P<0.05; ns, non-significant (*P*=0.2779)].

Our *in vitro* data shows that HES1 and HES5 synchronise and adopt a similar 3-4 h period in co-expressing cells. However, HES1 and HES5 have reported differences in kinetic parameters that can impact on periodicity. Specifically, while the mRNA half-lives of HES1 and HES5 are very similar (between 20 to 30 min), the protein half-life of HES1 is reported to be 22 min, which is considerably shorter than the 80-90 min reported for HES5 (Hirata et al., 2002; Bonev et al., 2012; Manning et al., 2019). To understand how protein stability differences may impact on the period duration, we used a previously developed model of HES1 protein repressing *Hes1* mRNA via a Hill function with a set time delay of 29 min, which produces oscillations of 2- to 3-fold change in amplitude, which is similar to our data (Lewis, 2003; Monk, 2003; Goodfellow et al., 2014). HES1 persistently oscillates for auto-repressive Hill coefficient of n≥5; meanwhile, HES5 can show either damped (n=5 to 6) or persistent (n=7) oscillations (Fig. S4A). This indicates that a high degree of nonlinearity is required for oscillatory dynamics, consistent with parameter inference from live data placing HES1 and HES5 at Hill coefficient values from 5 to 15 (Heron et al., 2007; Burton et al., 2021). Based on experimentally validated HES1 or HES5 mRNA and protein degradation rates (see Materials and Methods), the HES model simulations indicated that the HES1 predicted period was ∼2.5 h, whereas the HES5 predicted period was longer (∼4 h) (Fig. 4A, Fig. S4A,B).

### The periodicities of HES1 and HES5 converge when they are co-expressed

Across the population, the median HES1 period was 3.3 h, similar to HES5 when averaged across cells with different levels of expression (Fig. 2J). Consistent with co-oscillations at different levels, we observed no significant correlation between period and level when analysed as mean per track or per cycle (Fig. S4D,E). To resolve potential heterogeneity, we stratified cells into quartiles based on average HES expression. In the lowest HES5 quartile (0-25%, representing cells with low or no HES5), the HES1 period was 1 h 14 min shorter than in higher-expression groups (Fig. 4B). Similarly, in the lowest HES1 quartile, the HES5 period was shorter by 36 min compared to cells co-expressing both factors (Fig. 4C). Short HES1 periods were also specifically associated with low HES1 levels (<25%, reduced by 1 h 10 min), a relationship not observed for HES5 (Fig. S4C). At the single-cell level, dynamic transitions were observed. In a subset of priNPCs (3/18 cells tracked over 15 h), the HES1 period shifted from short to longer durations as HES5 expression increased within the same cell (Fig. 4D), suggesting possible entrainment between the two oscillators. However, such transitions are likely underestimated, as only 4% of tracks exceeded 15 h, limiting the ability to capture these dynamics. Taken together, these findings suggest that, in NPC cultures, the HES1 oscillator and, to a lesser degree, HES5 can operate outside their predicted dynamic regime and show a common period when they are co-expressed that cannot be fully explained from the current reported values for protein and mRNA stability.

### A coupled HES1-HES5 oscillation model explains synchronisation at a common period

We hypothesised that the HES1 and HES5 period deformations could be due to HES1-HES5 cross-repression causing bidirectional entrainment. To test this hypothesis, we generated a coupled oscillators model that combines the effects of HES1-HES5 cross-repression in addition to self-repression (Fig. 4E, Materials and Methods). In the coupled model, repressive Hill functions are used to encode bidirectional cross-repression, referred to as H15 (HES1 protein repressing *Hes5* mRNA) and H51 (HES5 protein repressing *Hes1* mRNA). Both Hill functions introduce new parameters, including time delays (set to 29 min, the same as the auto-repression time delay) and Hill coefficient, as well as cross-repression thresholds representing the amount of protein required to repress the transcription rate of mRNA of the other gene by 50% (Monk, 2003), namely P15 and P51, corresponding to H15 and H51, respectively. These values act as critical parameters: when one HES protein is below the cross-repression threshold, the other HES protein behaves as ‘free-running’, while cross-repression occurs at protein levels above the threshold, leading to synchronisation across a range of initial conditions and values of P15 and/or P51 (Fig. S5A,B). At equal values of cross-repressive and auto-repressive thresholds, a regime that we refer to as balanced coupling, HES5 entrains HES1, causing an elongation of its period; however, when both repression thresholds P15 and/or P15 are increased by ∼1.5 times compared to the other Hill threshold values, a shortening of HES5 period is observed, indicating that HES1 can also entrain HES5, albeit in a more-restricted parameter space. *In silico* co-expressing HES proteins adopt a common period ranging from 2.5 to 3.5 h, which is consistent with the experimental data (Fig. S5B).

To test the idea of entrainment experimentally, we have used siRNA to knockdown one Hes gene and measure the level and periodicity of the other in mESC-derived NPCs (Fig. 4F, Fig. S5C, see Materials and Methods). This resulted in a 30-60% reduction in mean HES5 protein levels across the cell population, verified by live cell imaging after 48 h (Fig. S5C). The effect on HES1 protein was not detected in NPCs at a fixed timepoint because it was potentially obscured by large fold-change of oscillations compared to HES5; Importantly, the siRNA treatment was effective reducing HES1 protein and mRNA levels by 30-60% in NMP cultures (Fig. S5D). To confirm the knockdown at the single-cell level, we monitored individual cells using time-lapse imaging and identified that mean HES1 and mean HES5 protein levels were reduced compared to control in 24% of siHes1 and 28% of siHes5 NPCs, respectively. Moreover, the knockdown resulted in cells with perturbed protein expression of both (Hes1^low^/HES5^high^ 28.81% and HES1^high^/HES5^low^ 9.68%; Fig. 4F), indicating that changes in the level of expression whereby each protein is upregulated when the other is knocked down were consistent with bi-directional repression, verifying the cross-repressive motif. However, neither periodicity was reduced when the other HES protein was knocked down (Fig. 4G). Therefore, while there is a correlation between co-oscillation and a change in the individual periodicity of each HES, the effect is unlikely to be due to simple or direct entrainment.

### Either HES can dominate at the single-cell level through a change in cross-repression threshold and/or level

**Fig. 5.**
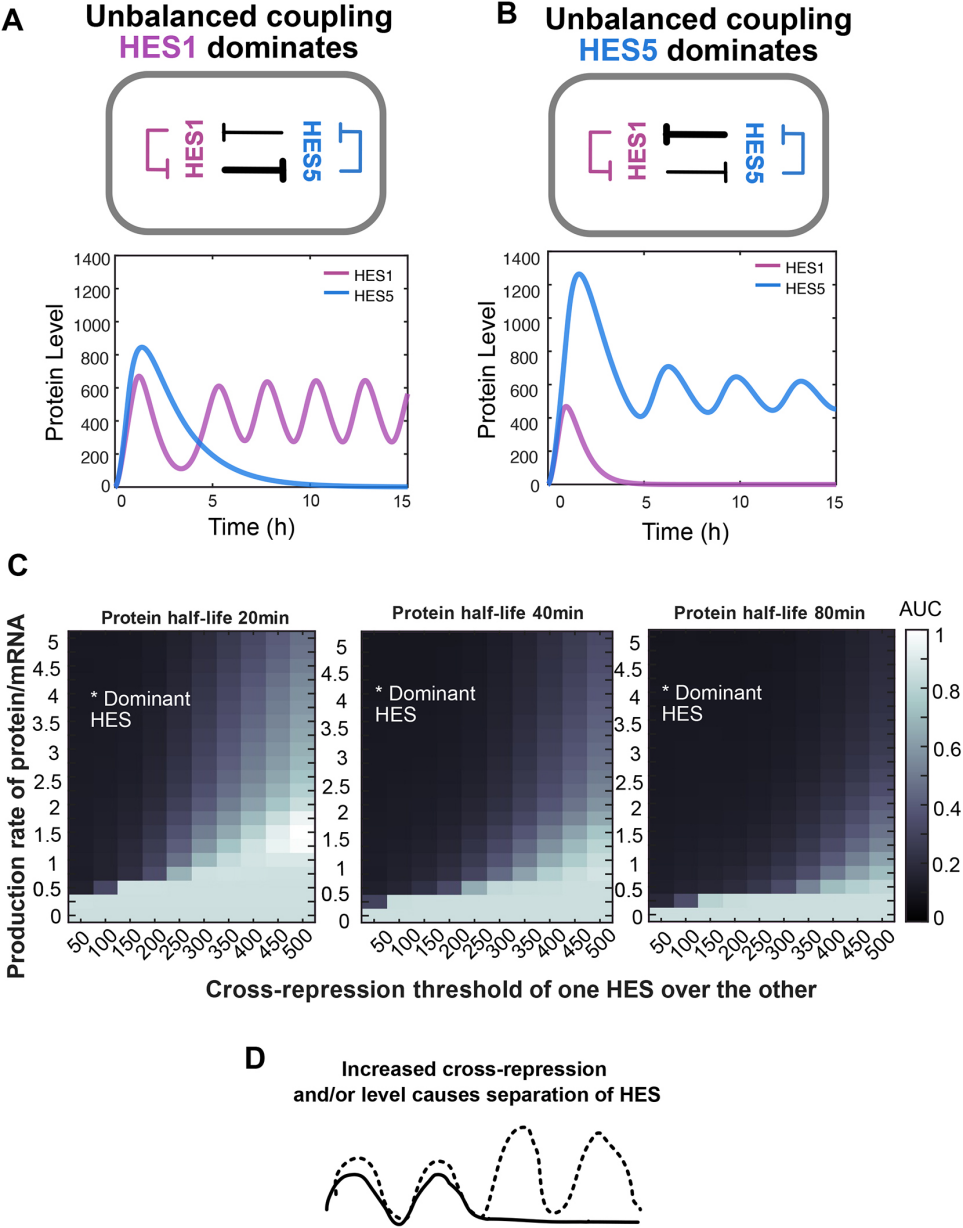
**Mathematical modelling suggests that unbalanced cross-repression between HES1 and HES5 leads to a single dominant HES at the single-cell level.** (A,B) Simulation of coupled HES1 and HES5 dynamics observed at cross-repression values that are unbalanced between HES1 and HES5 at (A) *P*_015_=390; *P*_051_=50 and (B) *P*_015_=50; *P*_051_=390. These dynamic regimes allow one oscillator to suppress the other after a single peak. Following downregulation, HES proteins revert to oscillations of different periods driven by self-repression. Model parameters: 
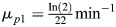
, 
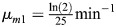
; 
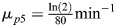
, 
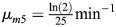
, 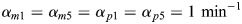; 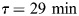; *n*=7; *n*_15_=*n*_51_=5. (C) Mapping the area under the curve (AUC) for HES1 protein levels observed for a range of HES5 production rates (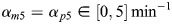), cross-repression threshold values (*P*_051_∈[50, 500]) and protein half-lives (20-80 min). Fixed model parameters: *P*_015_=*P*_01_=*P*_05_=390, 
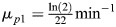
, 
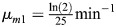
; 
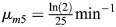
, 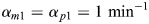; 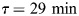; *n*=7; *n*_15_=*n*_51_=5. Dark areas correspond to dominant HES proteins. (D) A representation of HES protein separation.

As in other models of cross-repressing TFs, we also observed dynamic regimes where one HES can dominate over the other, which mimics ‘traditional’ bistable states in which expression of one TF is maintained at a high level while the other is repressed over time (Fig. 5A,B). However, in our motif, the dominant TF exhibits oscillations rather than a stable high level. When either cross-repressive threshold (P15 and, separately, P51) is reduced seven to eight times compared to auto-repressive thresholds, this leads to increased repression from one HES that becomes dominant, a regime referred to as unbalanced coupling (Fig. 5A,B). The effect of a change in cross-repression threshold is bidirectional and incremental, as increased cross-repression from either HES leads to progressively less of the other HES in the same cell through a severe dampening of peaks until only the initial HES is observed (Fig. S6A).

A similar effect can be obtained for cross-repression threshold values that are equal to auto-repression values, in cases when the level of either HES is increased through higher mRNA and protein production rates, leading to incremental suppression of peaks in the other HES, albeit less effective in eliminating low amplitude pulsing compared to cross-repression (Fig. S6A versus S6B). Moreover, a combined strategy (change in cross-repression threshold and production rates) can reduce cumulative HES (area under the curve) at any protein stability, with the cross-expression threshold providing finetuning of cross-repression even at low production rates, i.e. less than 1 (Fig. 5C). Other parameters, such as time delay, did not have an impact (Fig. S6C).

### During *in vitro* differentiation, neural progenitors can exit the oscillatory hybrid state into a single oscillating HES state

**Fig. 6.**
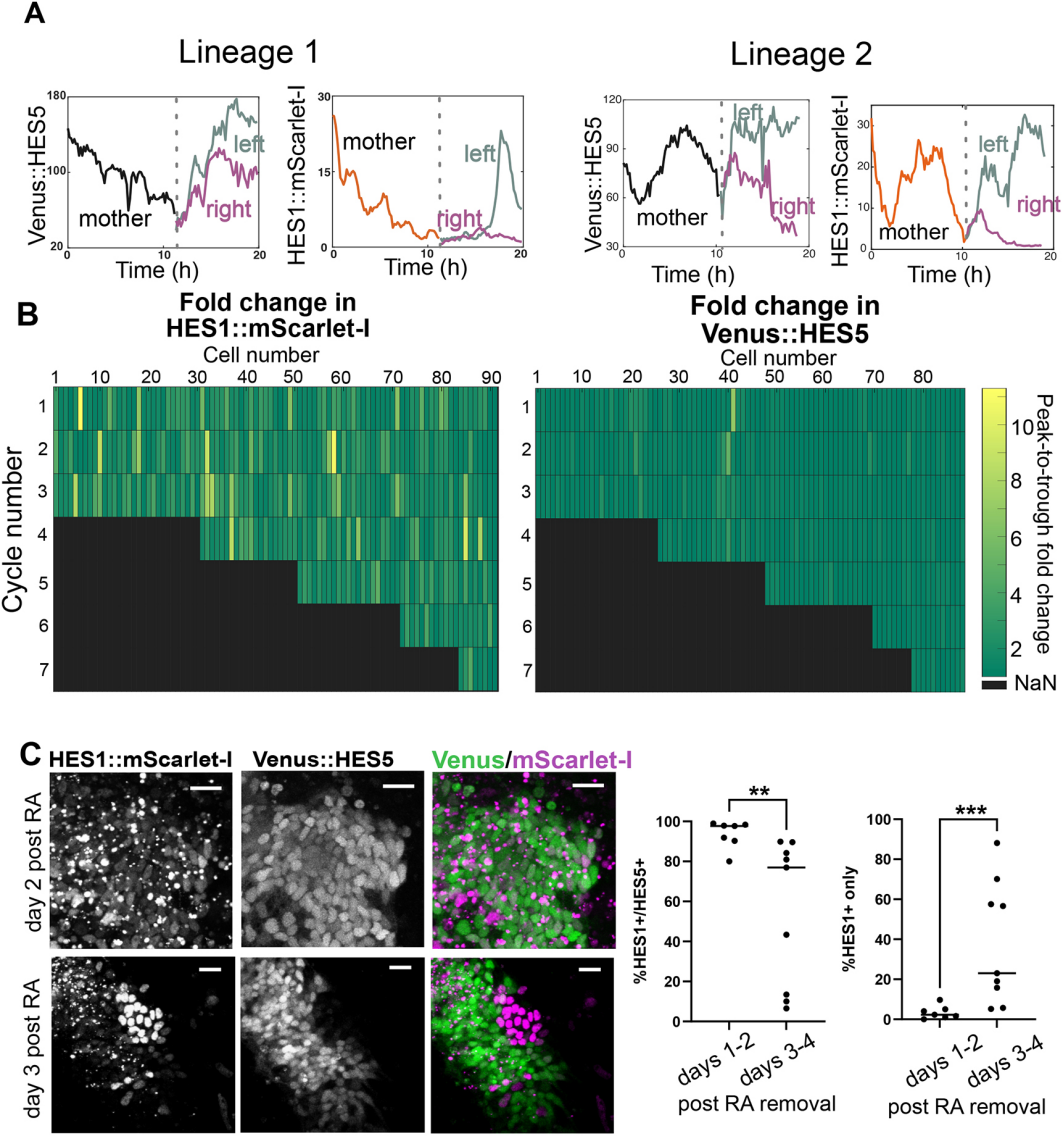
**Further exploration of HES1 and HES5 dynamics in mouse embryonic stem cell-derived neural progenitor cell cultures.** (A) Representative examples of HES1::mScarlet-I and Venus::HES5 dynamics in mother and corresponding daughter cells (left and right). In both lineages, the mother cell and left daughters co-express HES1 and HES5, whereas right daughters transition into HES5 only. (B) Mapping of fold-change (indicative of amplitude) of HES1 and HES5 observed in the same cell over time. Rows indicate cycle number; columns indicate individual cells ordered by cycle length. (C) Representative images and comparison of double-expressing and single-expressing HES1 fractions observed in live neural progenitor cells between 2 and 4 days after retinoic acid (RA) removal. Scale bars: 20 μm. *n*=5 experiments, 1421 cells. Two-tailed Mann–Whitney test (**P<0.01, ***P<0.001). Data points are values from individual images; bars indicate the median.

Consistent with the presence of double HES-positive but also single HES1-positive progenitors (Fig. 1E), as well as model predictions, a transition from a co-oscillating hybrid state into a single oscillatory HES1 state occurs in mESC-derived NPCs as they undergo neural differentiation (Fig. 6). Interestingly, transitions seem to occur following divisions (Fig. 6A); however, only 5/24 divisions have divergent HES patterns, with other examples showing that co-oscillations can persist in both daughter cells (Fig. S7A,B). This shows that exit from the oscillatory hybrid state can occur at different times in progenitors under the same conditions.

To understand if the experimental data are consistent with damped or persistent oscillations during differentiation (both of which are possible in the HES1-HES5 model), we analysed the fold-change in the same track over time. In both cultures, we observed that fold-change values are variable but not progressively reduced over 6-7 consecutive cycles in co-expressing cells and are not correlated to the level of either HES (Fig. 6B, Fig. S7C,D). These observations argue against damped oscillations and in favour of persistent oscillatory states with either hybrid or single HES. When monitored at later stages in the differentiation, the proportion of double HES-expressing NPCs is reducing, with more HES1-only cells being observed over time, suggesting an increase in transitions out of the hybrid state as development progresses, albeit with low probability (Fig. 6C).

The simulated mutually exclusive dynamic regimes, supported by experimental observations, suggest that HES1 and HES5 have the capacity to either co-express (in which case they co-oscillate) or resolve into one dominating over the other while continuing to oscillate. Our model explorations show that exclusivity can occur through a change in cross-repression threshold and/or increased abundance via production rates. In addition, experimental evidence suggests parameters may change in some of the progenitors following cell division.

### The HES1+/HES5+ hybrid state resolves during spinal cord development

**Fig. 7.**
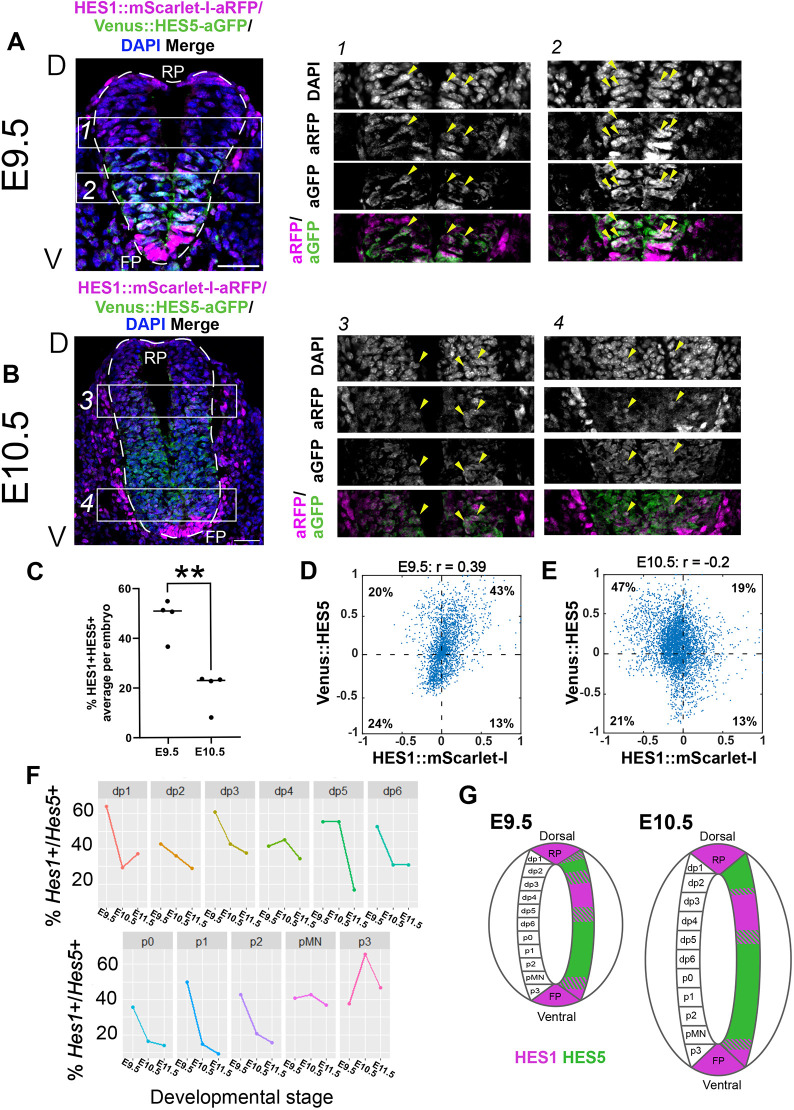
**Spinal cord tissue expression of HES1 and HES5 at different stages in development.** (A,B) Cryosections of mouse embryonic spinal cord at E9.5 (A) and E10.5 (B) in embryos containing endogenous HES1::mScarlet-I and Venus::HES5 imaged at single-cell resolution. Colour channels represent immunofluorescence of nuclear DAPI, HES1::mScarlet-I detected with anti-RFP and Venus::HES5 detected with anti-GFP in the same slice. HES1 is highly expressed at the floor plate (FP) and roof plate (RP), where no HES5 is present. Outlined areas 1-4 contain double-expressing nuclei (arrowheads). (C) Comparison of E9.5 versus E10.5 percentage of nuclei co-expressing HES1::mScarlet-I (from anti-RFP) and Venus::HES5 (from anti-GFP). Nuclei found within the basal boundary were considered, excluding cells in the RP and FP (identified as HES1^+^). Data points are mean values from multiple cryosections of the same embryo; bars indicate the median. Two-tailed unpaired *t*-test (**P<0.01). (D,E) Scatter plot representations of single-cell data collected from E9.5 (D) and E10.5 (E) cryosections in nuclei within the basal boundary, excluding the RP and FP. Intensity values were background subtracted and normalised to maximum per section. Pearson's correlation coefficient is indicated (r); percentages in each quarter correspond to fractions of single-, double- and negative-expressing nuclei observed overall. (F) Co-expression analysis using transcriptomics data from Delile et al. (2019) in different progenitor types in the dorsoventral axis. (G) Spatial localisation of HES1 and HES5 in development.

To understand if the hybrid state exists in NPCs *in vivo* and whether exit from hybrid state occurs during development, we explored the HES expression in domains along the mouse spinal cord dorsoventral axis (Fig. 7, Fig. S8). We mapped *Hes1* and *Hes5* between E9.5 and E11.5 using existing single cell transcriptomics data from mouse embryonic spinal cord (Delile et al., 2019); both Hes genes are widely found in spinal cord progenitors with a tendency for *Hes5* to become more abundant over development and *Hes1* to become more restricted to areas where *Hes5* is low (Fig. S8A). Expression patterns at E9.5 were least segregated, and establishment of separate Hes-expressing domains started from E10.5 onwards.

We used cryosections of dual HES1::Scarlet-I/Venus::HES5 embryos (amplified with anti-RFP and anti-GFP antibodies) to compare protein co-expression at E9.5 and E10.5 (Fig. 7A,B, see Materials and Methods). HES1 expression was highest at the floor plate and also present at the roof plate where no HES5 is observed (Fig. 7A,B, left-most panel). Outside the floor plate and roof plate regions, at E9.5, HES5 showed a prominent ventral expression domain, with HES1 expression overlapping both the dorsal and ventral sides of the domain, indicating co-expression of HES1 and HES5 in the dorsoventral axis (Fig. 7C). At E10.5, the HES1 dorsal and HES5 ventral expression domains became larger, comprising more nuclei; areas of co-expression of HES1 and HES5 became further restricted to the edges of the HES5 ventral domain (Fig. 7B). Overall, the percentage of double-positive nuclei reduced from 50% to only 23% between E9.5 and E10.5 (Fig. 7C, Fig. S8B,C). Intensity mapping in the same cells showed that, at E10.5, there is an increase in the HES5-only fraction at the expense of hybrid cells when compared to E9.5, indicating transitioning progenitors (Fig. 7D,E). Similar to our *in vitro* NPCs, where we observed both persistence and exit in different progenitors, the rate of transition out of the hybrid *Hes1+*/*Hes5+* state was variable in different progenitors over developmental time (Fig. 7F). Our analysis of protein (summarised in Fig. 7G) complemented by existing spinal cord data (Kicheva et al., 2014; Sagner et al., 2018; Delile et al., 2019) suggests the presence of a spatio-temporal mechanism that modulates the time of exit from the hybrid state potentially impacting the differentiation rate (Kicheva et al., 2014).

**Fig. 8.**
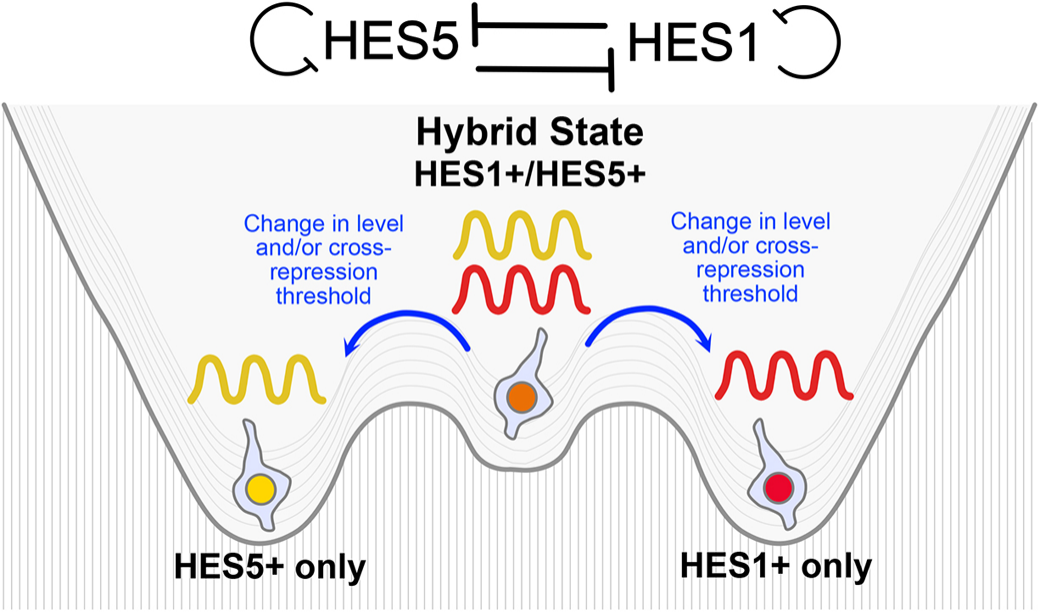
**Graphical representation of the HES1-HES5 dynamics**. The HES1 and HES5 proteins are co-expressed and co-oscillate in early development forming a hybrid state; co-expression resolves into single oscillatory HES protein states through a change in level and/or cross-repression threshold.

Taken together, our *in vitro*, tissue and theoretical observations suggest that cross-repressing TFs HES1 and HES5 are co-expressed at the single-cell level in early spinal cord development, forming a hybrid state where they oscillate in-phase, each converging to a common (or shared) periodicity. However, at later stage, one HES dominates over the other through cross-repression and most progenitors adopt one or the other oscillatory HES. In our model, this effect can be explained through a reduction in the thresholds of cross-repression and/or an increase in the level of one HES, see graphical representation in Fig. 8.

## DISCUSSION

HES1 and HES5 are two transcriptional repressors that can form a cross-repressive toggle switch motif and have mutually exclusive expression domains along the dorsoventral axis of the developing spinal cord. Using cell cultures and live-imaging reporters, we have shown here that HES1 and HES5 co-oscillate in primary NPCs and in NPCs derived from mESCs. These co-oscillating HES1/HES5 cells represent a hybrid progenitor state from which cells can transition into another progenitor state of a single dominant oscillatory HES. Expression analysis in the developing spinal cord showed that HES1/HES5 co-expressing cells exist *in vivo* but more cells co-expressed HES1 and HES5 at stage E9.5 than at stage E10.5. This indicates that a transition from the hybrid to a dominant HES state occurs in spinal cord over development.

Cross-repressive TF motifs are well known for creating bistable (toggle) switches that resolve in one of two stable states where only one or other TF is expressed. In such a classic bistable motif, a hybrid state where the TFs co-exist is very unstable and transitory. However, in our system, the hybrid state appeared stable because cells in culture oscillate for many hours and/or days, without damping of oscillations; unlike the traditional bistable motif, the HES1/HES5 cross-repressive motif resolves to two oscillatory states where only one or the other HES proteins continues to oscillate. Thus, our work expands our understanding of transitions in development as our data support the existence of more attractors than currently appreciated. Our study suggests that oscillatory dynamics of cross-repressing (and auto-repressing) TFs permit the existence of a stable hybrid state that would otherwise be expected to be transient and experimentally inaccessible. The resolution of the HES1-HES5 motif involves two additional attractors in neural progenitors, each representing the single oscillatory HES, before it transitions further into a state where HES expression is extinguished as cells differentiate (Manning et al., 2019).

Using computation, we demonstrate that both oscillatory hybrid and dominant states can be explained by cross-repression between HES1 and HES5, coupled with their individual capability to oscillate. While HES1 and HES5 oscillate by a combination of auto-repression, protein and mRNA instability, and appropriate time delays, other motifs, such as non-autorepressive TFs (Bokes and King, 2019; Guantes and Poyatos, 2008) or the AC/DC motif of 3TFs, are also known to generate hybrid oscillatory states *in silico* (Perez-Carrasco et al., 2018). Therefore, we expect our findings to be more widely applicable. *In silico*, we found that the resolution of cross-repressing and oscillatory TFs takes place when cross-repression in either direction becomes stronger than the other, and stronger than autorepression, or when production of either TF is increased, or indeed a combination of the two strategies. *In vivo*, such resolution could be mediated by region-specific negative regulation of HES by TFs such as OLIG2 and HES6 during spinal cord neurogenesis (Sagner et al., 2018; Fior and Henrique, 2005). It may also involve additional molecular mechanisms proposed for somitogenesis, such as modulation of degradation rates and strength of DNA-binding activity through Hes/Her dimerization (Schröter et al., 2012), as well as increasing gradients of transcriptional delay in Her1 (Ay et al., 2014). Additionally, the model is deterministic, while the data are stochastic and, as such, we cannot exclude the possibility that stochasticity may also contribute to resolution into single oscillatory states. Although our study focuses on the intrinsic dynamics of the HES1-HES5 network, *in vivo* this circuit is embedded within a broader regulatory context. Morphogen signalling pathways, additional bHLH transcription factors (e.g. OLIG2) and chromatin or epigenetic regulation may influence the dynamics of the network, potentially modulating oscillatory properties.

Resolving gene expression through a hybrid, double-positive oscillatory state, rather than a bistable switch, offers several advantages. One key benefit is the ability to extend the decision-making window. In some progenitor cells, this hybrid oscillatory state may delay the final resolution of the switch, allowing expression domains to be gradually established and refined over time, in sync with the timeline of neurogenesis. This finding complements previous findings where boundary areas of mutually exclusive and opposing TFs (e.g. A and B) was shown to contain a mix of cells with A and B expression, which are gradually resolved to either A or B across the border, as a property of the network (Exelby et al., 2021). Our findings suggest that such domain borders are also sharpened by the resolution of cells that initially have mixed (oscillatory) A/B characteristics to either (oscillatory) A or B. Additionally, this mechanism provides a form of functional compensation; under certain parameter conditions, the non-dominant HES can continue to oscillate at low amplitude, remaining poised for activation. If repression is lifted, for example, through deletion of the dominant factor, this latent activity could allow rapid upregulation across the spinal cord, supporting robustness in patterning (Hatakeyama et al., 2004).

Our study shows that, in addition to inter-cellular coupling of oscillating genes (Niwa et al., 2007; Biga et al., 2021; Hawley et al., 2022, 2025), intra-cellular coupling can also take place, in this case, leading to in-phase synchronisation of two oscillators. *In silico*, we showed that the free-running period of HES1 is shorter than the free-running period of HES5, as indeed is predicted by their differential protein stability. Furthermore, when co-expressed, in-phase oscillations of HES1 and HES5 with a common period are observed. Our computational modelling suggests a mutual entrainment event that results in these two HES proteins acquiring common periodicity and phase when co-expressed. This finding extends our understanding of frequency deformations beyond systems of identical oscillators (Rohm et al., 2018) and highlights the versatility of coupled oscillators, in this case enabling each HES to oscillate outside their normal frequency, a behaviour suggesting nonlinearity close to an infinite period bifurcation, as proposed in the somitogenesis clock (Sanchez et al., 2022). The mutual entrainment is also consistent with the tissue culture data where each HES has a different periodicity from their common period when the level of the other HES is very low, in order of 0-25%. However, our individual HES knockdown data did not reveal alterations in the periodicity of the other HES, arguing against an entrainment event. It could be that they are both independently responding to a third cue that is present only in co-expressing cells but it is also technically possible that the level of knockdown did not reach 0-25% in sufficient cells. More-sophisticated methods of knocking-down will be needed before the question of entrainment is unequivocally answered.

Nevertheless, it is possible that the in-phase aspect of co-expression serves to preserve the HES decoding properties by repressing downstream targets in the ‘high expression’ phase while allowing a probability for a transition to occur in the ‘low expression’ phase (Soto et al., 2020). Indeed, in a different context, the phase divergence (or temporal redundancy) of TF paralogs with overlapping targets can be modulated from out-of-phase to in-phase to upregulate the level of target genes during the yeast stress response (Wu et al., 2021).

In conclusion, oscillatory expression enables a HES1 and HES5 hybrid state that resolves into a single HES (Fig. 8), leading to spatial segregation of HES1 and HES5 in spinal cord. In addition, coupling between these two oscillators, and the resulting synchrony when they are expressed in the same cells, may preserve and even enhance the decoding functionality of HES oscillators.

### Limitations

The majority of our work used *in vitro* cultures and we have not confirmed the presence of co-oscillations, their resolution or, indeed, differences in dynamics between HES1 and HES5 *in vivo*. Our HES1-HES5 model is deterministic and not fully quantitative, and, as such, levels are not calibrated to abundance, and slow-varying fluctuations in level over time are not accounted for. A full exploration of attractor states in a quantitative model would strengthen the predictions on cell-fate decisions. Finally, our interpretation that oscillations enable a hybrid state are based on a single pair of oscillatory and cross-repressing TFs that oscillate in phase. Validation with other cross-repressing TFs would strengthen this generalisation.

## MATERIALS AND METHODS

### Mouse lines

Animal experiments were performed within the conditions of the Animal (Scientific Procedures) Act 1986. *Venus::Hes5* knock-in mice [ICR.Cg-Hes5<tm1(venus)Imayo>] (Imayoshi et al., 2013) were obtained from the Riken Biological Resource Centre, Japan. *Hes1::mScarlet-I* knock-in mice were generated by Marinopoulou et al. (2021). The double knock-in *Venus::Hes5*/*Hes1::mScarlet-I* mice are viable with mendelian ratios*.* We used the EASI-CRISPR strategy (Quadros et al., 2017) to generate a C-terminally tagged *Hes1::mVenus* mouse line using the same protocol (Marinopoulou et al., 2021; Bennett et al., 2021). To generate the long single-strand DNA donor repair template, a homology flanked flexible linker-3xFLAG-mVenus DNA sequence was cloned and used as a template in an initial PCR reaction with the following primers: Hes1_lssDNA_F, catgctcccggccgcCATGGGAATTCGGTACcaacagtgggacctcggt; and Hes1_lssDNA_R, caagttcgtttttagtgtccgtcagaagagagaggtgggctagggactttacgggtagcagtggcctgaggctctcacttgtacagctcgtccatgcc. Potential founder mice were screened by PCR, using primers that flank the homology arms (Geno F, ttgcctttctcatccccaac; Geno R, gcagtgcatggtcagtcac), used in combination with internal mVenus primers (mVenus F, CACATGAAGCAGCACGACTT; mVenus R, TCCTTGAAGTCGATGCCCTT). Germline transmission was confirmed through PCR and sequencing, and a colony was established. For timed matings, E0.5 was considered as midday on the day a plug was detected.

### Establishment and maintenance of primary neural progenitor cultures

Primary neural progenitor cells were isolated from dissected spinal cords of E10.5 embryos obtained from crossing *Venus::Hes5* and *Hes1::mScarlet-I* knock-in mice or dissecting LGEs of E13.5 embryos from crossing the *Hes1::mVenus* with *Hes1::mScarlet-I* knock-in mice. All lines were genotyped to confirm the presence of knock-in fusion alleles, mScarlet-I (Marinopoulou et al., 2021) and mVenus genotyping primers (Venus R, CTACTTGTACAGCTCGTCCATGCC; Venus F, GTGTCTAAGGGCGAAGAGCTG; mVenus as listed above), to identify cells expressing both fluorophores. Primary lines were maintained using protocols from Pollard (2013). Dissociated cells were cultured on laminin-coated plates in DMEM/F-12 media containing 4.5 mg/ml glucose, 1× MEM non-essential amino acids, 120 μg/ml bovine albumin fraction V, 55 μM 2-mercaptoethanol, 1× GlutaMAX, 0.5× B27, 0.5× N2 supplemented with 10 ng/ml EGF and 10 ng/ml bFGF. Cell lines were passaged routinely every 2-3 days by dissociation with Accutase and re-seeded at split ratios between 1:2 to 1:4. For imaging experiments, cells were seeded at 12,000 to 23,000 cells/cm^2^ in a Cell view 35 mm glass-bottomed dish and allowed to proliferate for 2-3 days. As expected, priNPC cultures express the progenitor marker SOX2 (Graham et al., 2003) (Fig. S1A). A list of reagents is included in Table S1.

### Establishment and maintenance of the *Venus::Hes5/Hes1::mScarlet-I* mouse ESC lines

All incubation steps were carried out at 37°C under 5% CO_2_. ESC derivations were performed as described by Nichols et al. (2009) with small alterations: Embryos were flushed from oviducts at the 4- to 8-cell stage (E2.5) and placed into KSOM plus 2i (1 μM PDO325901 and 3 μM CHIR99021) with PBS into the outer well to avoid evaporation, and incubated for 2 days. If the embryos had not hatched, the zona pellucida was removed using Acid Tyrode's solution and the trophectoderm was removed by incubating the embryos for 1 h in KSOM+20% rabbit anti-mouse antiserum, rinsing three time in pre-equilibrated N2B27 media (comprising a 1:1 mix of Neurobasal and DMEM/F12 plus 1× Glutamax, 0.5× N2, 0.5× B27, 0.5% BSA and 0.1 mM β-mercaptoethanol) and then incubating for 30 min in KSOM+20% guinea pig serum. The trophectoderm lysate was then manually removed using a finely drawn pipette. The isolated ICM cells were placed into separate wells of a 96-well plate (pre-coated with 20 μg/ml laminin 511+1 mM fibronectin in N2B27+2i+10 ng/ml LIF, and incubated for ∼7 days, until there is a large outgrowth). The outgrowths were passaged using Accutase and gradually expanded until the lines were established, and at which point they were maintained on 0.2% gelatin-coated plates in N2B27+2i+LIF (Ying et al., 2008) or in ESGRO+2i LIF. A list of reagents is included in Table S1.

### Differentiation of mouse ESCs to dorsal neural progenitors

We used a previously described protocol that enriches for dorsal interneurons 4 to 6 (Gupta et al., 2022). The differentiation media consisted of 1:1 DMEM/F-12 and Neurobasal supplemented with 0.5xN2, 1xB27, 2 mM Glutamax, 0.1 mM 2-mercaptoethanol and 1%BSA. Between 50,000 to 100,000 mESCs per cm^2^ were seeded on Matrigel coated culture Cell view 35 mm glass-bottomed dish with 4 partitions in differentiation media containing 10 ng/ml bFGF (day1) followed by 10 ng/ml bFGF and 3 μM CHIR99021(day2) to generate neuromesodermal precursors (Gouti et al., 2014). Neural induction was carried out with 100 nM retinoic acid (RA) (days 3-4). From day 5 onwards cells were maintained in differentiation media (no RA) and Venus::HES5 expression was observed immediately after RA removal, see diagram in Fig. S1B. A list of reagents is included in Table S1.

### Timelapse imaging and single cell tracking

Movies were acquired using Zeiss LSM880 microscope and GaAsP detectors using a Fluar 40× objective. HES1-mScarlet and HES5-Venus were detected using a 561 nm and either 488 nm or 514 nm laser (Venus). *Z*-sections covering the depth of the cells were acquired every 10 min. Primary neural stem cells were tracked using maximum intensity projections images created with Fiji. Single-cell tracks were produced in Imaris using the ‘Spots’ and ‘Track over time’ function with the Brownian motion algorithm. All tracks were manually curated to ensure accurate single-cell tracking, and generated mean intensity over time, representing protein concentration over time in single cells. The 2D spot size was set to 2.5 μm diameter in *x* and *y* dimensions. Tracking of mESC-derived neural progenitors was performed in 3D images to account for the fact that individual cells can overlap in the *z*-plane. The 3D spot size used was 3 μm in diameter in *x*, *y* and *z* dimensions. A list of reagents is included in Table S1.

### Immunofluorescence of cryosections

Mouse embryo trunks from E9.5 and E10.5 embryos were fixed in 4% PFA for 1 h at 4°C, followed by three quick washes with 1×PBS and one longer wash for 1 h at 4°C. Embryos were equilibrated overnight in 30% sucrose (MERCK) at 4°C before mounting in Tissue-Tek OCT (Sakura) in cryomoulds and freezing at −80°C. 12 μm sections were cut on a Leica CM3050S cryostat and collected using Superfrost Plus adhesion microscope slides. For immunofluorescence, sections were washed twice with PBS and stained using the same protocol as for cultured cells. Cells were fixed using 4% PFA for 15 min followed by a PBS wash. Permeabilisation and blocking was carried out using PBS+0.1% Triton X-100+10% donkey serum for 1 h at room temperature. Primary antibodies (see Table S1) were diluted in PBS+0.1% Triton X-100+1% donkey serum and incubated on the samples overnight at 4°C. Three 10 min washes in PBS were then performed and then secondary antibodies (see Table S1) added, diluted in PBS+0.1% Triton X-100+1% donkey serum, incubated at room temperature for 4 h (for tissue sections), kept in the dark or left at 4°C overnight (for cultured cells). Nuclei were stained with DAPI or Hoechst 33342. Three more PBS washes were then performed, and the cells were left in PBS for imaging or tissue sections were mounted with Gold Antifade Mountant. For tissue sections, anti-RFP antibody was used to detect Hes1::mScarlet-I endogenous knock-in, and three GFP antibodies were used to detect Venus::HES5, all with similar results. A list of reagents and antibodies is included in Table S1.

### Knockdown by siRNA

siRNA-mediated knockdown of HES1 and HES5 were performed using ON-TARGETplus SMARTpool siRNAs (Horizon Discovery). A non-targeting siRNA pool was used as a control. Mouse ESCs were seeded as normal and differentiated to specific stages, i.e. NMP and NPC (Fig. S1B). Transfection was carried out using Lipofectamine RNAiMAX (Thermo Fisher Scientific) in OptiMEM (Gibco) reduced serum media at 20 and 40 nM siRNA concentrations. Knockdown efficiency in NPC and NMP cells was assessed after 48 h post-transfection by live imaging of Venus::HES5 and HES1::mScarlet-I fluorescent intensity. Efficiency of siRNA at NMP stage was verified by qPCR using primers against *Hes1* (forward, GGC-GAA-GGG-CAA-GAA-TAA-ATG; reverse, GTG-CTT-CAC-ATG-CAT-TTC-CAG) (Kong et al., 2015). Overall knockdown efficiency was estimated at 30-60% (Fig. S5C,D). For the timelapse data, we computed the mean level of HES1 and HES5 in the same cell over time and normalised the expression by dividing by the mean of HES1 and HES5 in the control cells monitored in the same experiment (Fig. 4F,G).

### Quantification of positive cells

Endogenous levels of HES1-mScarlet-I and HES5-Venus were quantified as mean intensity per nucleus using the Imaris ‘Spots’ function applied to the DAPI/Hoechst nuclear marker channel. Negative mean intensity data was generated from cell cultures not containing Venus or mScarlet-I, which were processed in the same way. Mean intensities above the 95th percentile of negative control values were considered positive for HES1::mScarlet-I and Venus::HES5.

Immunofluorescence data from cell cultures and tissue was quantified in the same way, i.e. mean intensity per nuclear spot (detected from DAPI/Hoechst). In cultures, the 95th percentile of values observed secondary only control samples was used as a threshold for positive cells. In tissue, positive nuclei values were considered above the 95th percentile of mean intensities of GFP^−^ and/or RFP^−^ nuclei located outside the neural tube per slice. The criterion used for tissue is more stringent compared to a secondary only control (Fig. S8A,B).

### Detection of oscillations by a Gaussian Process method

We used the statistical approach developed by Phillips et al. (2017) and Manning et al. (2019) to analyse periodicity in single-cell timeseries. Briefly, data were detrended using a Gaussian Process covariance function calibrated to fit slow-varying fluctuations above 2.5× the period of oscillations (detrending parameter 7.5 h). The detrending removes fluctuations in the long-term trend, such as downregulation, and recovers the oscillatory signal with zero mean. We used maximum-likelihood estimation to fit the detrended data timeseries with two competing models: a fluctuating aperiodic model (null) and a periodic model (alternative). We used the log-likelihood ratio (LLR) statistic to compare the likelihood of data being oscillatory or non-oscillatory, and determined the oscillators based on a false discovery rate of 5% independently per experiment.

### Quantification of period from timeseries data

As described by Phillips et al. (2017) and Manning et al. (2019), we used maximum likelihood to determine the best fitting period value for a Gaussian Process covariance model that can reproduce the data. This period value is characteristic of the entire timeseries and, as such, it does not capture variations in period over time. To account for changes over time, we used the Hilbert transform to identify the start and end of each oscillatory cycle, and determined the instantaneous period (and level), as shown in Fig. S4E.

### Peak to trough fold-change from timeseries data

We used the routines described by Manning et al. (2019) to identify peaks and troughs in the timeseries. Peaks were paired with subsequent troughs, and we computed peak to trough fold-change as the ratio of raw intensity at the peak divided by intensity at the trough per track. We reported maximum peak to trough fold-change throughout the manuscript except for Fig. S7D, which shows instantaneous fold change.

### Phase-phase mapping

The Hilbert transform was used to reconstruct the phase angle in the HES1 and HES5 timeseries. Phase-phase mappings were generated by plotting the phase of HES1 against the phase of HES5 in the same cell at any time. We used dscatter.m (Eilers and Goeman, 2004) to colour code the density of datapoints.

### Detection of in-phase dynamics from timeseries

We used two approaches to determine how many co-expressing timeseries are in phase. Firstly, using HES1-HES5 phase-phase maps in the same cell, we computed the ratio of in-phase versus out-of-phase observations, a measure of synchrony described by Biga et al. (2021). Timeseries with a ratio higher than average values observed by chance (computed by cross-pairing HES1-HES5 in different cells from the same experiment) were classified as in phase. Secondly, we used cross-correlation analysis to measure the phase shift between HES1 and HES5 in the same cell. Using this method, timeseries with a phase shift of less than 30 min were classified as in phase. The two methods provided very good correlation (Fig. S3E).

### Detection of transitions from trend variation

To determine if individual cells showed a transition in the level of HES proteins, we quantified the variability of the trend and compared it between mother and daughter cells. Trends were determined as described in the ‘Detection of oscillations by a Gaussian Process method’ section and used to measure the coefficient of variation (COV, standard deviation over the mean) in either HES. Cells with a COV greater than 0.7 were classified as transitioning (see Fig. S7B).

### Mathematical modelling

We used the deterministic model of HES mRNA-protein regulation described by Lewis (2003) and Monk (2003) to model the variation of protein (*P*) and mRNA (*m*) over time, dependent on production, degradation rates and delayed self-repression:

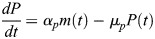


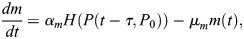
where *α*_*p*_, *α*_*m*_ represent translation and transcription (in absence of protein repression) rates, and *μ*_*p*_, *μ*_*m*_ represent degradation rates for protein and mRNA, respectively. The repression of protein onto mRNA is modelled as a repressive Hill function:

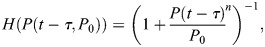
where *τ* represents time delay, *P*_0_ represents repression threshold and *n* is the Hill coefficient.

We selected values for the parameters that generate persistent oscillations of HES, consistent with previous reports (Goodfellow et al., 2014; Lewis, 2003; Monk, 2003). Values of *α*_*p*_, *α*_*m*_ were set to 1 min^−1^ (Goodfellow et al., 2014). The auto-repressive time delay *τ* was set to 29 min (Lewis, 2003); repression threshold *P*_0_ was set to 390, a value identified to oscillate by Goodfellow et al. (2014); the Hill coefficient *n*≥5, a value that yields 2× to 3× peak to trough amplitude of oscillations (Monk, 2003), similar to what we have observed in the data.

In the model, we estimated the period (measured as average peak-to-peak interval) that emerges at different stabilities of mRNA and protein by taking into account that degradation rates are related to half-life, 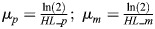, where *HL*_*p*_, *HL*_*m*_ represent protein and mRNA half-life, respectively. In Fig. S4B we can observe that the predicted period for HES1 is shorter than for HES5 primarily due to large differences in reported protein half-life [22 min for HES1 (Hirata et al., 2002); 80-90 min for HES5 (Manning et al., 2019)] and to some extent also due to small differences in mRNA half-life [25 min for Hes1 (Bonev et al., 2012); 30 min for Hes5 (Manning et al., 2019)]. At the reported stability values, HES1 and HES5 have distinct periods (Fig. 4A).

We then generated a coupled model of HES1 and HES5, including delayed auto-repression as well as delayed cross-repression:

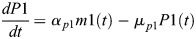


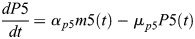


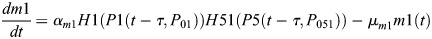


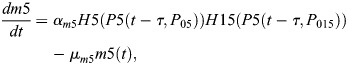
where *P*1, *m*1 and *P*5, *m*5 correspond to HES1 and HES5 protein and mRNA, respectively. In addition to parameters found in the single HES model, the coupled version contains *H*1, *H*5 auto-repressive Hill functions for HES1 and HES5, as well as cross-repressive Hill functions *H*15, representing repression from the HES1 protein onto Hes5 mRNA and *H*51, representing repression from the HES5 protein onto Hes1 mRNA. We used a time delay of 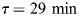 for all Hill functions. Each Hill function has characteristic parameters: auto-repressive Hill, Hill coefficient *n*=7; auto-repressive thresholds, *P*_01_, *P*_05_=390; cross-repressive Hill, Hill coefficients *n*_15_=*n*_51_=5; cross-repressive thresholds, *P*_015,_*P*_051_ (these were varied from 390 to 50 in different simulations; e.g. Fig. S6A). Repression thresholds are inversely proportional to repression strength, such that a low value indicates higher repression compared to a high value.

Other parameters were set in a similar way to the single HES model. Translation and transcription rates in absence of protein repression were set to 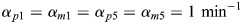 and identical to the single HES model. The protein degradation rates were set to 
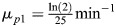
 and 
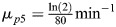
, corresponding to 25 min and 80 min half-life values for HES1 and HES5 protein, respectively. As the stability of mRNA is generally low for both HES proteins (Bonev et al., 2012; Manning et al., 2019), the mRNA degradation rates were set to 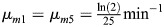, corresponding to 25 min half-life for either HES, resulting in HES5 periods similar to those observed in the data.

### Statistical analysis

Statistical analysis including normality testing was performed using Prism 10.3.0. Detection of periodicity, peak to trough fold change and phase angle was carried out using custom Matlab R2025b routines described by Phillips et al. (2017), Manning et al. (2019), Marinopoulou et al. (2021) and Biga et al. (2021).

### Bioinformatic analysis

sc-RNAseq data from Delile et al. (2019) were analysed in R using Seurat v5 (Hao et al., 2024). Cells were excluded if they had more than 6% UMI counts associated with mitochondrial genes and expressed fewer than 800 genes. Only progenitor cells (annotated in Delile et al., 2019) and E9.5, E10.5 and E11.5 time points were used for the analysis. Co-expression of *Hes1* and *Hes5* was assigned to cells if they had at least 1 count for both genes.

### AI

ChatGPT was used to shorten the Results sections of the manuscript as well as to generate a draft summary statement that was later edited by the authors to fit the study better. The authors subsequently reviewed and edited the content as necessary and take full responsibility for the publication's final content.

## Supplementary Material



10.1242/develop.204969_sup1 Supplementary information
