# Supplementary material for: Oscillatory co-expression of HES1 and HES5 enables a hybrid state in a cross-repressive transcription factor regulatory motif
Source: Development. 2026 Jun 10;153(11):dev204969. doi: 10.1242/dev.204969 (PMC13286377; doi:10.1242/dev.204969)
Supplement: Supplementary information [file develop-153-204969-s1.pdf]

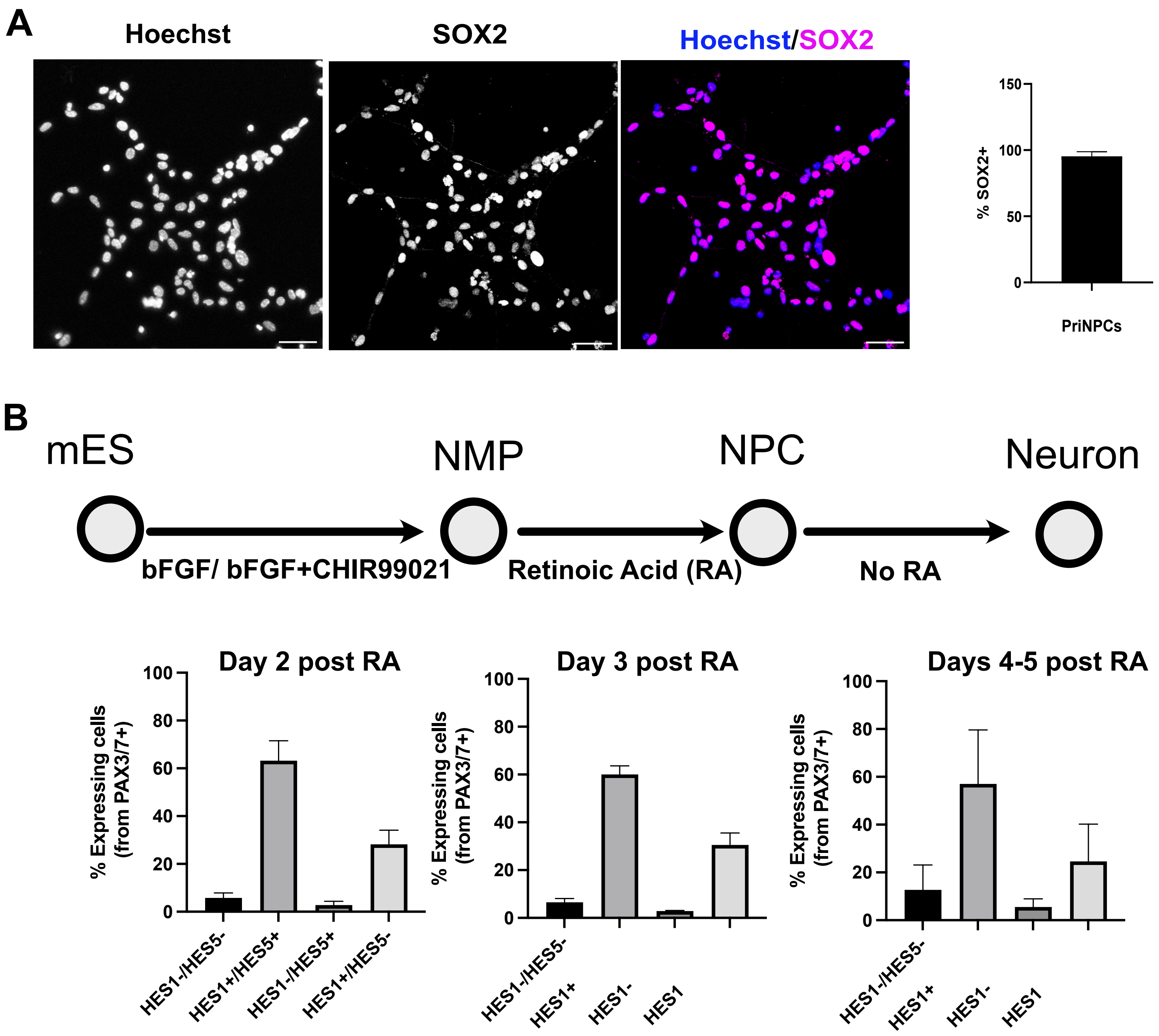

Fig. S1. In vitro protocols to study neural progenitor cells. Related to Fig. 1.

(A) SOX2 expression in primary neural progenitor cells detected by immunofluorescence; 40 x objective, scale bar is 40um; (right-most panel) percentage of SOX2+ nuclei as mean and SD from 3 independent experiments with a total of 1,075 nuclei analysed.

(B) Diagram of the mES differentiation protocol used to generate neuromesodermal precursors (NMP), neural progenitor cells (NPC) and neurons. We focused on the NPCs identified with the progenitor marker PAX3/7 and quantified the percentages of double and single expressing cells between 2 to 5 days post retinoic acid (RA) removal; Day 2: n=3 experiments, 1,628 cells; Day 3: n=2 experiments, 809 cells; Days 4-5: n=2 experiments, 805 cells.

Development • Supplementary information

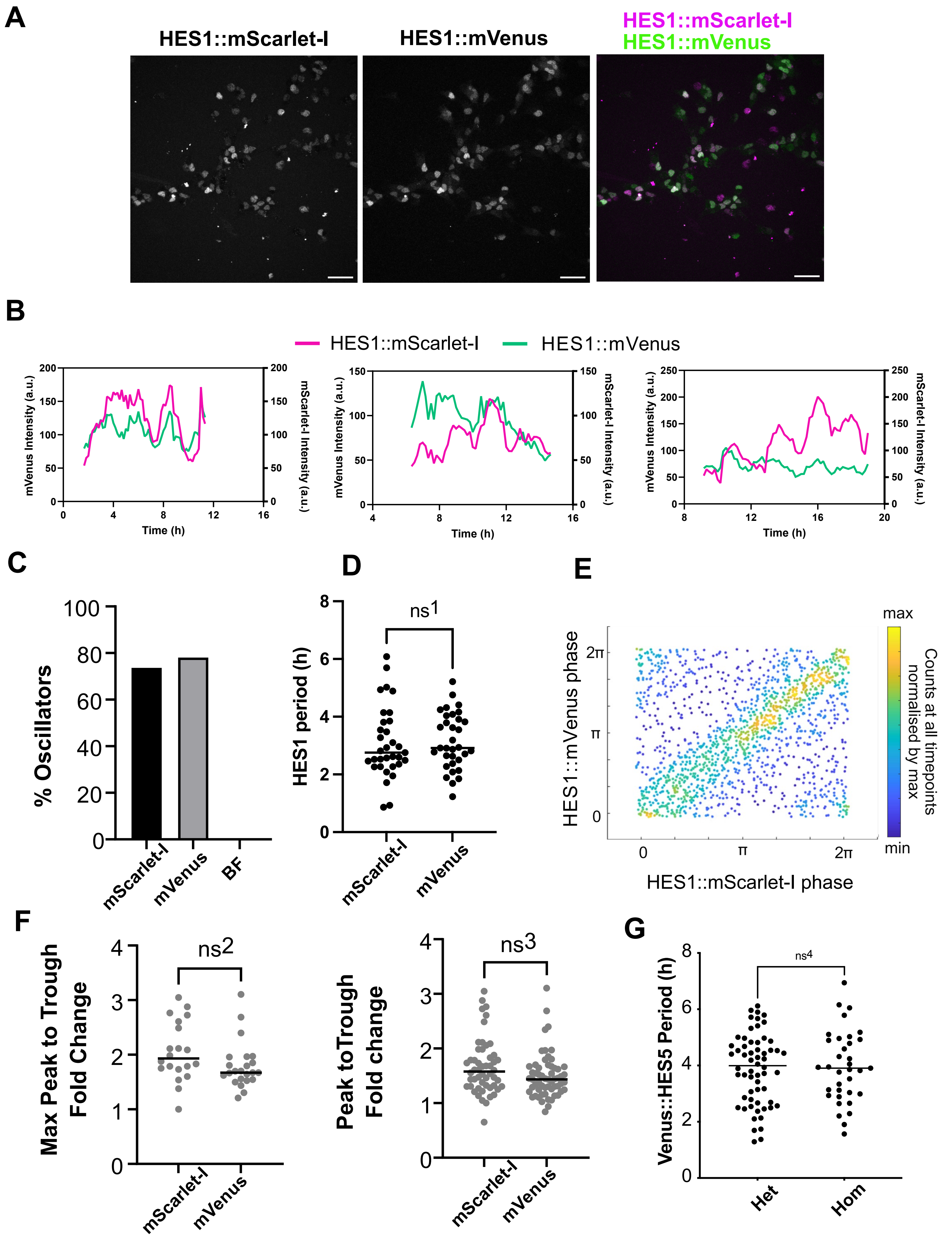

Development • Supplementary information

Fig. S2. Oscillatory dynamics of HES1 and HES5 monitored in one or both alleles. Related to Fig. 2. **(A)** Primary neural progenitor cell cultures obtained from LGE (Materials and methods) containing a dual knock-in for HES1::mScarlet-I and HES1::mVenus to monitor expression in each allele; 40x objective, scale bar 40um.

(B) Representative examples of HES1::mScarletI and HES1::mVenus oscillatory intensity variations observed in the same nucleus over time.

(C) Percentage of oscillatory cells as detected from mScarlet-I or mVenus versus aperiodic variations in the bright field (BF) signal in the same nucleus; n=2 with 33 tracks analysed.

(D) Comparison of HES1 period values observed in mScarlet-I or mVenus; markers indicate cells, line indicates median, unpaired t test, 2 tailed, ns^1=0.8189.

(E) Phase-phase density mapping of detrended HES1::mScarlet-I and HES1::mVenus in the same cell over time indicating synchronous oscillations; markers are color-coded to indicate low and high probability density areas.

(F) Comparison of maximum peak to trough (left) and overall peak to trough values observed with mScarlet-I versus mVenus in the same cells; markers indicate peaks, line indicates median; unpaired t-test, 2 tailed non-significant, ns^2=0.0873, ns^3=0.0536.

(G) Comparison of Venus::HES5 period in primary neural progenitor cultures obtained from mouse spinal cord E10.5; markers indicate cells, line indicates median; Mann-Whitney 2 tailed test, ns^4=0.8328; Heterozygous: 4 independent experiments, 78 tracks; Homozygous: 3 independent experiments, 51tracks.

Development • Supplementary information

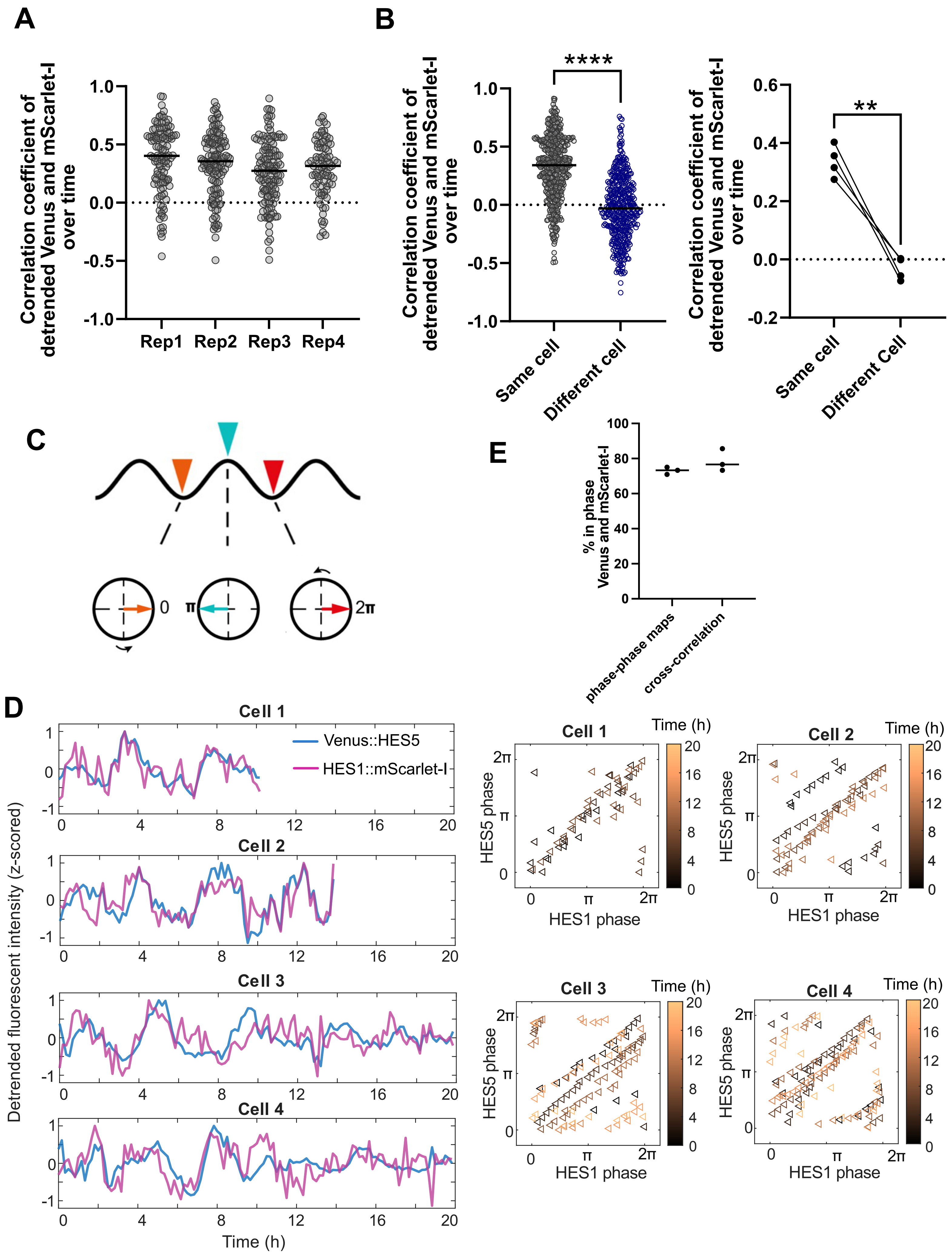
A
Correlation coefficient of detrended Venus and mScarlet-I over time
1.0
0.5
0.0
-0.5
-1.0
Rep1
Rep2
Rep3
Rep4
B
****
Same cell
Different cell
**
0.6
0.4
0.2
-0.2
Different Cell
C
0
π
2π
E
% in phase Venus and mScarlet-I
100
80
60
40
20
phase-phase maps
cross-correlation
D
Cell 1
Cell 2
Cell 3
Cell 4
Venus::HES5
HES1::mScarlet-I
Detrended fluorescent intensity (z-scored)
Time (h)
HES5 phase
HES1 phase

Fig. S3. Correlation and phase analysis of HES1 and HES5 expression in the same primary spinal cord neural progenitor cells. Related to Fig. 3.

(A) Pearsons correlation coefficient computed from detrended HES1::mScarlet-I and Venus::HES5 timeseries observed in the same nucleus over time in 4 independent experiments with a total of 446 tracks; markers indicate single nuclei, bars indicate median per experiment.

(B) Pearsons correlation coefficient from data in **(A)** compared against correlation coefficient values obtained when cross-pairing HES1::mScarlet-I in one nucleus with Venus::HES5 in another nucleus selected at random; (left panel) markers indicate individual nuclei, lines indicate median of 4 pooled independent experiments, Mann-Whitney 2-tailed test with $p<0.0001$****; (right panel) markers indicate paired medians per experiment, paired t-test, 2-tailed with $p<0.01$**.

(C) Diagram depicting phase angle reconstruction from a wave resulting in values ranging from 0 to 2pi over the course of a complete oscillation cycle.

(D) Representative examples of detrended (and z-scored) Venus::HES5 and HES1::mScarlet-I timeseries observed in the same cell and corresponding phase-phase mapping; the phase at individual timepoints in color-coded to show progression over time; individual phase observations fluctuate however are mostly distributed in the in-phase region.

Development • Supplementary information

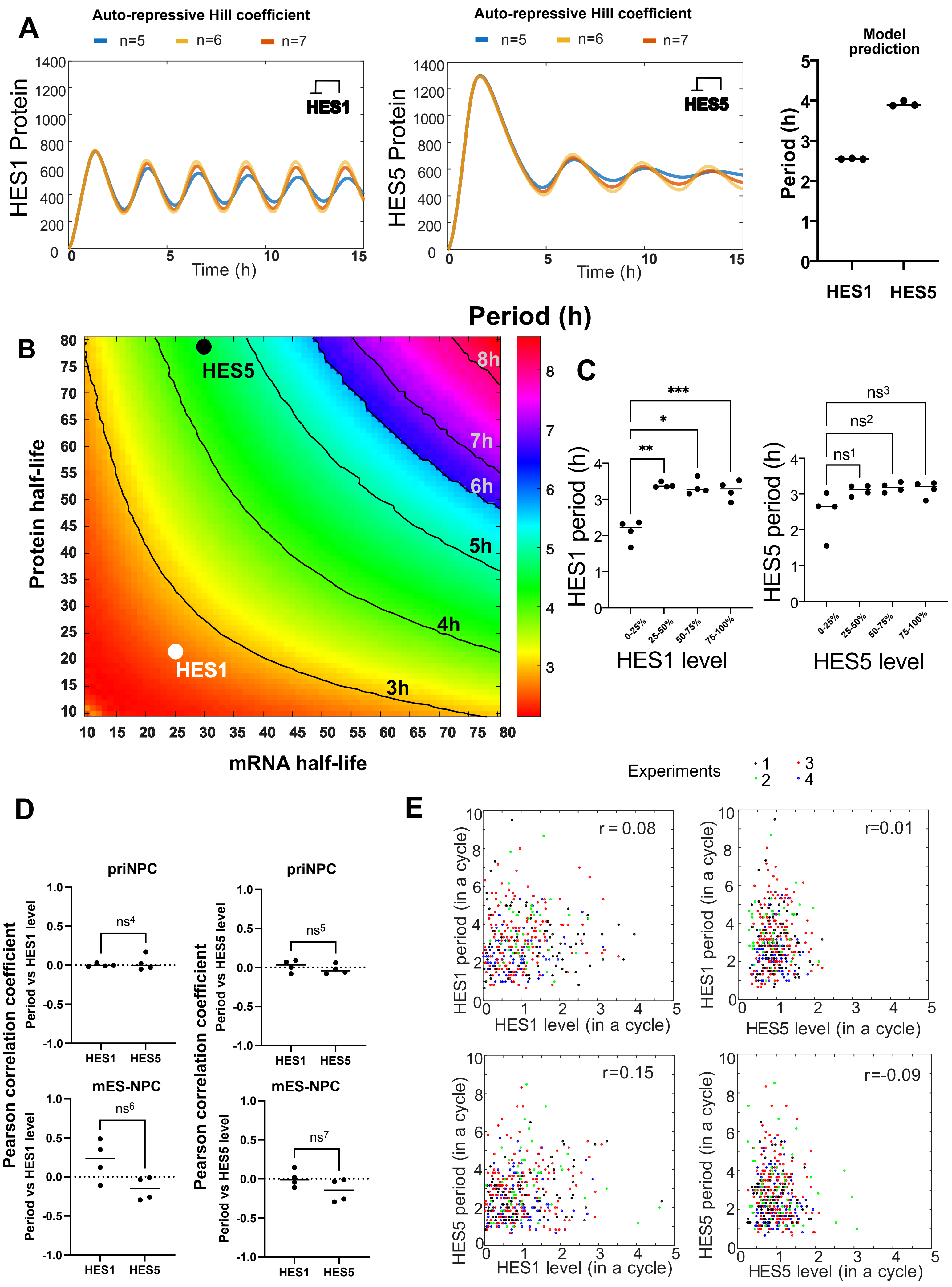

Development • Supplementary information

Fig. S4. Mathematical model predicts HES1 and HES5 period based on experimental degradation rates. Related to Fig. 4.

(A) Free-running HES model explorations at different values of auto-repression Hill coefficient values $n = 5; 6; 7$ and corresponding predicted values (measured as average of peak to peak intervals). Model parameters, HES1: $\alpha_m = \alpha_p = 1$ min^{-1} ; $\mu_m = \frac{\ln(2)}{25}$ min^{-1}; $\mu_p = \frac{\ln(2)}{22}$ min^{-1}; $P_0 = 390$; $\tau = 29$ min; HES5: $\alpha_m = \alpha_p = 1$ min^{-1} ; $\mu_m = \frac{\ln(2)}{25}$ min^{-1}; $\mu_p = \frac{\ln(2)}{80}$ min^{-1}; $P_0 = 390$; $\tau = 29$ min.

(B) Mapping of predicted period for the free running model of HES at different values of mRNA and protein half-life; markers indicate known values reported for HES1 (white marker, mRNA half-life=25min (Bonev et al., 2012); protein half-life =22min (Hirata et al., 2002)) and HES5 (black marker, mRNA half-life=30min; protein half-life =80min (Manning et al., 2019)). Other model parameters: $\alpha_m = \alpha_p = 1min^{-1}$; $P_0 = 390; n = 5$; $\tau = 29$ min.

(C) HES1 and HES5 period observed in priNPCs with different level of protein in the same HES; protein levels were binned into quartiles and median per quartile/per experiment was reported; repeated measures ANOVA with Dunnet's multiple comparison correction, $p<0.05$*, $p<0.01$**, $p<0.001$***, ns[1]=0.2989; ns[2]=0.1866; ns[3]=0.1169.

(D) Comparison of Pearsons correlation coefficient of HES1 and HES5 period versus average level of either HES measured in primary cultures (priNPC) and mES-derived NPCs using datasets in **Fig. 2**; unpaired t test, 2 tailed non-significant, ns[4]=0.6084, ns[5]=0.3976, ns[6]=0.0532, ns[7]=0.1470.

(E) Instantaneous (cycle by cycle) analysis of correlation of HES1 and HES5 period versus level of either HES measured in mES-derived NPC datasets show in **Fig. 2**; Pearsons correlation coefficient, r.

Development • Supplementary information

Supplementary Figure 5-related to Fig 4

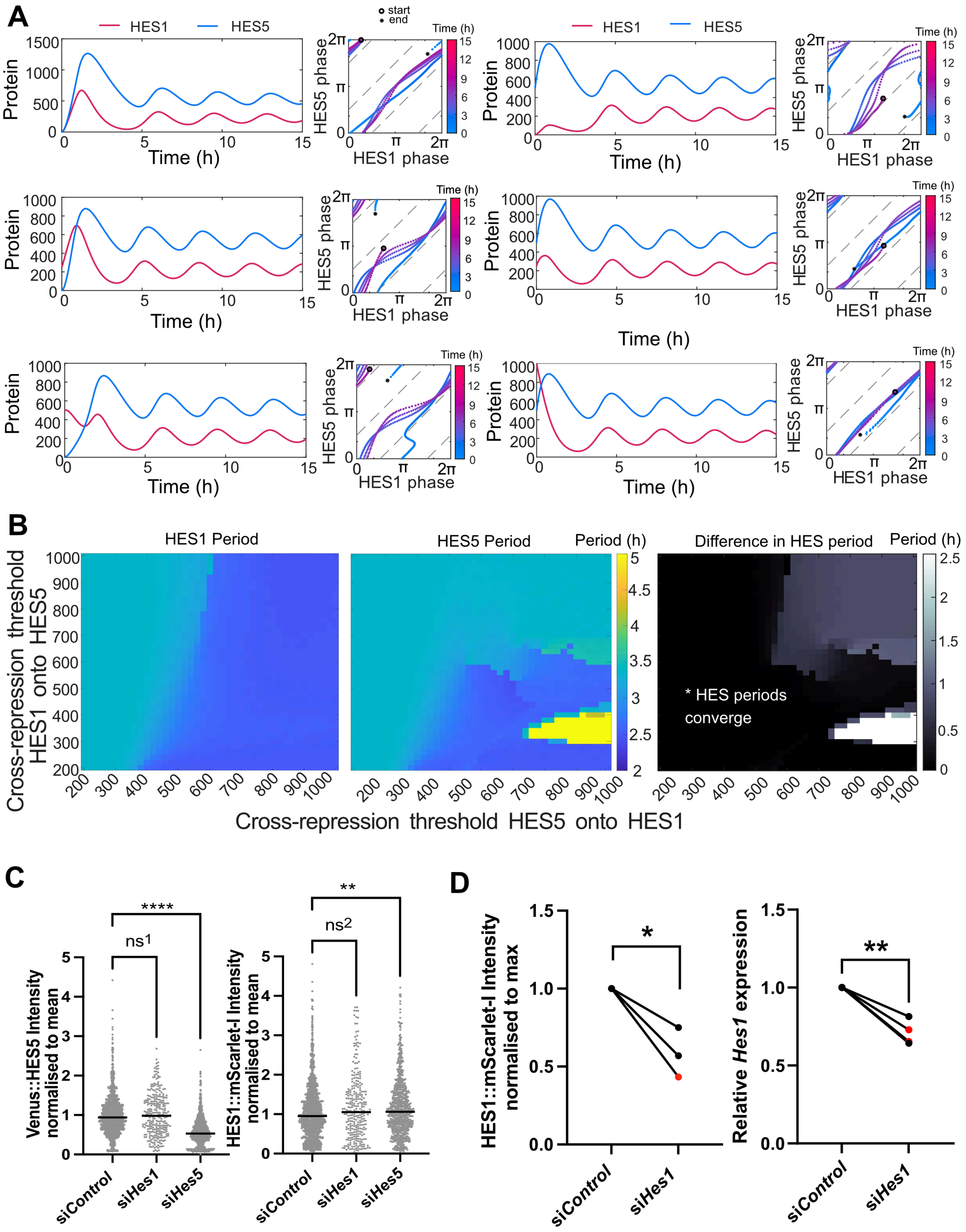

Fig. S5. Exploration of the HES1-HES5 model at different parameters and siRNA knock-down validation. Related to Fig. 4.

(A) In silico exploration of the HES1-HES5 protein dynamics at different initial conditions with corresponding phase-phase mapping; phase is color-coded to show progression over time; initial conditions (start) and end phase position is indicated with different markers. Model parameters: $P_{01} = P_{05} = P_{015} = P_{051} = 390$, $\mu_{p1} = \frac{\ln(2)}{22}$ min^{-1}, $\mu_{m1} = \frac{\ln(2)}{25}$ min^{-1}; $\mu_{p5} = \frac{\ln(2)}{80}$ min^{-1}, $\mu_{m5} = \frac{\ln(2)}{25}$ min^{-1}, $\alpha_{m1} = \alpha_{p1} = \alpha_{m5} = \alpha_{p5} = 1$ min^{-1}; $\tau = 29$ min; $n = 7; n_{15} = n_{51} = 5$.

(B) Mapping of predicted period for HES1 (left panel) and HES5 (middle panel) at different values of cross-repression threshold values P_{015} (x-axis) and : P_{051} (y-axis); the period of HES1 and HES5 converge in a wide range of parameters indicated by the difference map (right panel). Dark areas in the difference map indicate convergent period. Model parameters: : $P_{01} = P_{05} = 390$, $\mu_{p1} = \frac{\ln(2)}{22}$ min^{-1}, $\mu_{m1} = \frac{\ln(2)}{25}$ min^{-1}; $\mu_{p5} = \frac{\ln(2)}{80}$ min^{-1}, $\mu_{m5} = \frac{\ln(2)}{25}$ min^{-1}, $\alpha_{m1} = \alpha_{p1} = \alpha_{m5} = \alpha_{p5} = 1$ min^{-1}; $\tau = 29$ min; $n = 7; n_{15} = n_{51} = 5$.

(C) Quantification of HES protein expression differences in mES-derived NPC cultures in siRNA conditions measured in non-targeting control (si*Control*) and knock-down of *Hes1* (si*Hes1*) and *Hes5* (si*Hes5*) at 20-40nM after 48h; data was normalised by dividing to mean per experiment and pooled from n=3 independent experiments with 2,543 nuclei analysed.

(D) Validation of siRNA knock-down at protein and mRNA level at 20 and 40nM after 48h in neuromesodermal precursor cells (NMP, see **Fig. S1B**) that only express HES1(Materials and methods); relative protein and mRNA levels show a reduction of 30-60% with protein reducing the most at the high siRNA concentration (40nM- shown in red).

Development • Supplementary information

Supplementary Figure 6- related to Fig 5

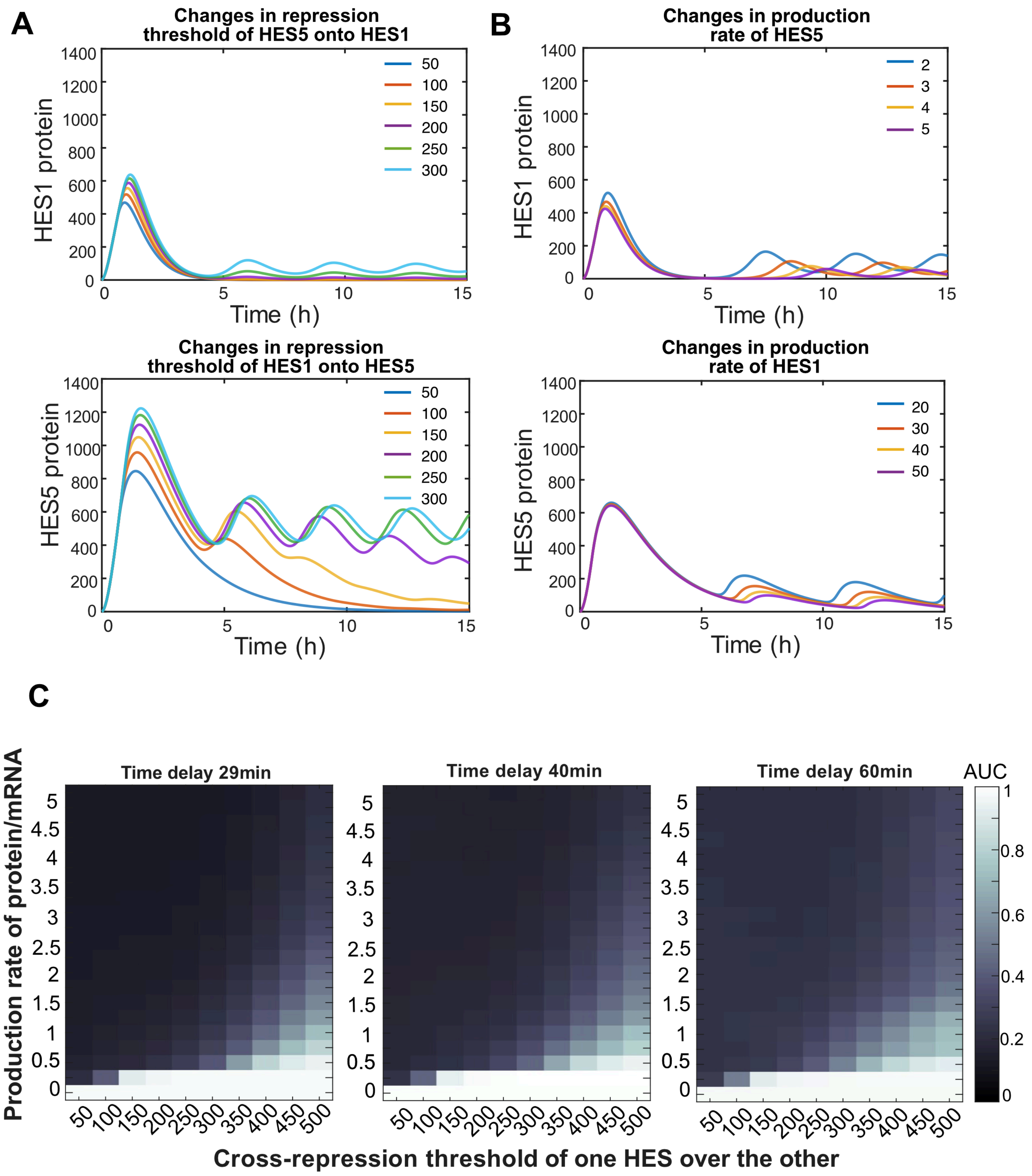

Fig. S6. Exploration of the HES1-HES5 model at different parameters and siRNA knock-down validation. Related to Fig. 5.

(A) Simulations of progressive HES5/1 repression onto HES1/5 occuring through a change in cross-repression threshold values ($P_{051}/P_{015} \in [50,300]$). Fixed model parameters: $P_{01} = P_{05} = 390$, $\mu_{p1} = \frac{\ln(2)}{22}$ min^{-1}, $\mu_{m1} = \frac{\ln(2)}{25}$ min^{-1}; $\mu_{p5} = \frac{\ln(2)}{80}$ min^{-1}, $\mu_{m5} = \frac{\ln(2)}{25}$ min $^{-1}$, $\alpha_{m1} = \alpha_{p1} = \alpha_{m5} = \alpha_{p5} = 1$ min^{-1}; $\tau = 29$ min; $n = 7$; $n_{15} = n_{51} = 5$. Top panel: $P_{015} = 390$; Bottom panel: $P_{051} = 390$. Lowering the cross-repression threshold is the most effective way to suppress either HES leading to a decrease in its area under the curve.

Development • Supplementary information

(B) Simulations of progressive HES5/1 repression onto HES1/5 occuring through a change in production rates. Fixed model parameters: $P_{015} = P_{01} = P_{05} = 390$, $\mu_{p1} = \frac{\ln(2)}{22}$ min^{-1}, $\mu_{m1} = \frac{\ln(2)}{25}$ min^{-1}; $\mu_{p5} = \frac{\ln(2)}{80}$ min^{-1}, $\mu_{m5} = \frac{\ln(2)}{25}$ min $^{-1}$,; $\tau = 29$ min; $n = 7$; $n_{15} = n_{51} = 5$. Top panel: $\alpha_{m1} = \alpha_{p1} = 1$ min^{-1}; $\alpha_{m5} = \alpha_{p5} \in [2,5]$; Bottom panel: $\alpha_{m5} = \alpha_{p5} = 1$ min^{-1}; $\alpha_{m1} = \alpha_{p1} \in [20,50]$. Increasing the production rates is an effective way to reduce the peak height of either HES leading to a decrease in its area under the curve. The more stable protein HES5 can dominate at lower production rates compared to the less stable protein HES1.

(C) Mapping of area under the curve (AUC) in HES1 protein level observed for a range of HES5 production rates ($\alpha_{m5} = \alpha_{p5} \in [0,5]$min^{-1}), cross-repression threshold values ($P_{051} \in [50,500]$) and time delay ranging from 29 to 60 min. Fixed model parameters: $P_{015} = P_{01} = P_{05} = 390$, $\mu_{p1} = \frac{\ln(2)}{22}$ min^{-1}, $\mu_{m1} = \frac{\ln(2)}{25}$ min^{-1}; $\mu_{p5} = \frac{\ln(2)}{80}$ min $^{-1}$, $\mu_{m5} = \frac{\ln(2)}{25}$ min $^{-1}$ $\alpha_{m1} = \alpha_{p1} = 1$ min^{-1}; $n = 7; n_{15} = n_{15} = 5$. Dark areas correspond to dominant HES.

Development • Supplementary information

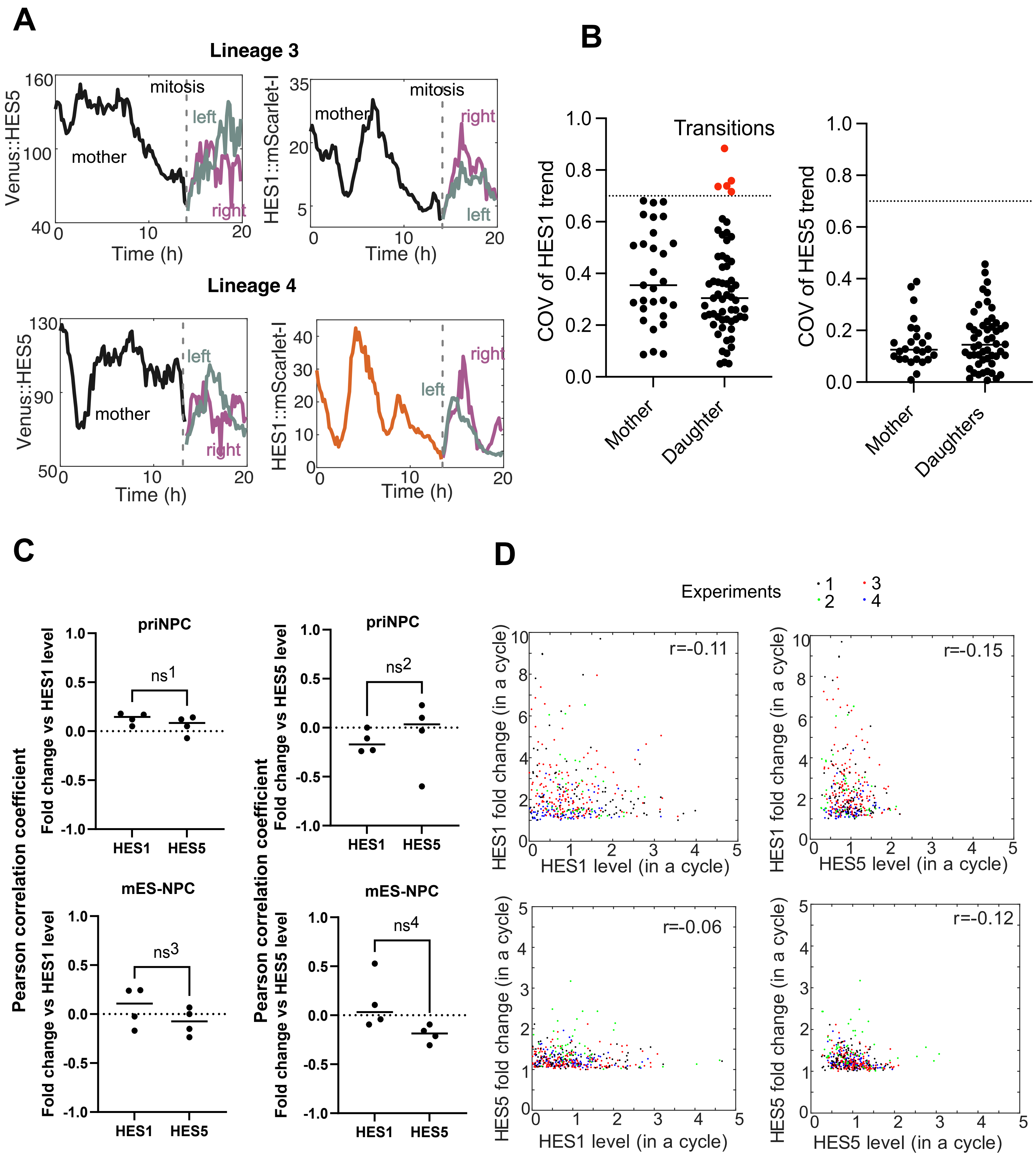

Fig. S7. Lineage tracked HES1 and HES5 and correlation analysis of fold change versus level. Related to Fig. 6. **(A)** Representative examples of HES1::mScarlet-I and Venus::HES5 fluorescent intensities observed in dividing mES-derived neural progenitor lineages comprising mother cell (division time unknown) and 2 daughter cells (left and right); intensities of mScarlet-I and Venus are not directly comparable however the same fluorophore can be compared between mother, left and right; lineages3&4 indicate that both left and right retain levels of HES1 and HES5 comparable to the mother cell following division; lineages 3&4 were collected in the same experiment as lineages 1&2 included in **Fig. 6A.**

(B) Quantification of variability in the trend of HES1 and HES5 to identify cells that transition (Materials and methods). Using the trend of each cell, coefficient of variation (CoV=standard deviation over mean) in both mother and daughter cells was computed; we identified that 5 daughter cells (corresponding to 5 divisions) had CoV above 0.7 including examples in **Fig. 6A;** the majority of daughter and mother cells including examples shown in **(A)** had CoV<0.7 indicating no transition; HES5 CoV was low throughout.

(C) Comparison of Pearsons correlation coefficient of HES1 and HES5 maximum fold change versus average level of either HES measured in primary cultures (priNPC) and mES-derived NPCs using datasets in **Fig. 2**; unpaired t test, 2 tailed non-significant, ns^1=0.2577, ns^2=0.7272, ns^3=0.2646, ns^4=0.0750.

(D) Instantaneous (cycle by cycle) analysis of correlation of HES1 and HES5 fold change versus level of either HES measured in mES-derived NPC datasets show in **Fig. 2**; Pearsons correlation coefficient, r.

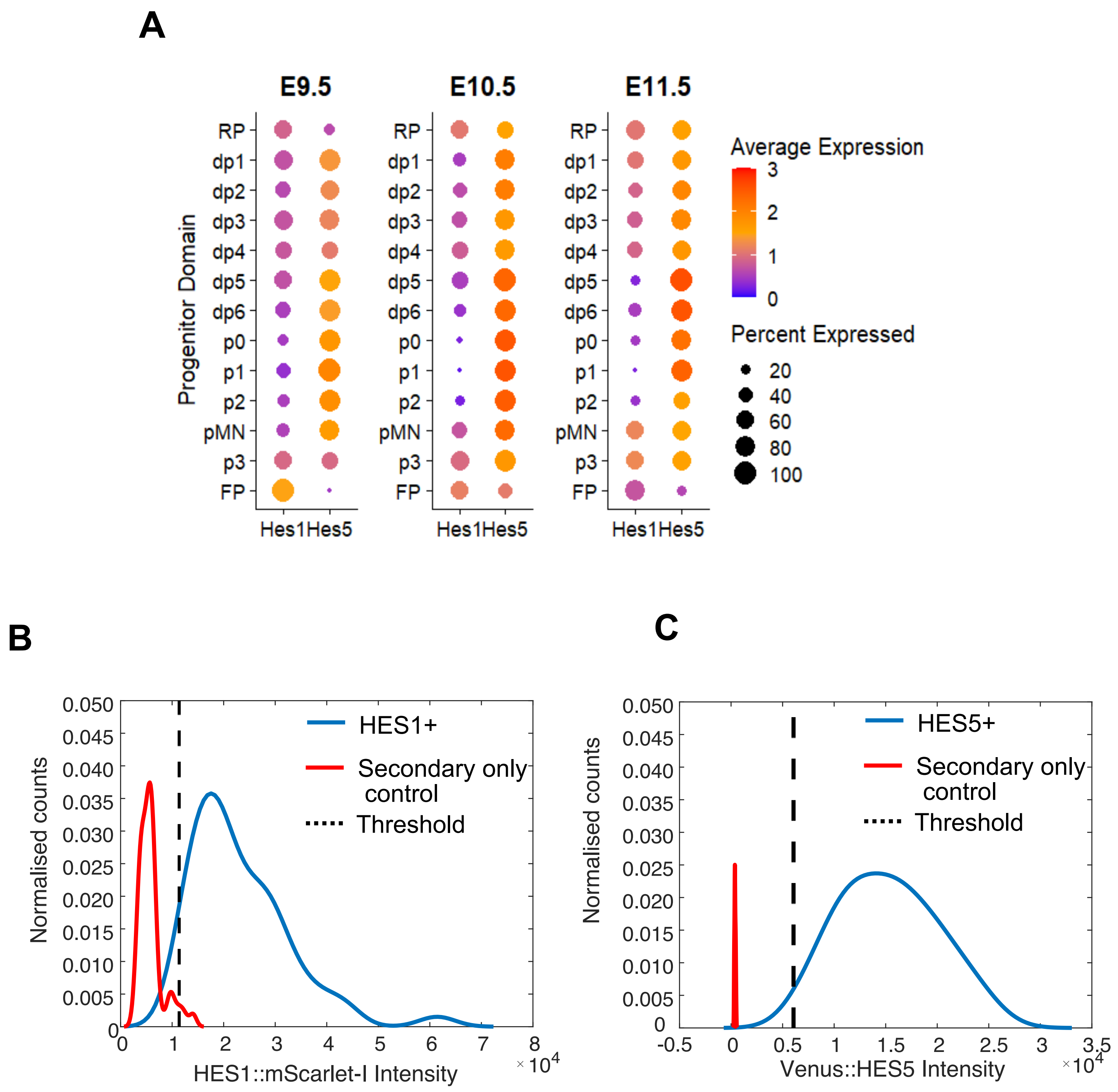

Fig. S8. Quantification of HES1+ and HES5+ cells from tissue data. Related to Fig. 7.

(A) Quantification of *Hes1* and *Hes5* expression in progenitors in the dorso-ventral axis of mouse embryonic spinal cord between E9.5 to E11.5 based on **t**ranscriptomic data from (Delile et al., 2019); the data indicates heterogeneity in the timing of different progenitor groups spend in the hybrid state.

(B-C) Intensity threshold for selecting HES1+ and HES5+ from data in **Figure 6A-E**; the distribution of nuclear intensity values for HES1:mScarletI detected by anti-RFP and Venus::HES5 detected by anti-GFP with intensity above threshold (dashed line) is compared against the distribution of nuclear intensities observed in slices with secondary only control (no anti-RFP, no anti-GFP).

Table S1. List of reagents

Reagent or Resource	Source	Catalogue No.
**Antibodies**		
Mouse monoclonal anti-PAX3/PAX7 (1:200)	R & D Biotech	MAB2457<br>***RRID:AB_2159398***
Rabbit polyclonal anti-SOX2 (1:200)	Abcam	ab97959<br>***RRID:AB_2341193***
Anti-RFP (1:500)	Proteintech	5F8<br>***RRID: AB_2336064***
Anti-GFP (1:200)	Abcam	ab13970<br>***RRID: AB_300798***
Anti-GFP (1:100)	Thermo Fisher	A-11122<br>***RRID: AB_221569***
Anti-GFP (1:500)	Roche	11814460001<br>***RRID: AB_390913***
Donkey anti-rabbit Alexa Fluor 488 (1:500)	Thermo Fisher Scientific	A21206<br>***RRID: AB_2535792***
Donkey anti-mouse Alexa Fluor 647 (1:500)	Thermo Fisher Scientific	A31571<br>***RRID: AB_162542***
Donkey- anti chicken Alexa Fluor 488 (1:500)	Jackson ImmunoResearch	703-545-155<br>***RRID: AB_2340375***
Donkey anti-Rat DyLight 550 (1:800)	Thermo Fisher Scientific	SA5-10027<br>***RRID: AB_2556607***
Goat anti-mouse Alexa Fluor 647 (1:500)	Thermo Fisher Scientific	A-21235<br>***RRID:AB_2535804***
Goat anti-rabbit Alexa Fluor 405 (1:500)	Thermo Fisher Scientific	A-31556<br>***RRID:AB_221605***
**Knock-down Reagents**		
ON-TARGETplus Mouse Hes1 siRNA	Horizon Discovery	L-060177-01-0005
ON-TARGETplus Mouse Hes5 siRNA	Horizon Discovery	L-060490-01-0005
ON-TARGETplus Non-targeting Control Pool	Horizon Discovery	D-001810-10-05
Lipofectamine RNAiMax transfection reagent	Thermo Fisher Scientific	13778030
OptiMEM reduced serum media	Gibco	31985062
**Cell Culture and IF Reagents**		
DMEM/F12	Sigma	D6421
Neurobasal	Gibco	21103-049
ESGRO 2i LIF	Sigma	SF016-100
N2 supplement	Gibco	17502048
B27 supplement	Gibco	17504-044
GlutaMAX	Gibco	35050-038
D-Glucose solution	Sigma	G8644
Bovine Albumin (BSA) Fraction V 7.5%	Gibco	15260-037
MEM Non-essential Amino Acid Solution	Sigma	M7145
Phosphate buffer saline (PBS)	Sigma	D8537
Basic fibroblast growth factor (bFGF)	PeproTech	100-18B
Mouse EGF recombinant protein	PeproTech	315-09-500UG
CHIR-99021	Sigma	SML1046-5MG
LIF	Bio-Techne	8878-LF-025
PD0325901	Sigma	PZ0162-5MG
Retinoic acid (RA)	Sigma	R2625
Accutase	Sigma	A6964
Laminin	Sigma	L2020
Laminin 511	Biolamina	LN511-0202
Matrigel	Corning	356234
Donkey serum	Sigma	D9663
Fibronectin	Millipore	FC010

Development • Supplementary information

KSOM	Millipore	MR-101-D
Acid Tyrode's solution	Millipore	MR-004-D
Paraformaldehyde (PFA)	Thermo Fisher Scientific	28908
Triton X	Sigma	T9284-500ML
2-mercaptoethanol	Invitrogen	31350-010
Rabbit anti-mouse antiserum	Sigma	M5774
Guinea pig serum	Sigma	C5405
Hoechst 33342 Solution	Thermo Fisher Scientific	62249
**Other**		
CELLview 35-mm cell culture dish	Greiner BIO-ONE	627860
CELLview 35-mm cell culture dish (4 compartments)	Greiner BIO-ONE	627870
Superfrost Plus Adhesion microscope slides	Eprendia	J1800AMNZ
Gold Antifade	Thermo Fisher	P36930

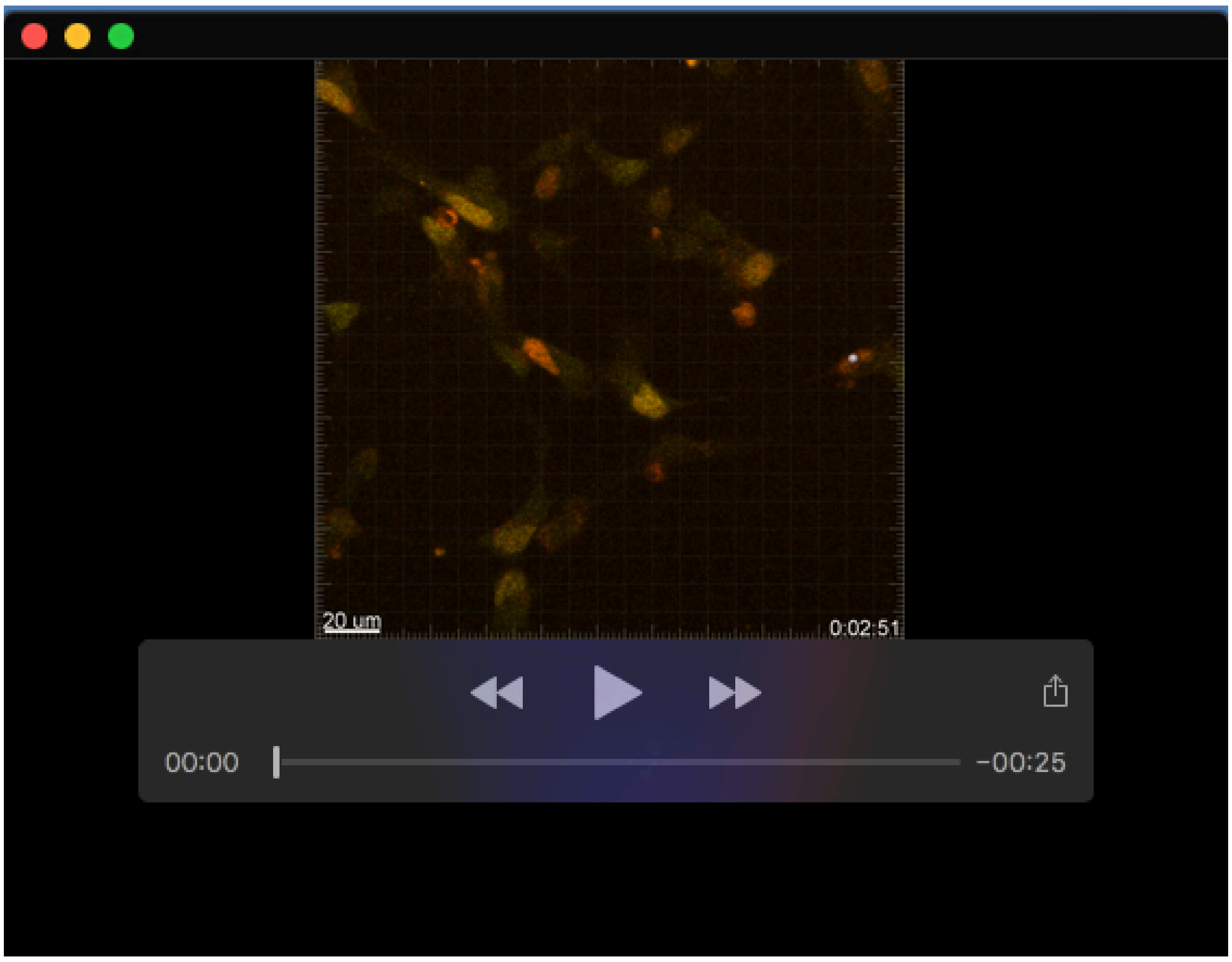

Movie 1. Timelapse video of primary neural progenitor cultures expressing Venus::HES5 and HES1::mScarlet-I. Duration approximately 18h.

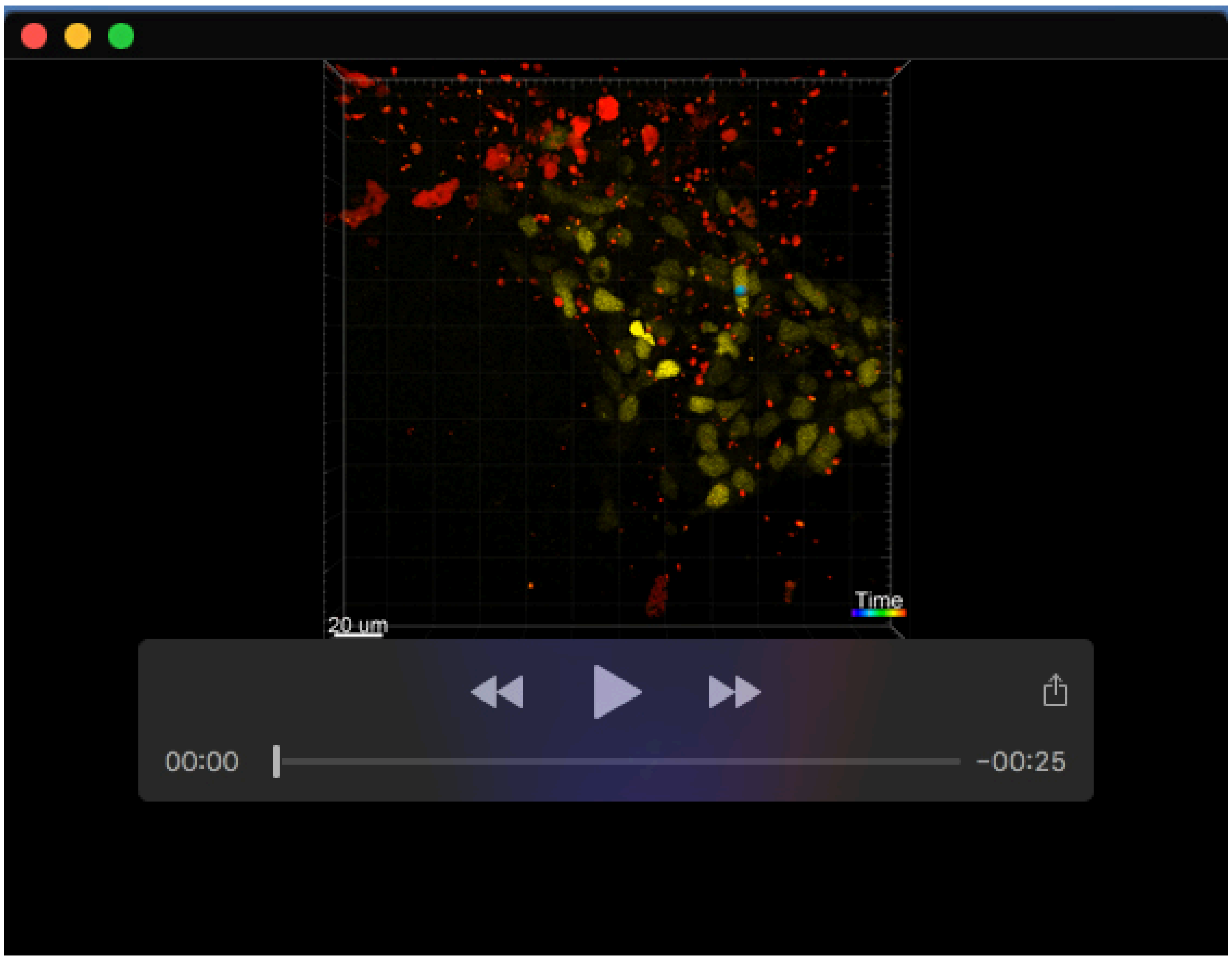

Movie 2. Timelapse video of mES-derived neural progenitor cell culture expressing Venus::HES5 and HES1::mScarlet-I. Duration approximately 15h.

Development • Supplementary information
