# Supplementary material for: Oscillatory co-expression of HES1 and HES5 enables a hybrid state in a cross-repressive transcription factor regulatory motif
Source: Development. 2026 Jun 10;153(11):dev204969. doi: 10.1242/dev.204969 (PMC13286377; doi:10.1242/dev.204969)
Supplement: Supplementary file 1 [file develop-153-204969_review_history.pdf]

Oscillatory co-expression of HES1 and HES5 enables a hybrid state in a cross-repressive transcription factor regulatory motif

Veronica Biga, Anzy Miller, Anoushka Kamath, Robert Lea, Ying Q. P. Mak, Antony D. Adamson, Elli Marinopoulou, Paul François, Nancy Papalopulu and Cerys S. Manning
DOI: 10.1242/dev.204969

Editor: James Briscoe

Review timeline
Original submission: 22 May 2025
Editorial decision: 15 June 2025
First revision received: 1 April 2026
Accepted: 25 April 2026

Original submission

First decision letter

MS ID#: dev.204969

MS TITLE: Oscillatory co-expression of HES1 and HES5 enables a hybrid state in a bistable transcription factor regulatory motif

AUTHORS: Veronica Biga, Anzy Miller, Anoushka Kamath, Ying Q. P. Mak, Antony D. Adamson, Elli Marinopoulou, Paul Francois, Nancy Papalopulu and Cerys S. Manning

Dear Dr Manning,

I have now received all the referees' reports on the above manuscript, and have reached a decision. The referees' comments are appended below, or you can access them online: please go to:

As you will see, the referees express considerable interest in your work, but have some significant criticisms and recommend a substantial revision of your manuscript before we can consider publication. Please clarify whether you propose three discrete dynamical regimes or if the oscillatory state represents a transient phase leading to stable states, as indicated by Referee 3. Your mathematical model would benefit from deeper exploration, particularly regarding phase relationships that show apparent discrepancies between experiments, parameter sensitivity analysis, and quantitative comparison of predicted versus observed oscillation damping timescales. The reviewers seek more direct validation of the HES5-HES1 entrainment model, potentially through knockout experiments or bidirectional effects analysis. An important question to address is whether the observed period changes reflect true entrainment or correlate with expression level changes. Additionally, strengthening your discussion of the biological mechanisms underlying parameter changes during neural differentiation, specifically how these enable transitions from hybrid to single-factor states, would strengthen the study. Finally, please consider the reviewers' concerns about the biological significance of detrended oscillatory data, as the removed trends may themselves encode important state transition information.

If you are able to revise the manuscript along the lines suggested, which may involve further experiments, I will be happy to receive a revised version of the manuscript. Your revised paper will be re-reviewed by one or more of the original referees, and acceptance of your manuscript will depend on your addressing satisfactorily the reviewers' major concerns. Please also note that

© 2026. Published by The Company of Biologists under the terms of the Creative Commons Attribution License (https://creativecommons.org/licenses/by/4.0/).

Development will normally permit only one round of major revision. If it would be helpful, you are welcome to contact us to discuss your revision in greater detail. Please send us a point-by-point response indicating your plans for addressing the referees' comments, and we will look over this and provide further guidance.

Please attend to all of the reviewers' comments and ensure that you clearly highlight all changes made in the revised manuscript. Please avoid using 'Tracked changes' in Word files as these are lost in PDF conversion. I should be grateful if you would also provide a point-by-point response detailing how you have dealt with the points raised by the reviewers in the 'Response to Reviewers' box. If you do not agree with any of their criticisms or suggestions please explain clearly why this is so.

Reviewer 1

Advance summary and potential significance to field

Biga et al. use a combination of advanced live cell imaging of endogenous reporters and computational modelling to understand how oscillatory behaviours influence the properties of cross-repressive fate determinants. To this end, they focus on the cross-repressive and oscillatory Notch target genes HES1 and HES5 in primary NPCs derived from the embryonic spinal cord and dorsal neural progenitors differentiated from mouse embryonic stem cells. In both systems, a large portion of cells co-express both proteins and surprisingly both transcription factors oscillate in-phase, with the faster oscillator HES1 adopting the period of the slower oscillator HES5. Based on these observations the authors suggest that oscillations facilitate a hybrid state, in which cross-repressive transcription factors can co-exist. To gain further insight into how differences in production and degradation rates affect period duration and cross-repressive behaviour, the authors turned to computational modelling comparing free-running and coupled HES1-HES5 dynamics. They demonstrate that by coupling between both oscillators the slower oscillator HES5 entrains the faster oscillator HES1. Moreover, the oscillatory co-existing regime can switch to a mutually-exclusive regime through a change in repression threshold and increase in abundance of either transcription factor. Lastly, the authors provide experimental evidence for this transition out of the hybrid state by demonstrating that the HES1/HES5 hybrid state resolves into discrete single HES-positive expression domains during spinal cord development.

Overall, this is an interesting and well-written manuscript that I very much enjoyed reading and that in my opinion will be of general interest for the readership of Development. The finding that oscillations can facilitate the co-existence of cross-repressive transcription factors is interesting and likely interesting for a wide range of other developmental systems. I therefore only have a few minor comments that the authors will be able to address without problems during the review process.

Comments for the author

1) Fig. 1F,G: It appears that there were significantly less cells analysed from the ES-NPC differentiations than from the spinal cord extracted primary-NPCs. Given that it should have been much easier to generate large numbers cells in the ES-NPC differentiations, I wonder what's the reason for this discrepancy?

2) Fig. 2: Why do the authors not show the comparison of multiple experiments in the ESC-derived NPCs, as they show for the primary NPCs in the right panel in F?

3) Fig. 3E: The labels for 'Scarlet Intensity' is different on the bottom left graph. Related to this, the authors refer throughout the manuscript to the mScarlet fusion in multiple different ways (Scarlet, mScarlet, mScarletI, mScarlet-l,…) and should unify these notations.

4) First sentence of the chapter 'A coupled Hes1-Hes5 oscilation modell…' on pg. 9 requires a reference to a figure. Similarly, the statements in the last sentences of the first paragraph on pg. 10 ('When the Hes5 levell…'until the end of the paragraph) should be supported by a figure reference.

© 2026. Published by The Company of Biologists under the terms of the Creative Commons Attribution License (https://creativecommons.org/licenses/by/4.0/).

5) Top of Pg. 11: The authors state that they see occasional transitioning from fast to slow HES1 oscillations as a consequence of HES5 upregulation in some pri-NPCs (Fig. 4F). It would be nice if the authors could provide a quantification for how often this phenomenon occurs.

6) Pg 14: 'important insights into how the two Hes oscillators interact in a single cell'. Because Hes proteins can form homo- and heterodimers, 'interact' may be misleading here.

7) In the section 'Establishment and maintenance of primary neural progenitor cultures' in the Materials and Methods, the authors refer to dissecting LGEs of e13.5 mouse embryos, which they have not done in this version of the manuscript.

8) The authors provide validation for the entrainment of HES1 by HES5 in the manuscript by showing that the period of the HES1 oscillations is significantly shorter in the lowest HES5 quartile group (bottom of pg. 10) and that they see occasional transitioning from fast to slow HES1 oscillations as HES5 is upregulated in some pri-NPCs (top of pg 11). While these observations support their model, it would be great if the authors could provide a more direct validation. This could be for example done by demonstrating that cells acquire faster HES1 oscillations in ES-NPCs differentiated from HES5 knock-out cells, while HES5 oscillation period should not be affected in HES1 knock-outs. Alternatively, the authors could check if they, as in the embryonic spinal cord, observe more frequent transitions from the HES1-HES5 hybrid state to a HES1 single state, if they analyse the dorsal ES-NPC differentiations at a slightly later stage of the differentiation protocol, and if this increased rate of transition is associated with an increased oscillation frequency.

Reviewer 2

Advance summary and potential significance to field

Biga et al. address in their manuscript "Oscillatory Co-expression of Hes1 and Hes5 enables a hybrid state in a bistable transcription factor regulatory motif" how multiple Hes transcriptional repressors interact and influence each other in mouse neural progenitors. Hes proteins are dynamic in many cell types due to their negative autoregulation and co-expression is a common feature. How the parameters of each delayed negative feedback loop affect each other due to cross-repression has not been addressed yet. Here, the authors quantified Hes1 and Hes5 dynamics in the developing neural tube both in primary cell culture and in vitro differentiated cells. They then use a theoretical model to explore the parameter space and determine when and how one TF "wins". They then correlate this finding to quantifications in cells. This paper is very relevant for neural tube development and other processes containing multiple oscillatory systems, e.g. the segmentation clock.

Comments for the author

There are a few points that would be worth addressing before publication:

Major points:
- The manuscript deals with the Hes1-Hes5 network as isolated system. The authors should at least mention that other factors such as signalling, other TFs, epigenetic changes etc. probably have an influence and will probably modulate the oscillation period as well. Something the authors mention in the result section: "These observations are consistent with the fact that the mES differentiation protocol enriches for dorsal types (Gupta et al., 2022) (identified by expression of PAX3/7) where HES1 is more widely expressed compared to ventral (Sagner et al., 2018); in contrast, the priNPCs could be ventral or dorsal, with HES5 being expressed in both areas (Manning et al., 2019)."
In the same regard, a discussion of how the parameters change in the context of neural differentiation should be added. Why does the cross-repression threshold change, as cells differentiate, which allows the stabilization of one TF over the other. They mention in the results that "experimental evidence suggests parameters can change (…) following cell division". In the discussion, the authors should discuss and speculate what the mechanism for changing parameters and thereby changing periods etc is. This would then also link back to the process of neural tube development, which is only superficially addressed in the discussion now.

© 2026. Published by The Company of Biologists under the terms of the Creative Commons Attribution License (https://creativecommons.org/licenses/by/4.0/).

- In the introduction, the authors "present the challenge" of co-existence of cross-repressive TFs and fate determinants. Something that is observed in many differentiation processes for various TFs. What is special about Hes proteins is that they are not only cross-repressive but also auto-repressive. This makes their dynamics different from other cross-repressive TF pairs. This is an aspect the authors should highlight and could also address in their discussion. e.g. What do we learn from this paper for other non-autorepressive TF pairs?
- In Figure 3, signal from both Hes1 and Hes5 are quantified in the same cells and phase-phase maps are generated. It would be interesting to know what the heterogeneity between cells is. It is stated that there is a "presence of stochasticity". Is this because oscillations are in-phase in some cells and not in others or is it because it is variable within single cells? Do variations correlate with Hes1 or Hes5 levels or differentiation state? This data should be present in their data.
- In Figure 4, the authors discuss how the presence of one TF influences the oscillations of the other. Using a coupled-oscillator model, they conclude that the "slow oscillator Hes5 cannot speed up", which is why they only test the effect of Hes5 levels on Hes1 (and Hes5) oscillation period. They should also check the other way around. As they have Hes5+/Hes1- cells in their dataset, they could check how the presence of Hes1 influences Hes5 oscillations. It is still possible that Hes1 also entrains Hes5 and leads to a slight decrease in the Hes5 oscillation period. Considering that Hes5 is also oscillatory in mouse somitogenesis with a general period of 2.5 hours, a possible decrease in the Hes5 oscillation period is in likely.
- Figure 1B, C: Cell density in mESC-derived NPCs is higher than for primary NPCs. Are these images indeed representative? Does cell density explain slight differences in oscillation parameters? An easy way to address this would be to quantify dynamics in less dense regions.
- Hes5 Hes1 reporter mice were used heterozygously, while mESC-derived NPCs were homozygous. The authors could clarify what the phenotype of double hom mice is? Is there any difference in period?

Minor points:
- In the introduction, the authors discuss the presence of hybrid states identified in scRNAseq experiments. However, protein is not always the same as RNA. Maybe phrase more carefully?
- The authors quantify the peak-to-trough fold-change, which is a measure of the oscillation amplitude. They also Hilbert transform to determine the oscillation phase. They could use the same approach to determine the (instantaneous) amplitude (and period), which they could then link to Hes1/ Hes5 expression levels over time (as in Figure 4F).
- Results part: "Like priNPCs, in mES-derived NPCs HES1 oscillations were more
- frequent, and ". What is meant with more frequent? Oscillations occurred more frequently or the frequency was higher? It might be good to re-phrase to prevent confusion.
- I don't see a section on period quantification in the method section. This should be added.
- Figure 6: Consider adding a scheme where along the DV axis of the neural tube Hes5 and Hes1 are expressed (maybe even over development).
- Typo in Methods: "514nm laser (Venus). was de Z-sections covering the"

Other point:
The model based on a coupled oscillator model makes sense to me. However, I am not judging this from a physicist's point of view.

Reviewer 3

Advance summary and potential significance to field

In this manuscript, the authors address a longstanding conundrum: how cross-repressive transcription factors such as Hes1 and Hes5, known to encode distinct downstream cell fates, can coexist dynamically within single cells in the developing neural tube. Specifically, they show that oscillatory expression allows these factors to be co-expressed in a temporally coordinated fashion, effectively delaying cell fate resolution and enabling hybrid states. The authors support this hypothesis through a combination of live-cell imaging and a mathematical model grounded in biophysical parameters. The model captures both known and emergent behaviours, including oscillator entrainment and how variations in interaction strength and degradation rates affect cell fate outcomes.

© 2026. Published by The Company of Biologists under the terms of the Creative Commons Attribution License (https://creativecommons.org/licenses/by/4.0/).

Overall, the study is solid and provides very interesting insights into temporal regulation of cell fate. However, several conceptual and technical aspects could benefit from clarification or deeper analysis, particularly regarding the definition of cell states and the interpretation of oscillatory dynamics.

SUGGESTIONS TO AUTHORS

Major Comments

1) The manuscript would benefit from clarifying how the authors define cellular states, especially in the abstract and discussion. Are the authors proposing three discrete dynamical regimes (e.g., two stable states and one hybrid), or is the oscillatory state a transient phase (e.g., a damped or spiral attractor) en route to one of the stable states? This distinction has important implications for how we conceptualise cell states in development, which are rarely truly stationary. The same apply for the mathematical model, is there any stable oscillation state observed, or there is always a single attractor in the system?

2) If I understood correctly, trends are removed before analysing oscillatory characteristics. However, these trends themselves may encode important biological information. For instance, changes in the mean levels of HES proteins could reflect cell state transitions. In many traces (e.g., HES1 in Fig. 3A), changes in the trend exceed the oscillation amplitude. Have the authors analysed these trends separately? Are they correlated with period or cell decision? Are they captured by the mathematical model?

3) The model is interesting, and is simple enough to gain mechanistic understanding of the emerging behaviours, but could be explored in greater depth:
3a) The initial large peak in protein levels appears to result from initial conditions (zero protein). How sensitive are the dynamics to this choice, and how do they compare to experimental starting conditions?
3b) Does the model predict timescales for oscillation damping? Can these be compared quantitatively to the experimental data?
3c) Phase relationships in Fig. 3F show apparent synchrony, but in Fig. 4B, a phase lag of ~π/2 is observed. Can the authors explain this discrepancy? Phase diagrams for the model across different parameter regimes would help clarify when and how in-phase locking emerges.

4) The observation that HES1 period increases in the presence of HES5 is compelling. However, since HES1 and HES5 levels are positively correlated, could the change in period be simply a function of HES1 expression levels? Would plotting HES1 period against HES1 amplitude alone in Fig 4D (rather than against HES5 levels) show a similar trend?

5) The manuscript refers to fate decisions at the point of cell division. If this refers to a specific hypothesis regarding levels or phases of HES oscillations, a statistical analysis addressing this directly would help clarify the claim

6) The model shows that increasing repression thresholds or mRNA production can push the system out of the oscillatory regime toward dominance by a single TF. This is interesting, but likely not the only possible mechanism. For instance, changes in degradation rates (potentially through post-translational modifications or heterodimer formation) or delays in transcriptional feedback (the delay parameter imposed in the differential equaion) could achieve similar transitions. Can the authors comment on the specificity of the mechanisms they identify, given the available data?

Minor Comments

1. In the discussion of intermediate states, the authors could reference examples of bistable circuits that exhibit a third, stable co-expressing state via self-activation, such as Guantes & Poyatos (PLoS Comp Biol 2008, doi:10.1371/journal.pcbi.1000235), as well as synthetic gene circuit implementations of such circuits (Wu et al. eLife 2017;6:e23702. DOI: 10.7554/eLife.23702).

2. The identification of "dominant" cells in the experimental data could be more clearly explained. This also links back to the definition of cell states (see Major Comment 1).

© 2026. Published by The Company of Biologists under the terms of the Creative Commons Attribution License (https://creativecommons.org/licenses/by/4.0/).

3. The methodology for oscillation detection could be described more precisely:
3a) Statements such as "data was de-trended using a 7.5 lengthscale parameter" are too vague for reproducibility.
3b) The use of Hilbert transforms for phase detection could be complemented by more intuitive analyses, such as computing time-lagged correlations (e.g., $\langle H1(t)H5(t-\tau)\rangle$ as a function of τ). This would give direct insight into phase relationships and how they evolve over time.
4. The sentence "which produces oscillations of 2-3X in amplitude (that decay over time as the model is deterministic)" is misleading. Determinism alone does not preclude sustained oscillations e.g., the repressilator is a deterministic oscillator. The decay observed here is likely due to the system's parameters leading to damped dynamics.
5. "Repress mRNA of the other gene by 50%", I think the authors mean "Repress mRNA transcription rate of the other gene by 50%".

First revision

Author response to reviewers' comments

We thank all reviewers for their insightful and constructive comments, which helped us produce a better manuscript. Below is a point-by point response of how we addressed each comment in the revised manuscript.

Reviewer 1: SUMMARY OF THE ADVANCE MADE IN THIS PAPER AND ITS POTENTIAL SIGNIFICANCE TO THE FIELD

Biga et al. use a combination of advanced live cell imaging of endogenous reporters and computational modelling to understand how oscillatory behaviours influence the properties of cross-repressive fate determinants. To this end, they focus on the cross- repressive and oscillatory Notch target genes HES1 and HES5 in primary NPCs derived from the embryonic spinal cord and dorsal neural progenitors differentiated from mouse embryonic stem cells. In both systems, a large portion of cells co-express both proteins and surprisingly both transcription factors oscillate in-phase, with the faster oscillator HES1 adopting the period of the slower oscillator HES5. Based on these observations the authors suggest that oscillations facilitate a hybrid state, in which cross-repressive transcription factors can co-exist. To gain further insight into how differences in production and degradation rates affect period duration and cross- repressive behaviour, the authors turned to computational modelling comparing free- running and coupled HES1-HES5 dynamics. They demonstrate that by coupling between both oscillators the slower oscillator HES5 entrains the faster oscillator HES1. Moreover, the oscillatory co-existing regime can switch to a mutually-exclusive regime through a change in repression threshold and increase in abundance of either transcription factor. Lastly, the authors provide experimental evidence for this transition out of the hybrid state by demonstrating that the HES1/HES5 hybrid state resolves into discrete single HES-positive expression domains during spinal cord development.

Overall, this is an interesting and well-written manuscript that I very much enjoyed reading and that in my opinion will be of general interest for the readership of Development. The finding that oscillations can facilitate the co-existence of cross- repressive transcription factors is interesting and likely interesting for a wide range of other developmental systems. I therefore only have a few minor comments that the authors will be able to address without problems during the review process.

SUGGESTIONS TO AUTHORS

1) Fig. 1F,G: It appears that there were significantly less cells analysed from the ES-NPC differentiations than from the spinal cord extracted primary-NPCs. Given that it should have been much easier to generate large numbers cells in the ES-NPC differentiations, I wonder what's the reason for this discrepancy?

© 2026. Published by The Company of Biologists under the terms of the Creative Commons Attribution License (https://creativecommons.org/licenses/by/4.0/).

In the updated manuscript, we have increased the total sample size of mES-derived NPC to 1,987 cells, n=3 repeats which is comparable to the priNPC dataset (approx. 2,000 cells) (**Rebuttal Fig 1, new Fig 1E,G**).

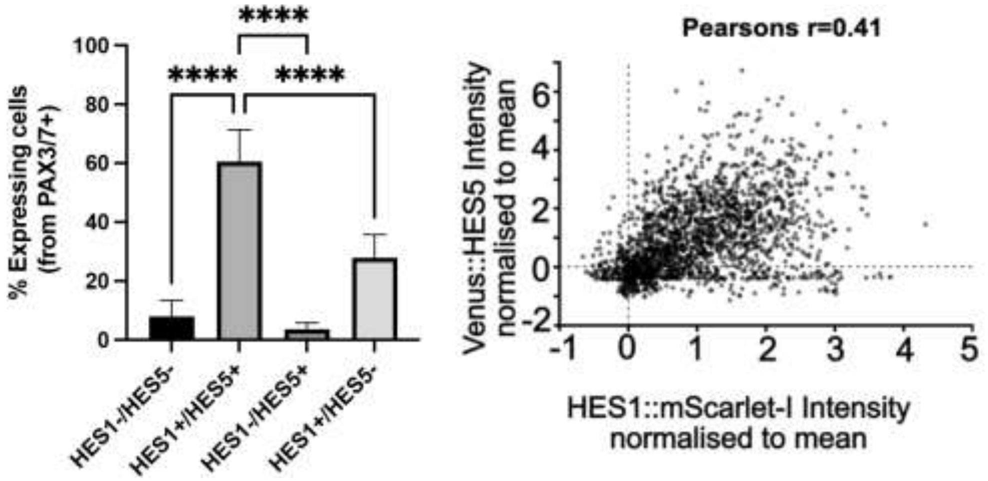

Rebuttal Fig 1. Percentages of double or single HES expressing mES-derived NPCs identified with progenitor marker PAX3/7 (**new Fig 1E**). Scatterplot data of HES1 vs HES5 intensity correlations in the same cells (**new Fig 1G**).

2) Fig. 2: Why do the authors not show the comparison of multiple experiments in the ESC-derived NPCs, as they show for the primary NPCs in the right panel in F?

As suggested, we have included the comparison of period median for multiple experiments of mES-derived NPCs including an additional 4th repeat experiment (**Rebuttal Fig 2, new Fig 2I**). This strengthened our previous result.

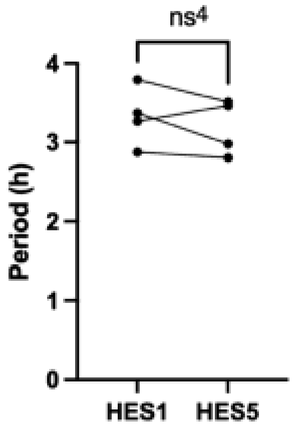

Rebuttal Fig 2. Comparison of median period per experiment in HES1 versus HES5 monitored in mES-derived NPCs (**new Fig 2I- right panel**).

3) Fig. 3E: The labels for 'Scarlet Intensity' is different on the bottom left graph. Related to this, the authors refer throughout the manuscript to the mScarlet fusion in multiple different ways (Scarlet, mScarlet, mScarletI, mScarlet-l,...) and should unify these notations.

We have fixed the labelling in Fig. 3E and have consolidated the nomenclature throughout the manuscript, by using mScarlet-I throughout.

4) First sentence of the chapter 'A coupled Hes1-Hes5 oscilation model...' on pg. 9 requires a reference to a figure. Similarly, the statements in the last sentences of the first paragraph on pg. 10 ('When the Hes5 level...'until the end of the paragraph) should be supported by a figure

© 2026. Published by The Company of Biologists under the terms of the Creative Commons Attribution License (https://creativecommons.org/licenses/by/4.0/).

reference.

We have introduced figure references in both locations on page 9 and 10.

5) Top of Pg. 11: The authors state that they see occasional transitioning from fast to slow HES1 oscillations as a consequence of HES5 upregulation in some pri-NPCs (Fig. 4F). It would be nice if the authors could provide a quantification for how often this phenomenon occurs.

In our priNPC data, transitions in HES levels are rare, observed only in 3/18 tracks. In these rare cases, we see that the changes in HES5 levels is associated with changes in HES1 oscillation period, reported on page 8. However, this frequency of transitioning is likely underestimated because most cells could only be tracked for relatively short durations (on average 10hrs and only 4% of tracks are >15 hrs), limiting our ability to capture such transitions.

6) Pg 14: 'important insights into how the two Hes oscillators interact in a single cell'. Because Hes proteins can form homo- and heterodimers, 'interact' may be misleading here.

We avoided the use of the word ‘interact’ in favour of ‘HES oscillators **regulate each** other’ throughout the revised manuscript.

7) In the section 'Establishment and maintenance of primary neural progenitor cultures' in the Materials and Methods, the authors refer to dissecting LGEs of e13.5 mouse embryos, which they have not done in this version of the manuscript.

We apologise for any confusion, but we did use in vitro data obtained from priNPCs dissected from E13.5 LGE mouse embryos. These cells contained the two HES1 alleles tagged with different fluorophores (new **Fig. S2**) and were used these to demonstrate that the observed differences in HES1 and HES5 peak-to-trough fold change are not attributable to the different fluorophores used for tagging (mVenus vs mScarlet-I). We amended the text on page 6 to better flag that the HES1::mVenus is a newly reported mouse line.

8) The authors provide validation for the entrainment of HES1 by HES5 in the manuscript by showing that the period of the HES1 oscillations is significantly shorter in the lowest HES5 quartile group (bottom of pg. 10) and that they see occasional transitioning from fast to slow HES1 oscillations as HES5 is upregulated in some pri-NPCs (top of pg 11). While these observations support their model, it would be great if the authors could provide a more direct validation. This could be for example done by demonstrating that cells acquire faster HES1 oscillations in ES-NPCs differentiated from HES5 knock-out cells, while HES5 oscillation period should not be affected in HES1 knock-outs.

To directly test the entrainment predictions while avoiding earlier developmental effects of Hes1 loss, we used siRNA-mediated knockdown specifically at the NPC stage. We then measured both expression levels and oscillation periods of HES1 and HES5 following knockdown of the alternate Hes gene. Consistent with the model prediction of Hes1/Hes5 cross-repression, we indeed observe higher levels of HES5 and HES1 protein than the control when the alternate HES is perturbed (i.e. siRNA of Hes1 or Hes5 respectively) (**Rebuttal Fig 3, new Fig 4F**).

© 2026. Published by The Company of Biologists under the terms of the Creative Commons Attribution License (https://creativecommons.org/licenses/by/4.0/).

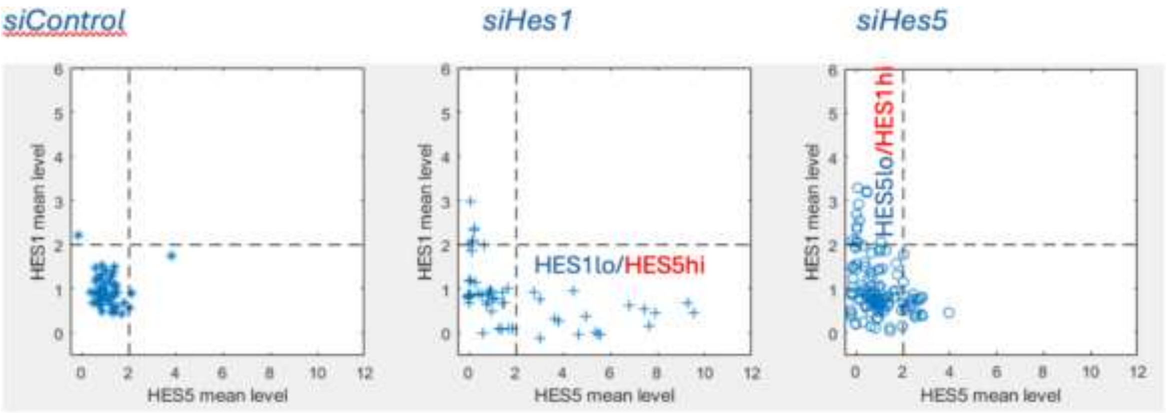

Rebuttal Fig 3. Comparison of mean level in control (non-targeting siRNA, siControl), *Hes1* knock-down (*siHes1*) and *Hes5* knock-down (*siHes5*) (**new Fig 4F**).

However, we observed either no change or a slight increase in oscillation period in single HES expressing cells relative to double-positive cells (**Rebuttal Fig 4, new Fig 4G**). Thus, although our correlative observations (now found in **Fig 4B**) suggest a shorter HES1 period in HES5-low cells compared to HES5-high cells and our mathematical model predicts entrainment, this new perturbation analysis did not support a scenario in which HES5 entrains HES1 oscillations directly. Accordingly, we have moderated the wording in the manuscript to avoid implying direct entrainment.

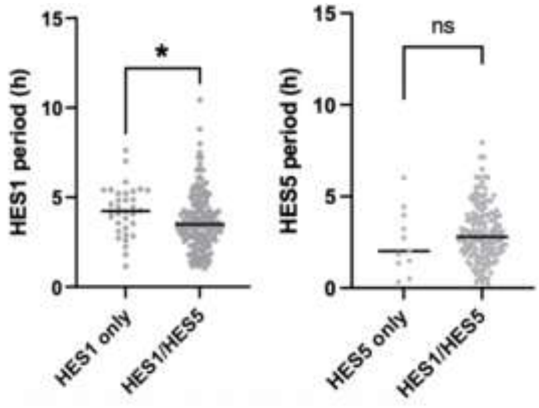

Rebuttal Fig 4. Period values detected in single HES1 and single HES5 oscillatory cells from the siRNA data (**new Fig 4G**).

Alternatively, the authors could check if they, as in the embryonic spinal cord, observe more frequent transitions from the HES1-HES5 hybrid state to a HES1 single state, if they analyse the dorsal ES-NPC differentiations at a slightly later stage of the differentiation protocol, and if this increased rate of transition is associated with an increased oscillation frequency.

In **new Fig 6C** of the revised manuscript, we have included an analysis of later stages of the dorsal NPC differentiation protocol (**Rebuttal Fig 5**). This analysis shows a decrease in the proportion of HES1+/HES5+ double-positive cells accompanied by a corresponding increase in cells expressing only a single HES protein at later stages, supporting the in-vivo evidence of transitions from an initial HES1-HES5 hybrid state to a single HES state.

© 2026. Published by The Company of Biologists under the terms of the Creative Commons Attribution License (https://creativecommons.org/licenses/by/4.0/).

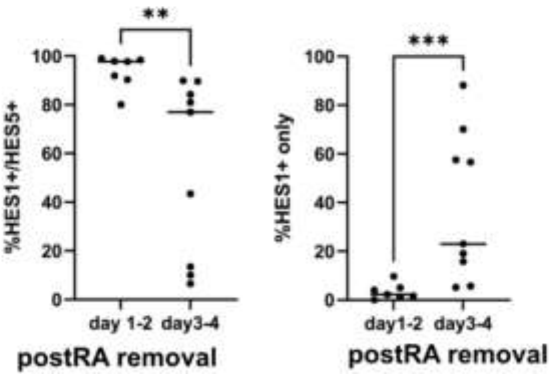

Rebuttal Fig 5. Percentages of live mES- derived NPCs that co-express or single express compared at different stages in the in vitro differentiation (**new Fig 6C**).

Reviewer 2: SUMMARY OF THE ADVANCE MADE IN THIS PAPER AND ITS POTENTIAL SIGNIFICANCE TO THE FIELD

Biga et al. address in their manuscript "Oscillatory Co-expression of Hes1 and Hes5 enables a hybrid state in a bistable transcription factor regulatory motif" how multiple Hes transcriptional repressors interact and influence each other in mouse neural progenitors. Hes proteins are dynamic in many cell types due to their negative autoregulation and co-expression is a common feature. How the parameters of each delayed negative feedback loop affect each other due to cross-repression has not been addressed yet. Here, the authors quantified Hes1 and Hes5 dynamics in the developing neural tube both in primary cell culture and in vitro differentiated cells. They then use a theoretical model to explore the parameter space and determine when and how one TF "wins". They then correlate this finding to quantifications in cells. This paper is very relevant for neural tube development and other processes containing multiple oscillatory systems, e.g. the segmentation clock.

SUGGESTIONS TO AUTHORS

There are a few points that would be worth addressing before publication:

Major points:
- The manuscript deals with the Hes1-Hes5 network as isolated system. The authors should at least mention that other factors such as signalling, other TFs, epigenetic changes etc. probably have an influence and will probably modulate the oscillation period as well.

We agree that other factors such as the ones mentioned by the reviewer may have a role in modulating HES1-HES5 oscillations and we have included this as a new discussion point on page 14: “Although our study focuses on the intrinsic dynamics of the HES1- HES5 network, in vivo this circuit is embedded within a broader regulatory context. Morphogen signalling pathways, additional bHLH transcription factors (e.g OLIG2) and chromatin or epigenetic regulation may influence the dynamics of the network, potentially modulating oscillatory properties”.

Something the authors mention in the result section: "These observations are consistent with the fact that the mES differentiation protocol enriches for dorsal types (Gupta et al., 2022) (identified by expression of PAX3/7) where HES1 is more widely expressed compared to ventral (Sagner et al., 2018); in contrast, the priNPCs could be ventral or dorsal, with HES5 being expressed in both areas (Manning et al., 2019)."

We have included a schematic diagram of HES1 and HES5 expression in relation to the D-V axis to clarify the D-V position of expression of each gene (**new Fig 7G**).

In the same regard, a discussion of how the parameters change in the context of neural differentiation should be added. Why does the cross-repression threshold change, as cells differentiate, which allows the stabilization of one TF over the other. They mention in the results that "experimental evidence suggests parameters can change (...) following cell division". In the

© 2026. Published by The Company of Biologists under the terms of the Creative Commons Attribution License (https://creativecommons.org/licenses/by/4.0/).

discussion, the authors should discuss and speculate what the mechanism for changing parameters and thereby changing periods etc is. This would then also link back to the process of neural tube development, which is only superficially addressed in the discussion now.

We agree that the Hes1-Hes5 motif is unlikely to be an isolated system. We have added a more detailed discussion of how changes occurring during development may affect the cross-repression and oscillatory dynamics during differentiation. On page 15: “In vivo, such resolution could be mediated by region-specific negative regulation by TFs OLIG2 and HES6 during spinal cord neurogenesis (Sagner et al., 2018, Fior et al., 2005). It may also involve additional molecular mechanisms proposed for somitogenesis, such as modulation of degradation rates and strength of DNA binding activity through Hes/Her dimerization (Schroter et al., 2012) as well as increasing gradients of transcriptional delay in Her1 (Ay et al., 2014). “

- In the introduction, the authors "present the challenge" of co-existence of cross- repressive TFs and fate determinants. Something that is observed in many differentiation processes for various TFs. What is special about Hes proteins is that they are not only cross-repressive but also auto-repressive. This makes their dynamics different from other cross-repressive TF pairs. This is an aspect the authors should highlight and could also address in their discussion. e.g. What do we learn from this paper for other non-autorepressive TF pairs?

We have expanded our discussion to highlight the fact that non-autorepressive TFs can also oscillate, On page 13:

“While HES1 and HES5 oscillate by a combination of auto-repression, protein and mRNA instability and appropriate time delays, other motifs such as non- autorepressive TFs (Bokes and King, 2019, Guantes and Poyatos, 2008) or the AC/DC motif of 3TFs, are also known to generate hybrid oscillatory states in silico (Perez-Carrasco et al., 2018). Therefore, we expect our findings to be more widely applicable.”

- In Figure 3, signal from both Hes1 and Hes5 are quantified in the same cells and phase-phase maps are generated. It would be interesting to know what the heterogeneity between cells is.

It is stated that there is a "presence of stochasticity". Is this because oscillations are in- phase in some cells and not in others or is it because it is variable within single cells? Do variations correlate with Hes1 or Hes5 levels or differentiation state? This data should be present in their data.

In the revised manuscript, we provided examples of phase- phase heterogeneity showing phase relationships can vary between cells, and also in the same cell over time (**new Fig S3D**). In addition, we report that 80% of co-expressing cells show in-phase HES1-HES5 oscillations in the same cell, quantified using two criteria (see updated Materials and methods, **Rebuttal Fig 6, new Fig S3E**).

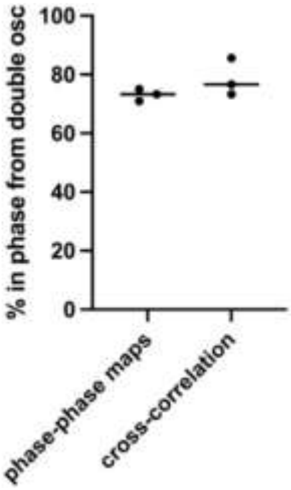

Rebuttal Fig 6. Percentage in-phase per experiment (**new Fig S3E**).

- In Figure 4, the authors discuss how the presence of one TF influences the oscillations of the

© 2026. Published by The Company of Biologists under the terms of the Creative Commons Attribution License (https://creativecommons.org/licenses/by/4.0/).

other. Using a coupled-oscillator model, they conclude that the "slow oscillator Hes5 cannot speed up", which is why they only test the effect of Hes5 levels on Hes1 (and Hes5) oscillation period. They should also check the other way around. As they have Hes5+/Hes1- cells in their dataset, they could check how the presence of Hes1 influences Hes5 oscillations. It is still possible that Hes1 also entrains Hes5 and leads to a slight decrease in the Hes5 oscillation period. Considering that Hes5 is also oscillatory in mouse somitogenesis with a general period of 2.5 hours, a possible decrease in the Hes5 oscillation period is in likely.

We thank the reviewer for the opportunity to provide a more balanced exploration of bi- directional entrainment. In the **new Fig 4C,D** (**Rebuttal Fig 7**) we have included side by side quartile data of period of either HES versus the level of the other, with the 0-25% quartile indicating free-running HES while the 25-100% quartiles showing period convergence. This data is now discussed in the new results section “The periodicities of HES1 and HES5 converge when they are co-expressed”. We have also provided a more detailed exploration of the model showing that bidirectional entrainment is possible, found in **new Fig S5B.**

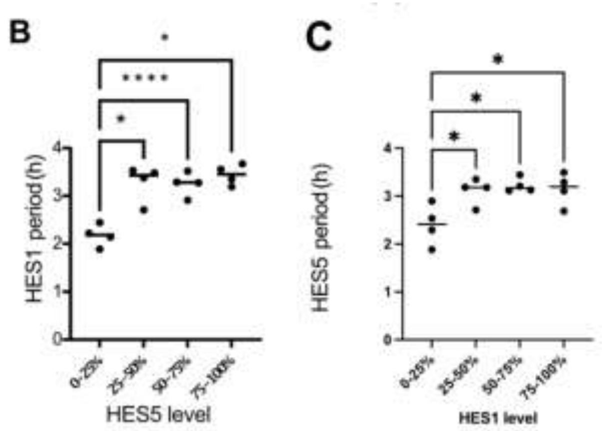

Rebuttal Fig 7. Binning of period of HES1 and HES5 by the mean level per track of opposite HES (**amended Fig 4B,C**).

In addition, we have performed siRNA experiments to knockdown one HES and measure the level and oscillatory period of the other HES (**Rebuttal Fig 8, new Fig 4F,G**). The analysis of expression level in perturbed conditions demonstrated that the cross- repressive motif is bidirectional, however the period of one HES did not reduce when the other is knocked down. Therefore, we have moderated our conclusions with regards to entrainment found on page 10: “while there is a correlation between co-oscillation and change in each HES’s individual periodicity, the effect is unlikely to be due to simple or direct entrainment.”

© 2026. Published by The Company of Biologists under the terms of the Creative Commons Attribution License (https://creativecommons.org/licenses/by/4.0/).

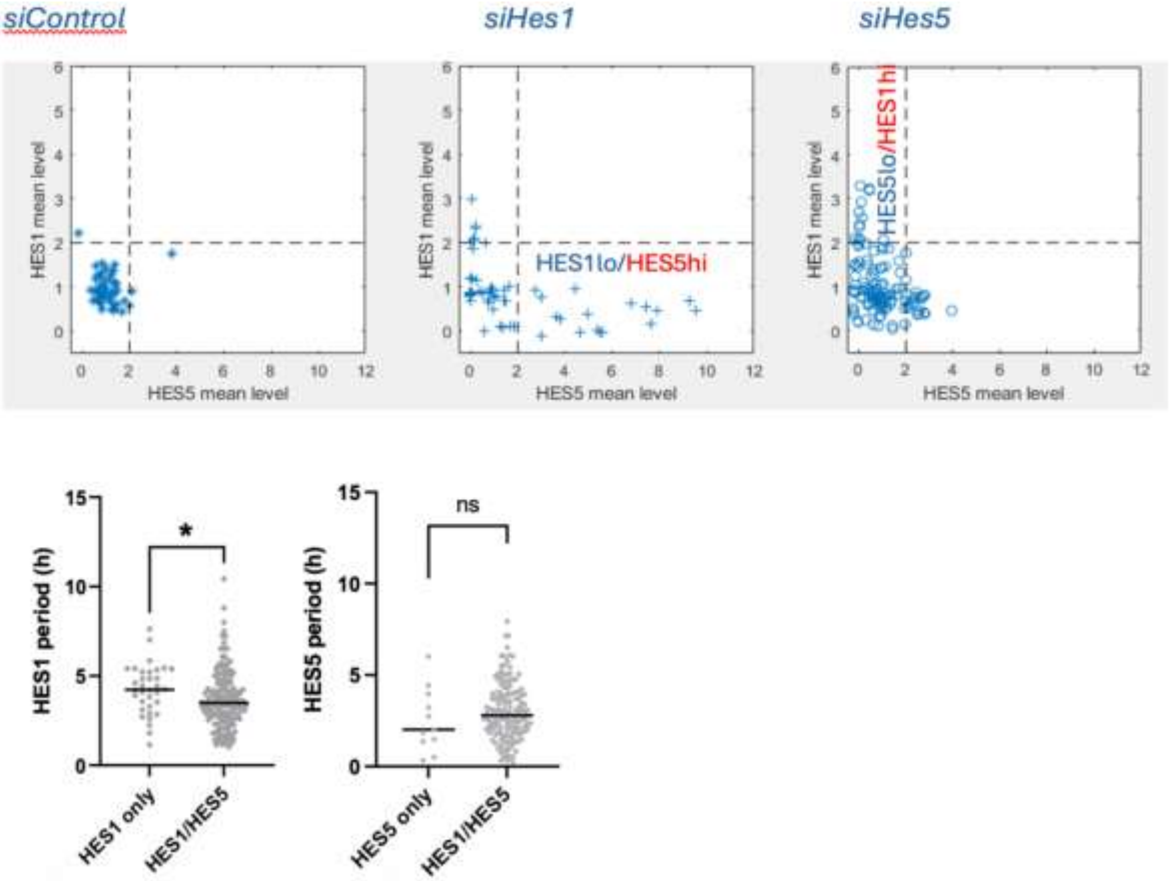

Rebuttal Fig 8. (top) Comparison of mean level in control (non-targeting siRNA, siControl), Hes1 knock-down (siHes1) and Hes5 knock-down (siHes5) (**new Fig 4F**).

(bottom) Period values detected in single HES1 and single HES5 oscillatory cells from the siRNA data (**new Fig 4G**).

- Figure 1B, C: Cell density in mESC-derived NPCs is higher than for primary NPCs. Are these images indeed representative? Does cell density explain slight differences in oscillation parameters? An easy way to address this would be to quantify dynamics in less dense regions.

Yes, these images are representative. Identifying less dense regions is diGicult in both systems because cells form extensive contacts: primary NPCs are highly motile and form transient network-like contacts (see **Movie 1**), while mES-derived NPCs organise into pseudostratified rosettes with interkinetic nuclear migration (see **Movie 2**). Although mESC-derived NPCs appear denser due to rosette formation, comparison of the two systems revealed no significant differences in the oscillation period of HES1 or HES5 (**Rebuttal Fig 9; new Fig. 2J**), indicating that cell density does not measurably affect the oscillatory dynamics.

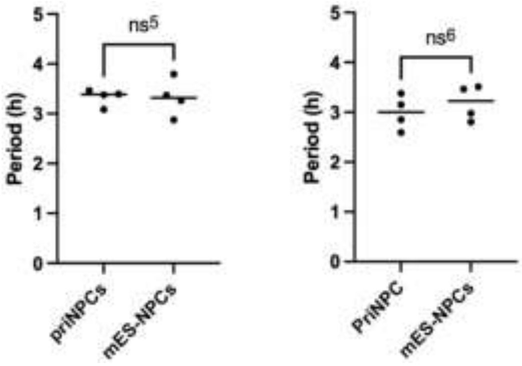

© 2026. Published by The Company of Biologists under the terms of the Creative Commons Attribution License (https://creativecommons.org/licenses/by/4.0/).

Rebuttal Fig 9. Median period values per experiment compared in the two culture systems (**new Fig 4J**).

- Hes5 Hes1 reporter mice were used heterozygously, while mESC-derived NPCs were homozygous. The authors could clarify what the phenotype of double hom mice is? Is there any diGerence in period?

The double HES1/HES5 homozygous mice are viable with mendelian ratios and we included a statement in Materials and methods on page 16. We tested that the HES5 period is unchanged in homozygous versus heterozygous priNPC spinal cord cultures containing the Venus::HES5 reporter (**Rebuttal Fig 10, new Fig S2G**).

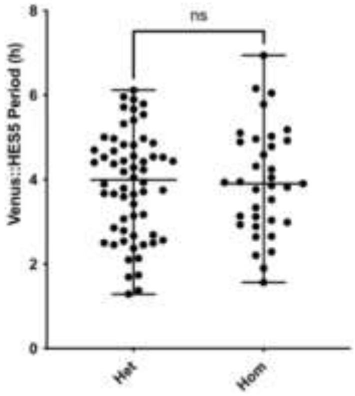

Rebuttal Fig 10. Comparison of period in hom vs het priNPC Venus::HES5 cultures (**new Supp Fig 2G**).
For HES1, we have the equivalent to homozygous where each allele is labelled with a different fluorophore, reported in **Fig S2**. The two copies of HES1 are highly synchronous and the period values (observed with either fluorophore) are within the expected ultradian range and unchanged between the 2 alleles (**Rebuttal Figure 11, new Fig S2D,E**). On page 6: "These observations argued against artefactual differences due to the choice of fluorophores, mono-allelic versus bi-allelic expression and confirmed that HES1 is more oscillatory and has a higher fold-change compared to HES5".

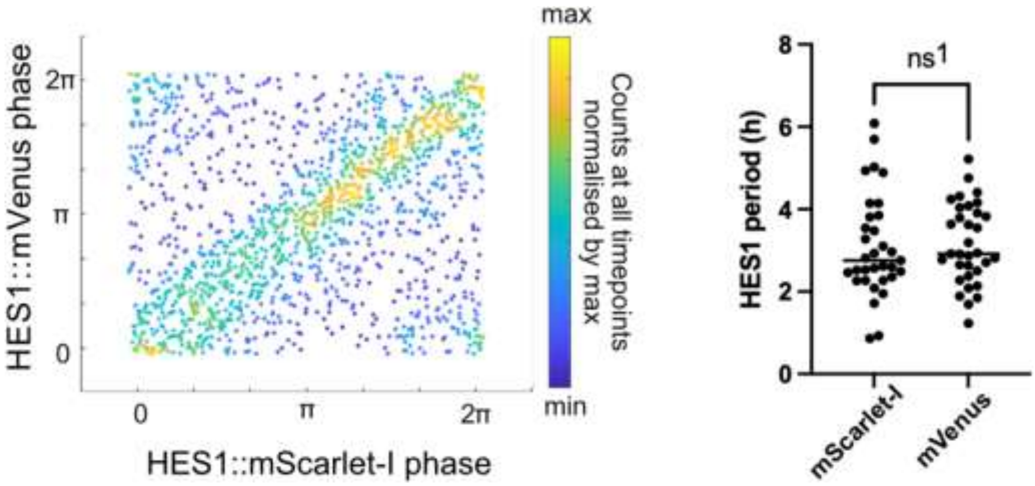

Rebuttal Fig 11. Phase-phase mapping and period measurements in double HES1 reporter priNPC cultures (new Fig S2D,E).

Minor points:
- In the introduction, the authors discuss the presence of hybrid states identified in scRNAseq experiments. However, protein is not always the same as RNA. Maybe phrase more carefully?

Yes, we agree and have adjusted the wording on page 3 in the revised manuscript.

- The authors quantify the peak-to-trough fold-change, which is a measure of the oscillation

© 2026. Published by The Company of Biologists under the terms of the Creative Commons Attribution License (https://creativecommons.org/licenses/by/4.0/).

amplitude. They also Hilbert transform to determine the oscillation phase. They could use the same approach to determine the (instantaneous) amplitude (and period), which they could then link to Hes1/ Hes5 expression levels over time (as in Figure 4F).

As suggested, in the revised manuscript we have included correlation tests of period versus level of either HES (**new Fig S4D**) and fold-change versus level of either HES (**new Fig S7B**) but found no significant relationships between these variables either as mean per track or in terms of instantaneous values, see **Rebuttal Fig 12.**

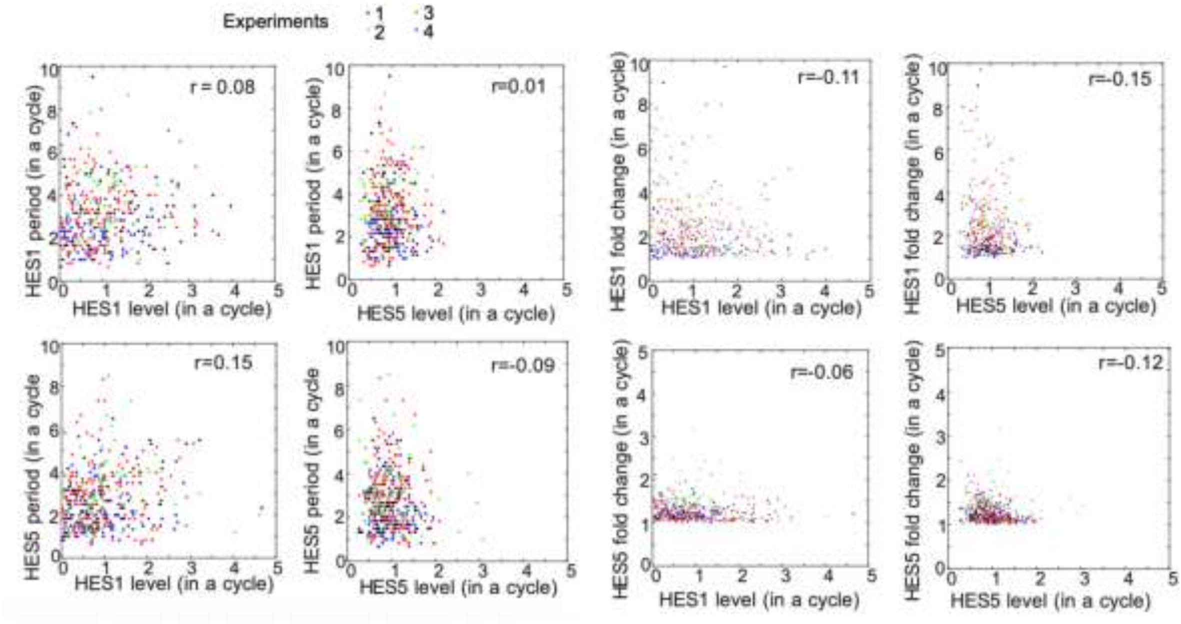

Rebuttal Fig 12. Correlation coeGicient analysis of instantaneous period and peak-to- trough fold change versus mean level of either HES in each cycle (included in new **Fig S4E, Fig S7C**).

- Results part: "Like priNPCs, in mES-derived NPCs HES1 oscillations were more - frequent, and ". What is meant with more frequent? Oscillations occurred more frequently or the frequency was higher? It might be good to re-phrase to prevent confusion.

By ‘more frequent’, we intended to say ‘occuring more frequently’. We have re-phrased the text in the revised manuscript on page 7.

- I don't see a section on period quantification in the method section. This should be added.

We have added a section on period quantification in the Methods of the revised manuscript on page 20.

- Figure 6: Consider adding a scheme where along the DV axis of the neural tube Hes5 and Hes1 are expressed (maybe even over development).

We have added a schematic of expression across the DV axis of the neural tube (**Rebuttal Figure 13, new Fig 7G**).

© 2026. Published by The Company of Biologists under the terms of the Creative Commons Attribution License (https://creativecommons.org/licenses/by/4.0/).

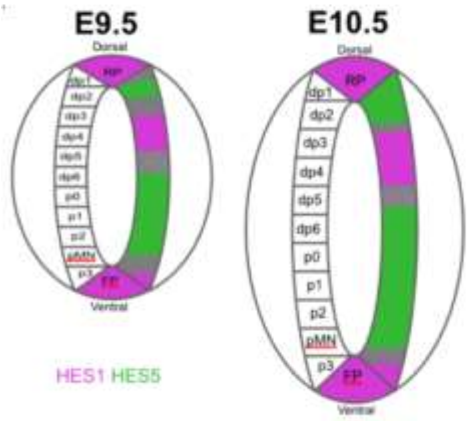

Rebuttal Fig 13. Schematic representation of HES1/HES5 over development (included in **new Fig7G**).

- Typo in Methods: "514nm laser (Venus). was de Z-sections covering the"

We also thank the reviewer for all the additional suggestions, which have now been included in the amended manuscript.

Other point:
The model based on a coupled oscillator model makes sense to me. However, I am not judging this from a physicist's point of view.

Reviewer 3: SUMMARY OF THE ADVANCE MADE IN THIS PAPER AND ITS POTENTIAL SIGNIFICANCE TO THE FIELD

In this manuscript, the authors address a longstanding conundrum: how cross- repressive transcription factors such as Hes1 and Hes5, known to encode distinct downstream cell fates, can coexist dynamically within single cells in the developing neural tube. Specifically, they show that oscillatory expression allows these factors to be co-expressed in a temporally coordinated fashion, effectively delaying cell fate resolution and enabling hybrid states. The authors support this hypothesis through a combination of live-cell imaging and a mathematical model grounded in biophysical parameters. The model captures both known and emergent behaviours, including oscillator entrainment and how variations in interaction strength and degradation rates affect cell fate outcomes.

Overall, the study is solid and provides very interesting insights into temporal regulation of cell fate. However, several conceptual and technical aspects could benefit from clarification or deeper analysis, particularly regarding the definition of cell states and the interpretation of oscillatory dynamics.

SUGGESTIONS TO AUTHORS

Major Comments
1) The manuscript would benefit from clarifying how the authors define cellular states, especially in the abstract and discussion. Are the authors proposing three discrete dynamical regimes (e.g., two stable states and one hybrid), or is the oscillatory state a transient phase (e.g., a damped or spiral attractor) en route to one of the stable states? This distinction has important implications for how we conceptualise cell states in development, which are rarely truly stationary.

We thank the reviewer for the opportunity to clarify that our data supports the existence of 3 distinct states including the co-oscillatory/hybrid state and 2 single oscillatory ones which are abundant in vitro however may be transient in vivo. If the hybrid state was a transient state, e.g. a spiral attractor, we would expect a dampening of the amplitude of HES oscillations over time. In **Rebuttal Fig 14** (**new Fig 6B**) we show that oscillations persist for many cycles in the same cell. We also provide additional quantification indicating that double HES and single HES fractions are observed in progenitors over many differentiation days (**new Fig S1B**). Taken together, these observations argue against damping.

© 2026. Published by The Company of Biologists under the terms of the Creative Commons Attribution License (https://creativecommons.org/licenses/by/4.0/).

We have re-written the abstract and amended the discussion on page 13: "Our work expands our understanding of transitions in development in many ways. First, our data supports the existence of many more attractors than currently appreciated. Our study suggests that oscillatory dynamics of cross-repressing (and auto-repressing) TFs permit the existence of a hybrid state that would otherwise be expected to be transient or inaccessible. Second, the resolution of the HES1-HES5 motif involves two additional attractors each representing the single oscillatory HES, before it transitions further into a state where HES expression is extinguished as cells differentiate. "

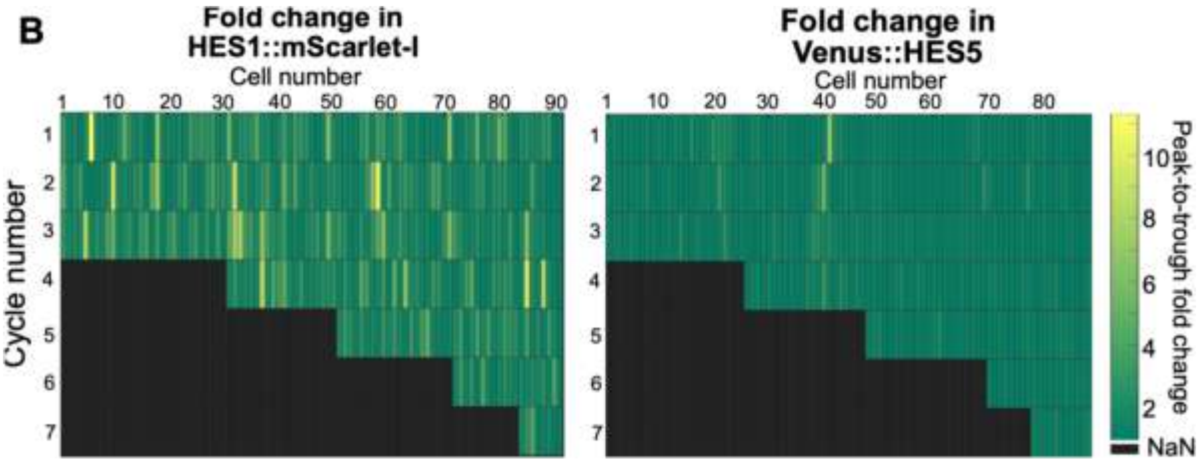

Rebuttal Figure 14. Fold change values in consecutive cycles observed in HES1 and HES5 (**new Fig. 6B**).

The same apply for the mathematical model, is there any stable oscillation state observed, or there is always a single attractor in the system?

In the revised manuscript, we have added model exploration of the two single HES and the hybrid state and identified areas of parameter space that support the existence of sustained oscillatory activity as opposed to damped oscillations for all 3 states. Sustained co-oscillations occur at high nonlinearity in the auto-repressive Hill (**Fig S4A**) which is consistent with previous Bayesian inference estimates placing HES1 and HES5 data at Hill coeGicient values from 5 to 15 (Heron et al., 2007, Burton et al., 2021).

As our study reveals an exciting new type of cell state transition from double co- oscillating to single oscillating regimes, a full exploration of attractors in the HES1-HES5 motif will be followed up in a subsequent publication.

2) If I understood correctly, trends are removed before analysing oscillatory characteristics. However, these trends themselves may encode important biological information. For instance, changes in the mean levels of HES proteins could reflect cell state transitions. In many traces (e.g., HES1 in Fig. 3A), changes in the trend exceed the oscillation amplitude. Have the authors analysed these trends separately? Are they correlated with period or cell decision?

In the analysis of periodicity, we detrend the data, as required for frequency-based analysis. However, the peak to trough fold-change measure represents the raw/total intensity level at the peak versus at the trough (now signposted in Materials and methods page 20). In the revised manuscript, we correlated period and fold-change versus level (which reflects any fluctuations in trends). These correlations were non- significant most likely due to low probability of transitions from double HES to single HES states in our data (**Rebuttal Fig 15, new Fig S4D, new Fig S7C**).

© 2026. Published by The Company of Biologists under the terms of the Creative Commons Attribution License (https://creativecommons.org/licenses/by/4.0/).

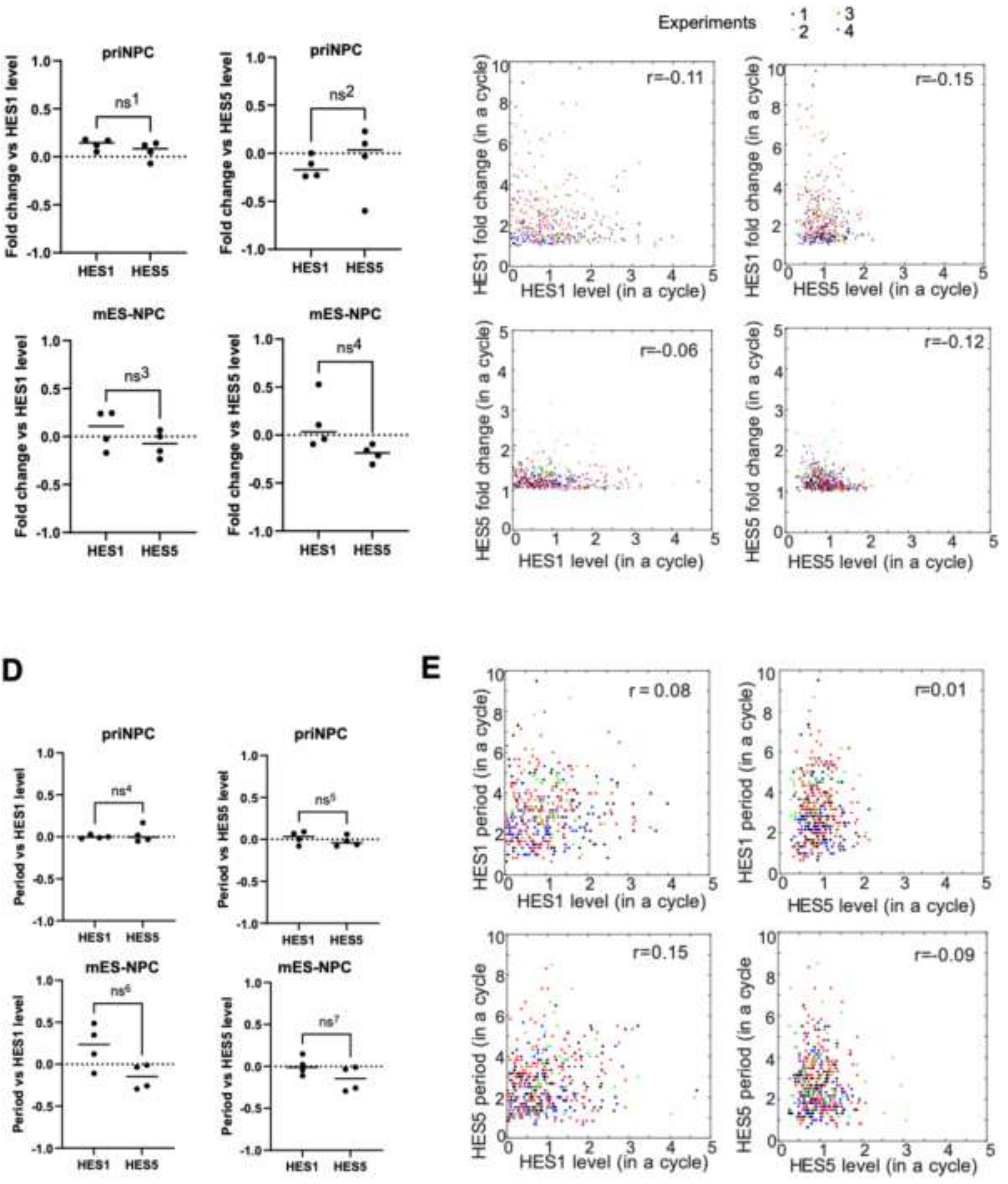

Rebuttal Fig 15. Correlation analysis of period and fold-change parameters versus HES level measured as average per track (right panels) and as instantaneous (per cycle-left panels); included in **new Fig S7C-D, Fig S4D-E**.

Are they (the trends) captured by the mathematical model?

In the new section 'Limitations of the study', we have stated that slow-varying fluctuations (or trends) in level of expression are not accounted for by the model (page 15).

3) The model is interesting, and is simple enough to gain mechanistic understanding of the emerging behaviours, but could be explored in greater depth:
3a) The initial large peak in protein levels appears to result from initial conditions (zero protein). How sensitive are the dynamics to this choice, and how do they compare to experimental starting

© 2026. Published by The Company of Biologists under the terms of the Creative Commons Attribution License (https://creativecommons.org/licenses/by/4.0/).

conditions?

In the revised manuscript, we have varied the initial conditions in terms of the starting protein/mRNA levels and we observe how following the initial cycle, the output is in- phase, with some variation between subsequent cycles (**Rebuttal Fig 16, new Fig S5A**). Determining the experimental starting conditions would require extensive protein and mRNA absolute quantitation experiments, however in the absence, we have provided examples of phase over time in the experimental data (**new Fig. S3D**). Overall there is agreement between model and data.

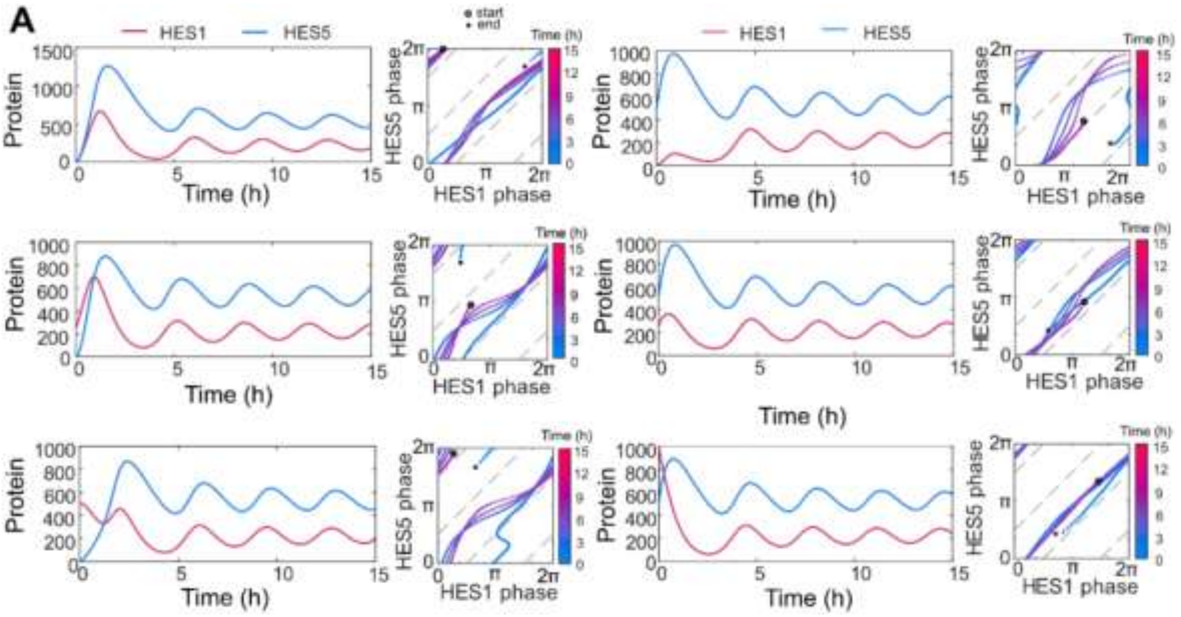

Rebuttal Fig 16. Model simulations for different initial conditions with corresponding phase-phase mapping over time (**new Fig S5A**).

3b) Does the model predict timescales for oscillation damping? Can these be compared quantitatively to the experimental data?

Depending on parameters, the model reproduces either persistent or damped oscillatory states, however our experimental data argued against damping as discussed in point 1.

3c) Phase relationships in Fig. 3F show apparent synchrony, but in Fig. 4B, a phase lag of ~π/2 is observed. Can the authors explain this discrepancy? Phase diagrams for the model across different parameter regimes would help clarify when and how in-phase locking emerges.

The model explorations included in response to point 3a indicate that, depending on initial conditions, a phase shift of approx. π/2 can be observed in the first cycle, however in subsequent cycles this is reduced and largely remains in the in-phase region (**Rebuttal Fig 16, new Fig S5A**).

4) The observation that HES1 period increases in the presence of HES5 is compelling. However, since HES1 and HES5 levels are positively correlated, could the change in period be simply a function of HES1 expression levels? Would plotting HES1 period against HES1 amplitude alone in Fig 4D (rather than against HES5 levels) show a similar trend?

© 2026. Published by The Company of Biologists under the terms of the Creative Commons Attribution License (https://creativecommons.org/licenses/by/4.0/).

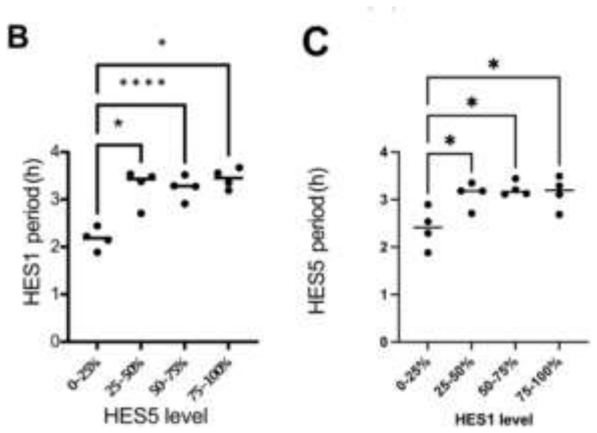

Indeed, the cells with the lowest HES1 level also have decreased HES1 periods (**new Fig S4C**). The period values are weakly correlated to fold-change in the same HES (HES1: r=0.36; HES5: r=0.37). As such, in the case of either HES when they are co-expressed, i.e. quartiles 25-100% they adopt the same period which is different from the free-running period in the 0-25% quartile.

Overall, we share the reviewer's concern that the evidence we provided is correlative with multiple confounding factors. In the revised manuscript, we have performed new experiments to establish if the period of HES1 changes in the absence of HES5.

We tested for bidirectional effects in the HES1-HES1 regulatory motif by knocking-down one *Hes* and monitoring the effects on the protein of the other. Our experimental results indicate that as expected, high HES5 expressing cells are observed when Hes1 is knocked-down and conversely, high expressing *HES1* cells are observed when Hes5 is knocked-down confirming bidirectionality. The perturbed cells that are generated in either condition, single HES1 or single HES5 continue to oscillate however the period of HES1 is not reduced. As a result, we have moderated our conclusions on entrainment, found on page 10.

© 2026. Published by The Company of Biologists under the terms of the Creative Commons Attribution License (https://creativecommons.org/licenses/by/4.0/).

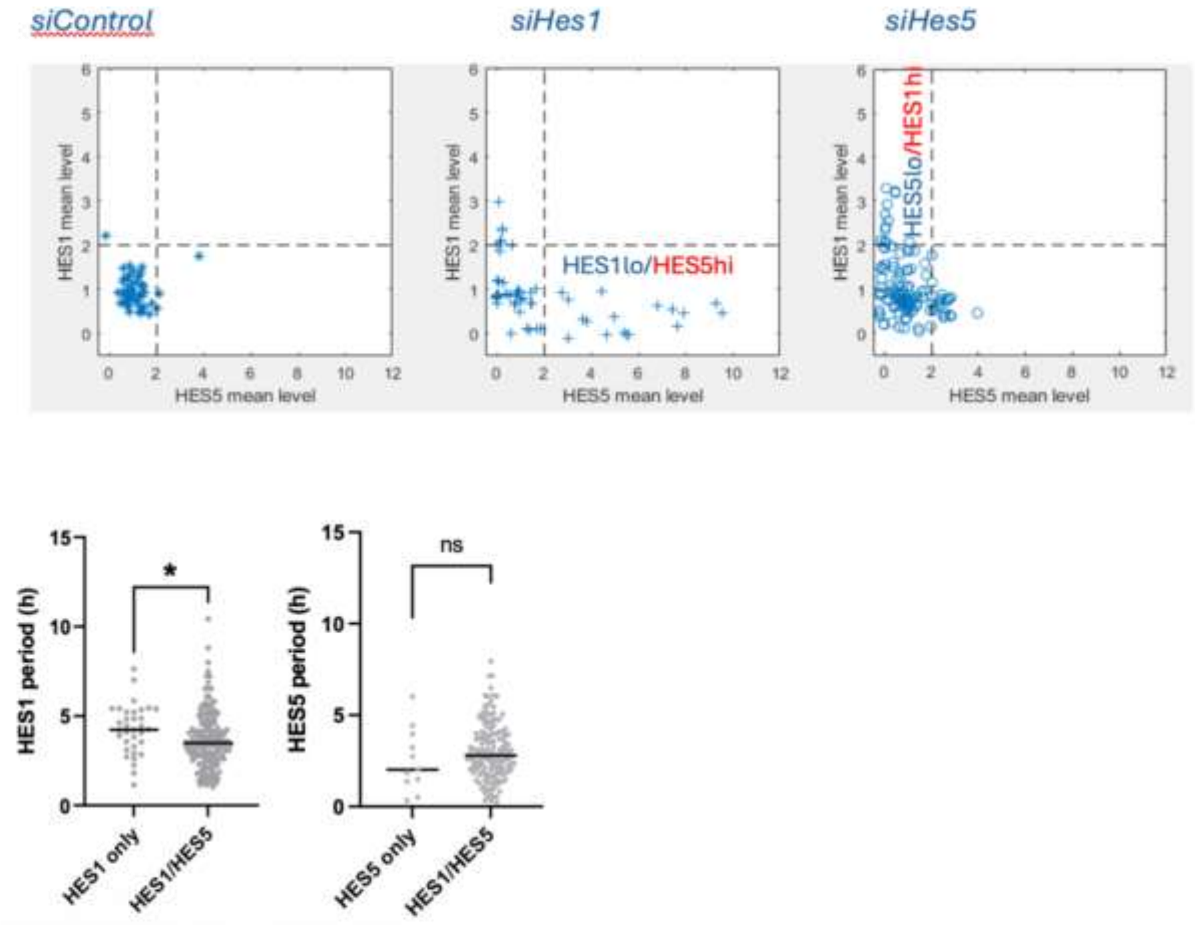

Rebuttal Fig 17. (top) Comparison of mean level in control (non-targeting siRNA, siControl), Hes1 knock-down (siHes1) and Hes5 knock-down (siHes5) (**new Fig 4F**).

(bottom) Period values detected in single HES1 and single HES5 oscillatory cells from the siRNA data (**new Fig 4G**).

5) The manuscript refers to fate decisions at the point of cell division. If this refers to a specific hypothesis regarding levels or phases of HES oscillations, a statistical analysis addressing this directly would help clarify the claim

A possible hypothesis is that cell division provides a point for transitions between the hybrid state and single HES oscillatory state. In the revised manuscript we analysed the variability of the trend of either HES in the mother versus daughter cells and identified that 5/24 divisions are divergent with one of the daughters (but not the other) showing a higher coeGicient of variation compared to any other mother cell (Fig. S7B) reported on page 11. While we agree that this is an interesting observation, the sample size is too low to carry out further analysis.

6) The model shows that increasing repression thresholds or mRNA production can push the system out of the oscillatory regime toward dominance by a single TF. This is interesting, but likely not the only possible mechanism. For instance, changes in degradation rates (potentially through post-translational modifications or heterodimer formation) or delays in transcriptional feedback (the delay parameter imposed in the differential equaion) could achieve similar transitions. Can the authors comment on the specificity of the mechanisms they identify, given the available data?

In the revised manuscript, we have carried out additional exploration of the parameters that can contribute to the resolution towards dominance by a single HES (**new Fig 4C, new Fig S6C**). We find that while protein stability affects the area under the curve (AUC) of the other HES, the combination of cross-repression threshold and/or production rate increase is effective at any half-life. Time delay however does not have an impact. Overall, we observe that cross-repression threshold is most effective at

© 2026. Published by The Company of Biologists under the terms of the Creative Commons Attribution License (https://creativecommons.org/licenses/by/4.0/).

suppressing AUC of the other HES; this effect can be enhanced by increased production rates. We have also provided a discussion of additional mechanisms that could contribute in vivo on page 13.

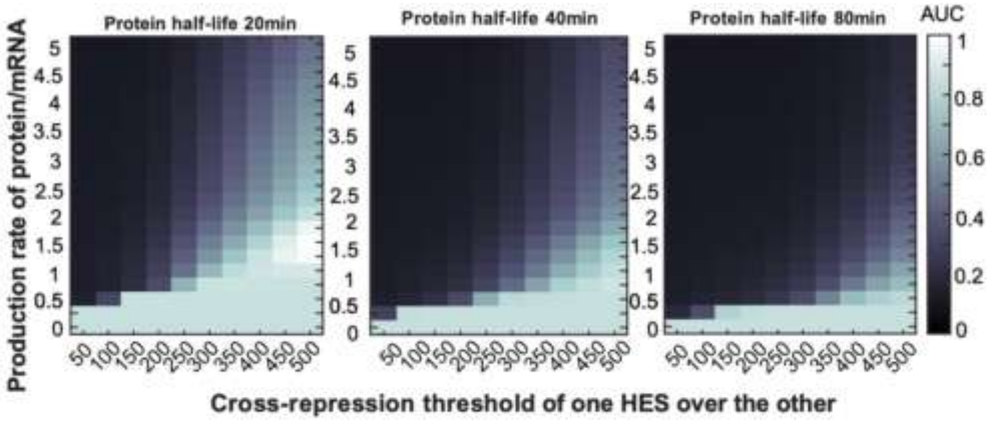

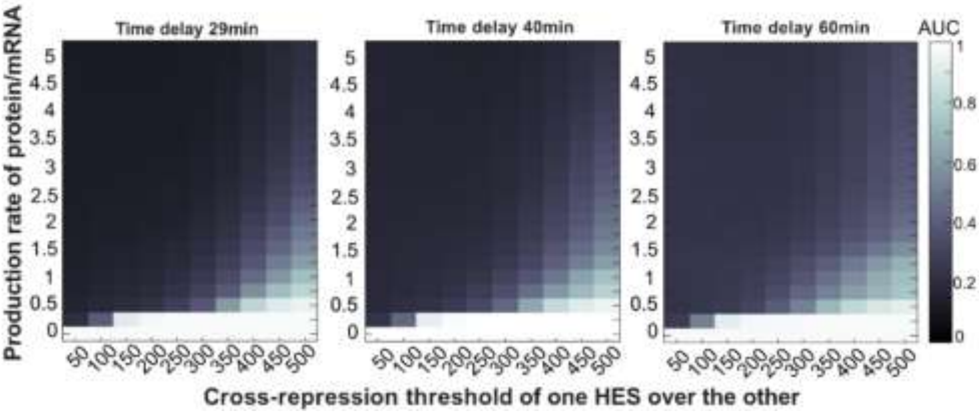

Rebuttal Fig 18. Mapping of area under the curve for one HES when the parameters of the other HES are varied to increase cross-repression.

Minor Comments

1. In the discussion of intermediate states, the authors could reference examples of bistable circuits that exhibit a third, stable co-expressing state via self-activation, such as Guantes & Poyatos (PLoS Comp Biol 2008, doi:10.1371/journal.pcbi.1000235), as well as synthetic gene circuit implementations of such circuits (Wu et al. eLife 2017;6:e23702. DOI: 10.7554/eLife.23702).

We have included these citations on page 3.

2. The identification of "dominant" cells in the experimental data could be more clearly explained. This also links back to the definition of cell states (see Major Comment 1).

We defined this in the manuscript on page 7: "corresponding to one HES oscillating while the other is very low, a regime that we refer to as dominant".

3. The methodology for oscillation detection could be described more precisely:
3a) Statements such as "data was de-trended using a 7.5 lengthscale parameter" are too vague for reproducibility.

We apologise for the lack of clarity. We have added the relevant methodological details on page 20.

3b) The use of Hilbert transforms for phase detection could be complemented by more intuitive

© 2026. Published by The Company of Biologists under the terms of the Creative Commons Attribution License (https://creativecommons.org/licenses/by/4.0/).

analyses, such as computing time-lagged correlations (e.g., $\langle H1(t)H5(t-\tau)\rangle$ as a function of τ). This would give direct insight into phase relationships and how they evolve over time.

In the revised manuscript, we provided examples to show that phase relationships do vary both over time in the same cell and also between cells (**Fig. S3D**). We have also used both phase- phase maps and cross-correlation analysis in the same track (described in Materials and methods on page 21) to determine that approx. 80% of cells have in-phase HES1 and HES5 (**Rebuttal Fig 18; new Fig S3E**).

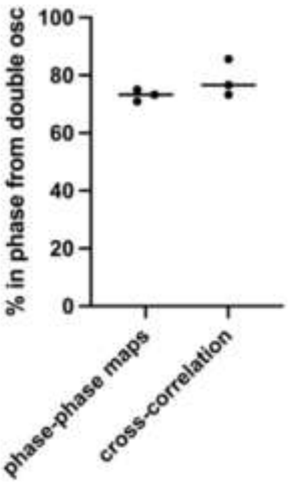

4. The sentence "which produces oscillations of 2-3X in amplitude (that decay over time as the model is deterministic)" is misleading. Determinism alone does not preclude sustained oscillations e.g., the repressilator is a deterministic oscillator. The decay observed here is likely due to the system's parameters leading to damped dynamics.

We apologise for this error and have amended the text on page 16.

5. "Repress mRNA of the other gene by 50%", I think the authors mean "Repress mRNA transcription rate of the other gene by 50%".

Yes, we have edited the text on page 9.

Second decision letter

MS ID#: dev.204969R1

MS TITLE: Oscillatory Co-expression of HES1 and HES5 Enables a Hybrid State in a Cross-repressive Transcription Factor Regulatory Motif

AUTHORS: Veronica Biga, Anzy Miller, Anoushka Kamath, Robert Lea, Ying Q. P. Mak, Antony D. Adamson, Elli Marinopoulou, Paul Francois, Nancy Papalopulu and Cerys S. Manning

Dear Dr Manning,

I am happy to tell you that your manuscript has been accepted for publication in Development, pending our standard publication integrity checks. It was accepted on 25 Apr 2026. Where referee reports on this version are available, they are appended below. You will see that Reviewer 2 would like some additional information, but I am content for you to deal with this in proofs.

Reviewer 1

© 2026. Published by The Company of Biologists under the terms of the Creative Commons Attribution License (https://creativecommons.org/licenses/by/4.0/).

Advance summary and potential significance to field

Biga et al. focus on Hes1 and Hes5 and use a combination of advanced live cell imaging and computational models to demonstrate that their oscillatory expression facilitates a hybrid state in which these cross-repressive cell fate determinants can be co-expressed in the same cells and that the oscilliatory regime can be resolved into either Hes1 or Hes5 single positive states in response to changes in levels and/or cross-repression threshold between the TFs. The authors lastly provide evidence that this transition from double-positive Hes1/Hes5 hybrid state to segregated single-positive Hes1 and Hes5 expression domains occurs in the embryonic spinal cord.
Altogether, this is an interesting, well-written and logically structured manuscript that makes a significant and novel mechanistic contribution to our understanding how oscillations can maintain hybrid states by allowign co-expression of otherwise antagonizing cell fate determinants, and thus developmental plasticity, and based on which parameters these hybrid states can resolve into single positive states. The data is convincing and the inferences made from the models are clearly explained. The authors have also addressed all my comments from the initial round of review. As transcription factor oscillations play essential roles in numerous developmental and also other processes, this manuscript is an excellent fit for publication in Development.

Reviewer 2

Advance summary and potential significance to field

The authors have addressed all reviewer comments in depth.

In the siRNA experiments (new Figure 4F, G): Efficiency of knockdown is not obvious from the quantifications: How strong was the knockdown per cell, and in how many cells was it visible? In bulk it was 30 - 60 %. The authors indicate in the main text that Hes1 knockdown was not visible in the NPC cultures. Also in new Fig. 4, a knockdown is not necessarily obvious. It would make sense to analyse this not in bulk but on a single-cell basis: Would the results be different, if only cells with knockdown (i.e., low/decreasing levels in Hes1 or Hes5, respectively) were analysed? And if there is no knockdown in NPCs, then there is no knockdown.
It would be good to either clarify in the text how the analysis was done or adjust the analysis accordingly.

Reviewer 3

Advance summary and potential significance to field

The authors have done a great job at addressing satisfactorily the comments raised by the reviewers.

© 2026. Published by The Company of Biologists under the terms of the Creative Commons Attribution License (https://creativecommons.org/licenses/by/4.0/).
